# Catalyzing satellite communication: A 20W Ku-Band RF front-end power amplifier design and deployment

**DOI:** 10.1371/journal.pone.0300616

**Published:** 2024-04-10

**Authors:** Jiafa Chen, Fei Wang, Dawei Zhang, Jinsong Liu, Huaxia Wu, Zhengxian Zhou, Haima Yang, Tingzhen Yan, Tianchen Tang

**Affiliations:** 1 Department of Research Center of Optical Instrument and System, Ministry of Education and Shanghai Key Lab of Modern Optical System, University of Shanghai for Science and Technology, Shanghai, China; 2 Department of China Aviation East China Optoelectronics, Anhui East China Photoelectric Technology Research Institute, Wuhu, Anhui Province, China; 3 Department of College of Physics and Electronic Information, Anhui Normal University, Wuhu, Anhui Province, China; 4 Department of Optoelectronic Materials Science and Technology, Anhui Provincial Key Laboratory, Wuhu, Anhui Province, China; 5 Department of Printing and pack aging Engineering, Shanghai Publishing and Printing College, Shanghai, China; Virginia Military Institute, UNITED STATES

## Abstract

This paper presents a groundbreaking Ku-band 20W RF front-end power amplifier (PA), designed to address numerous challenges encountered by satellite communication systems, including those pertaining to stability, linearity, cost, and size. The manuscript commences with an exhaustive discussion of system design and operational principles, emphasizing the intricacies of low-noise amplification, and incorporating key considerations such as noise factors, stability analysis, gain, and gain flatness. Subsequently, an in-depth study is conducted on various components of the RF chain, including the pre-amplification module, driver-amplification module, and final-stage amplification module. The holistic design extends to the inclusion of the display and control unit, featuring the power-control module, monitoring module, and overall layout design of the PA. It is meticulously tailored to meet the specific demands of satellite communication. Following this, a thorough exploration of electromagnetic simulation and measurement results ensues, providing validation for the precision and reliability of the proposed design. Finally, the feasibility of that design is substantiated through systematic system design, prototype production, and exhaustive experimental testing. It is noteworthy that, in the space-simulation environmental test, emphasis is placed on the excellent performance of the Star Ku-band PA within the 13.75GHz to 14.5GHz frequency range. Detailed power scan measurements reveal a P_1dB_ of 43dBm, maintaining output power flatness < ± 0.5dBm across the entire frequency and temperature spectrum. Third-order intermodulation scan measurements indicate a third-order intermodulation of ≤ -23dBc. Detailed results of power monitoring demonstrate a range from +18dBm to +54dBm. Scans of spurious suppression and harmonic suppression, meanwhile, show that the PA evinces spurious suppression ≤ -65dBc and harmonic suppression ≤ -60dBc. Rigorous phase-scan measurements exhibit a phase-shift adjustment range of 0° to 360°, with a step of 5.625°, and a phase-shift accuracy of 0.5dB. Detailed data from gain-scan measurements show a gain-adjustment range of 0dB to 30dB, with a gain flatness of ± 0.5dB. Attenuation error is ≤ 1%. These test parameters perfectly align with the practical application requirements of the technical specifications. When compared to existing Ku-band PAs, our design reflects a deeper consideration of specific requirements in satellite communication, ensuring its outstanding performance and uniqueness. This PA features good stability, high linearity, low cost, and compact modularity, ensuring continuous and stable power output. These features position the proposed system as a leader within the market. Successful orbital deployment not only validates its operational stability; it also makes a significant contribution to the advancement of China’s satellite PA technology, generating positive socio-economic benefits.

## Introduction

Recently, the deployment of communication satellites in low Earth orbit has emerged as a significant global research focus. Additionally, low Earth-orbit satellites, governed by a “first-come-first-served” rule, represent a renewable and strategic asset, influencing the future developmental prospects of nations worldwide. Consequently, competition is intense among countries in the field of near-Earth communication [1–5]. The satellite-based communication system, characterized by satellite-ground interaction, is extensively used, not only in satellite communication *per se*, but also in remote-sensing measurements, and various other fields [6]. The Ku-Band, known for its extended signal-transmission range and robust anti-interference capability, is widely employed in satellite-based communications [7–11]. The power amplifier (PA), a critical component in the satellite-based communication system, is primarily employed for amplifying and transmitting high-power RF signals. Furthermore, its performance and stability directly influence the overall effectiveness of the communication system [12–16]. Therefore, the design and implementation of PAs evince immense significance and practical value.

Currently, star-communication technology is advancing rapidly, imposing higher requirements, but also offering broader application prospects for RF PA design [17–19]. Researchers worldwide have extensively investigated RF PA design in the field of satellite communication [20, 21]. Common PA-design models include microwave tubes, semiconductor devices, and integrated circuits [22–27]. While microwave-tube PAs offer good linearity and stability, they are larger, consume more power, and incur higher maintenance costs [28, 29]. Additionally, while semiconductor-device PAs entail advantages such as small size and low power consumption, their linearity and stability evince limitations related to device characteristics [30, 31]. Thus, improving the reliability and maintenance of the PA, while ensuring power output and signal stability, is a key focus.

Meanwhile, owing to challenges in current PA research, particularly around modular design, researchers have proposed various new ideas and approaches, such as the design concept of the combination of discrete and integrated modules. These approaches can enhance the balance of power amplification, matching, and stability considerations [32–36]. Research on low-noise techniques and modulation approaches for PAs has also garnered significant attention [37–40]. Foreign companies, led by STMicroelectronics, have achieved considerable success in the field of PAs, with their widely used products establishing them as international leaders due to their excellent performance. In China, research in this field is also gradually emerging, although the PAs developed using domestic gallium nitride (GaN) chips currently manifest low efficiency, low linearity, significant power drops, and poor reliability under continuous-wave high and low temperature conditions. Meanwhile, all pertinent indices evince more than 10% difference, and this cannot meet the demand associated with high-power amplifiers for high-performance satellite communication [41–47]. In the future, the requirements of RF PAs will continue to increase, as star technology further advances. Therefore, improving power-output efficiency, reducing power consumption, increasing reliability, and reducing noise will become significant topics in future RF PA research. Meanwhile, the development of more intelligent control systems, and more accurate simulation and testing techniques, will provide better support for RF PA research [48–50].

This paper addresses the specific requirements of satellite communication by introducing a modular, low-noise Ku-Band 20W RF transmit front-end PA. The primary objective is to enhance the quality and reliability of satellite communication, thereby contributing significantly to the advancement of satellite-communication technology. Through in-depth research, and a meticulous analysis of PA circuit principles, system parameters, and component selection, the system is designed for integration. The integration process leverages innovative, independently researched and developed GaN microwave power devices, with a focus on enhancing power output and linearity. This strategic approach successfully resolves issues related to poor linearity, low efficiency, and inadequate stability. Furthermore, it establishes a rational and achievable technical and process implementation plan, permitting the realization of multi-functional power amplification, high modularity, stable power output, and enhanced reliability. Ultimately, indeed, this work establishes comprehensive power-amplification technical indices, applicable to practical engineering projects.

## System design and working principle

**Fig 1:**
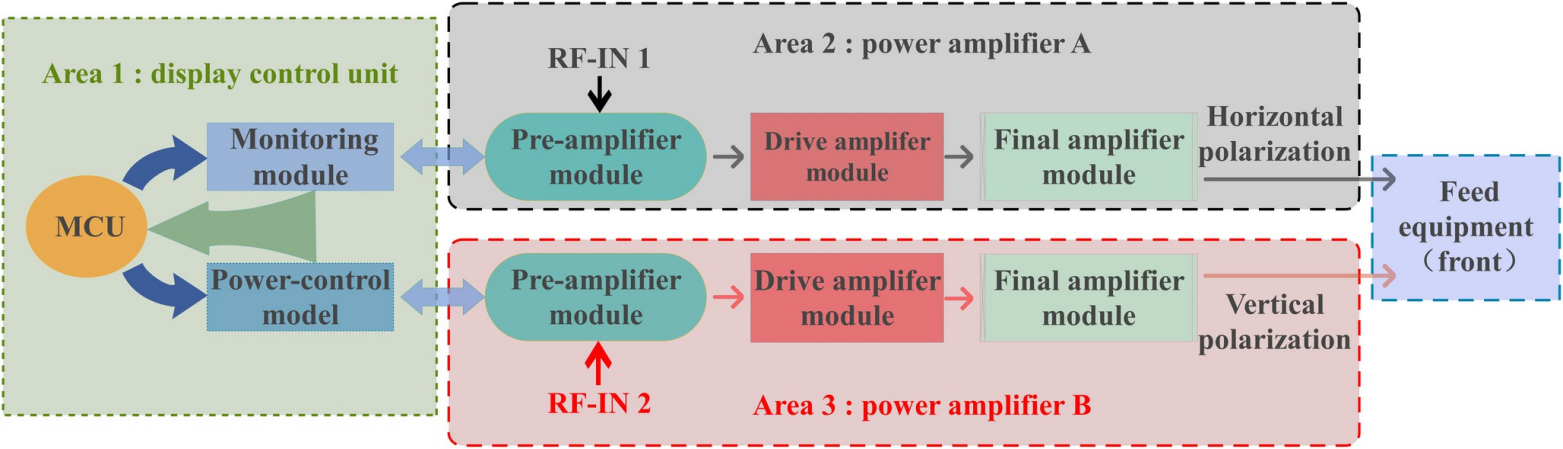
Topological diagram of 20W RF transmit front-end PA for Star Ku-Band.

The Ku-Band satellite transmission signal proposed in this paper is a linearly polarized electromagnetic wave, with a specific polarization angle. It is based on the principle of vector signal synthesis decomposition [51, 52]. This allows the decomposition of the phase and amplitude of the Ku linearly polarized electromagnetic wave into vertical and horizontal linear-polarization channels. The topology of the 20W RF transmit front-end PA for the Star Ku-Band is illustrated in Fig 1. The system utilizes spatial combination technology, connecting the display control unit, comprising a microcontroller (MCU), power-control module, monitoring module, and two amplifiers with identical power output. Additionally, it features a modular design, enhancing maintainability and facilitating mass production. This design reduces system losses, significantly improves output power and efficiency, and increases Ku-Band power amplification from 13.75GHz to 14.5GHz. The system has successfully achieved power amplification, out-of-band filtering, gain adjustment, polarization phase shift, beam phase shift, and the detector monitoring of Ku-Band RF signals. Simultaneously, it has achieved the tracking and transmission of Ku-Band satellite-communication signals and power amplification.

Fig 1 illustrates a dual-channel RF link with identical power output. The Ku-Band transmission signal undergoes initial processing in a preamplifier module, the latter comprising an attenuator, a CNC phase shifter, a low-noise amplifier, and a filter. This conversion and correction process permits both horizontal and vertical polarization. Adjustments to the beam phase shift and the correction of phase shifts between array elements are also realized. Subsequently, drive amplification of the transmit signal is achieved through the drive amplifier module, which consists of a high linear drive chip and an attenuator. Finally, signal-power amplification is achieved through the final-stage amplifier module. This module encompasses an amplifier chip, microstrip-waveguide conversion, isolation filter components, harmonic component suppression, one-way output, and an improvement of output standing waves. The power-control module primarily converts the input DC +24V power into different voltages, ensuring the necessary voltage and current for the RF link and the active chips in the monitoring module. The monitoring module is comprised of an attenuator, phase shifter, temperature and power monitoring components, and fan control, via the internal MCU chip, completing chip control. Externally, it can communicate with the beam-control module via the RS232 serial port, providing operational-status parameters. Additionally, it performs interactive control, internal configuration, monitoring, and module-alarm functions. The main technical indices of its system design are presented in Table 1.

**Table 1:** Main technical indices of its system design.

Indicators	Technical requirements
**Operating frequency (F**_**0**_ **)**	13.75GHz—14.5GHz
**Input power**	-10dBm - 0dBm
**Peak output power (Psat)**	≥ 20W
**Output power flatness**	≤ 0.5dBm
**Output P**_**1dB**_ **power**	≥ 43dBm (single-load continuous wave signal test)
**Third order syncopation**	≤ -23dBc (offset 20MHz dual carrier combined output 38dBm test)
**Gain adjustment**	0dB-30dB, steps of 0.5dB, gain flatness ≤ 2dB, attenuation error ≤1%
**Phase shift regulation**	0°-360°, steps of 5.625°, phase shift accuracy of 0.5dB
**Stray suppression**	≤ -65dBc
**Harmonic suppression**	≤ -60dBc
**Port standing waves**	≤ 1.5
**Monitoring communication method**	Ethernet port/RS-485
**Operating temperature**	-40°C ~ -60°C
**Amplifier protection function**	Over-current, over-excitation, over-reflection, over-temperature and other protection functions

### Low-noise amplification design

The microwave low-noise amplifier is a critical component in radar, electronic countermeasures, and telemetry remote-control receiving systems [53, 54]. The noise in the transmitting system is heavily influenced by the noise of the amplifier, with the noise coefficient of the preamplifier module exerting the most significant impact on the overall noise of the microwave system. Additionally, its gain determines the extent of noise suppression in the subsequent circuit. This highlights the fact that the performance of the low-noise amplifier constrains the overall performance of the transmitting system. It also significantly contributes to enhancing the overall technical level of the system [55–58]. Therefore, the fundamental requirements for low-noise amplifiers include a low-noise figure, sufficient power gain, good operating stability, and a large dynamic range [59, 60].

#### Noise factor

The noise factor (NF) of a low-noise amplifier serves as a crucial indicator, encompassing the comprehensive noise performance of the entire receiving front-end system, and exerting a direct influence on the sensitivity of that system [61]. More precisely, the total noise factor of a low-noise amplifier is characterized by the ratio of the input signal-to-noise ratio to the output signal-to-noise ratio. This metric provides valuable insights into the ability of the amplifier to maintain signal integrity and minimize noise, thereby playing a pivotal role in optimizing the performance of the receiving front-end system.

$$\phi =\frac{S_{in}/N_{in}}{S_{out}/N_{out}}$$ (1)

Where, *S*_*in*_ is the input signal power, *N*_*in*_ is the input noise power, *S*_*out*_ is the output signal power and *N*_*out*_ is the output noise power.

$$S_{out}=G_{1}S_{in}$$ (2)

$$N_{out}=N_{1}+G_{1}N_{in}$$ (3)

where *N*_1_ is the noise inherent in the first-stage amplifier, and *G*_1_ is the gain of the first-stage amplifier, which can be further derived as follows:

$$\phi =\frac{S_{in}/N_{in}}{G_{1}S_{in}/\left(N_{1}+G_{1}N_{in}\right)}=1+\frac{N_{1}}{G_{1}N_{in}}$$ (4)

Therefore, the total noise factor of the two-stage cascade amplifier can be derived thus:

$$\phi =\frac{S_{in}/N_{in}}{G_{1}G_{2}S_{in}/\left(N_{2}+N_{1}G_{2}+G_{1}G_{2}N_{in}\right)}=1+\frac{N_{1}}{G_{1}N_{in}}+\frac{N_{2}}{G_{1}G_{2}N_{in}}=\phi_{1}+\frac{\phi_{2}-1}{G_{1}}$$ (5)

**Fig 2:**
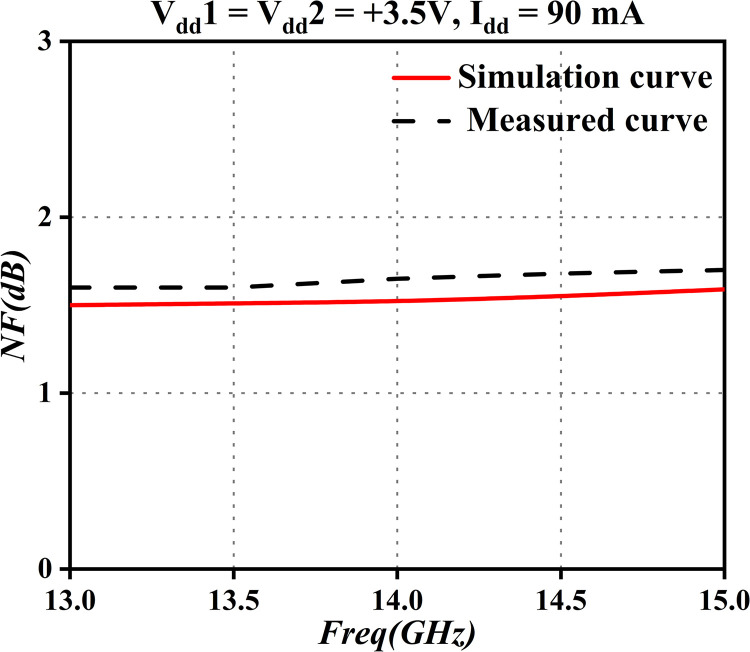
NF curve.

*G*_2_, *N*_2_ are the noise and gain of the second-stage amplifier, respectively. *ϕ*_1_ is the noise factor of the first stage, and *ϕ*_1_ is the noise factor of the second. The overall noise factor in a cascade configuration is mainly determined by the performance of the first-stage low-noise amplifier. A smaller noise factor, and larger gain, lead to a reduced overall circuit noise factor. Subsequent stages must primarily consider gain and power capacity. In the design of input matching circuits, any passive component, such as resistors, capacitors, and transmission lines, will directly degrade the overall circuit noise factor, according to this formula. Furthermore, the fundamental principle of input matching design for low-noise amplifiers involves conjugately matching the optimal noise impedance (Γopt) of the device to 50 ohms, using a minimal number of passive components. Therefore, we conducted simulations and relevant tests on the noise factor of the first-stage amplifier. Specific results are presented in Fig 2. This design concept aims to ensure that the entire system maintains optimal noise performance during input matching, offering robust support for the efficient operation of the overall circuit.

Illustrated in Fig 2, the NF typically measures 1.6dB, reflecting favorable noise characteristics. Nonetheless, within the frequency range of 13.75GHz to 14.5GHz, the measured noise figure exceeds the simulated value. In practical low-noise design considerations, pursuit of the lowest noise figure may result in significant port standing wave issues. Particularly when cascaded with a transmitting band-pass filter, moreover, the total noise figure may significantly exceed the theoretically calculated value. This phenomenon, mainly attributable to transmission-line losses and radiation losses caused by discontinuities, results in a noise figure slightly lower than the theoretical value, but still in compliance with the technical specifications outlined in this paper. In practical applications, finding a balance between the lowest noise figure and port standing waves is crucial to ensure the reliability and performance of the system, in accordance with the design specifications.

#### Stability analysis

If the amplifier enters an unstable state, it may trigger self-oscillation of the signal, resulting in a rapid change in the amplifier gain and, ultimately, leading to transistor burnout [24]. Thus, in amplifier design, our objective is to maximize operational stability. In essence, in fact, our aim is to design an amplifier capable of stable operation under various impedance conditions, thus avoiding self-oscillation. This design principle aims to guarantee the reliability of the amplifier, sustaining stable performance across diverse operating conditions to minimize the risk of damage and failure.

$$K=\frac{1-{\left|S_{11}\right|}^{2}-{\left|S_{22}\right|}^{2}+{\left|\Delta \right|}^{2}}{2|S_{12}||S_{21}|}>1$$ (6)

$$|\Delta |=|S_{11}S_{22}-S_{12}S_{21}|<1$$ (7)

Where K is the StabFact of the test process, i.e., the stability coefficient. Λ is the reflection coefficient, and *S*_*ij*_ is the scattering parameter of the two-port network of *S*. This is mainly used to judge the stable state of the whole system. When K is greater than 1, it is in a stable state; if K is less than 1, conversely, it is in an unstable state.


$$1-{\left|S_{11}\right|}^{2}>|S_{12}||S_{21}|$$ (8)

$$1-{\left|S_{22}\right|}^{2}>|S_{12}||S_{21}|$$ (9)

**Fig 3:**
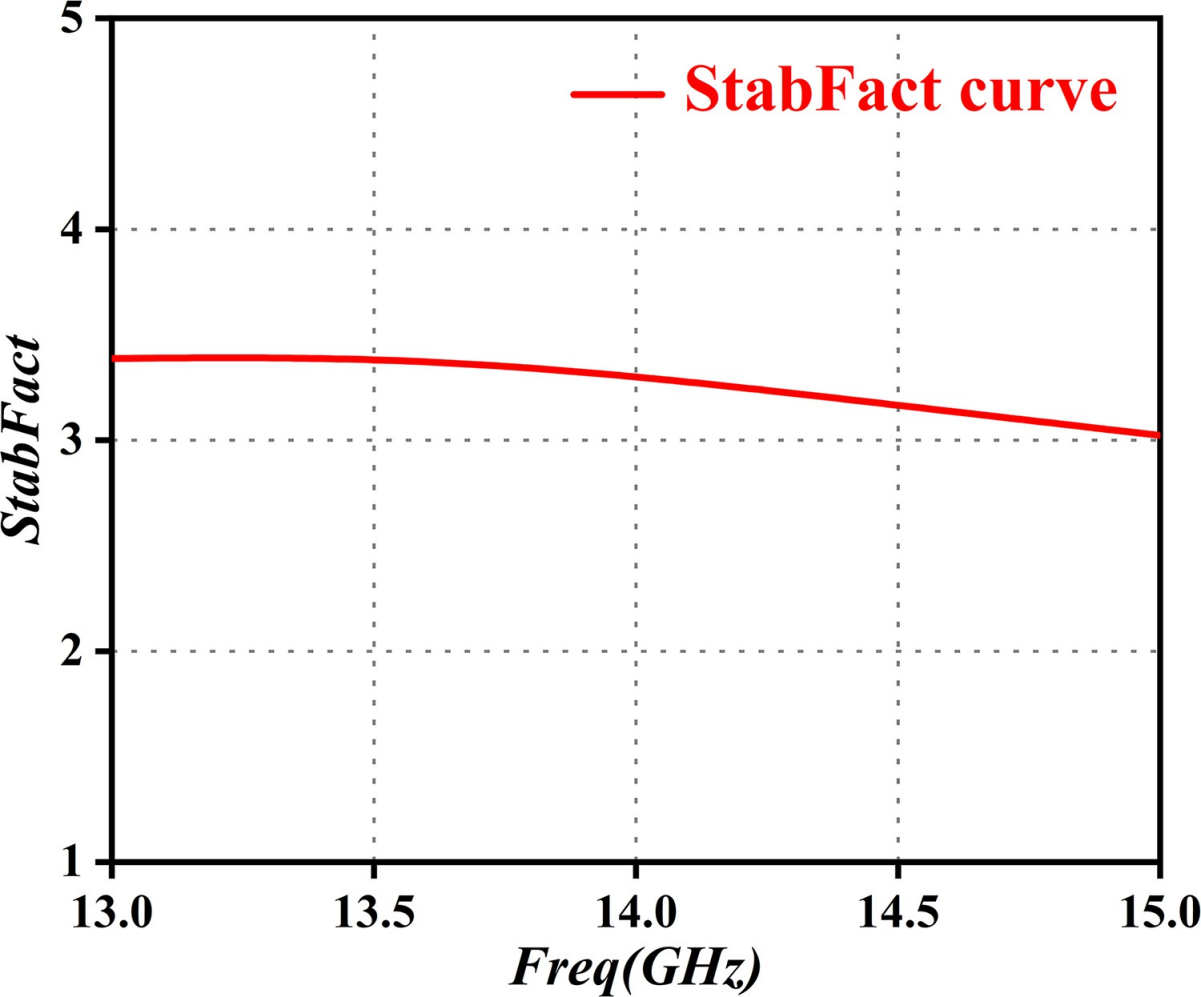
StabFact curve.

Low-noise amplifiers can achieve absolute stability only when the four inequalities mentioned above are satisfied. Hence, in the early design stages, calculations must assess the stability of the amplifier. This process simplifies the selection of appropriate transistor types, while compensating for the real part of the input impedance, using series resistors. Thus, the maintenance of transistor stability requires the real part of the input impedance to be greater than zero, indicating a reflection coefficient less than 1. In this study, we used matching reactive components to guarantee amplifier stability. Following completion of the layout design, stability coefficient simulations were conducted using ADS, as depicted in Fig 3. This process helps validate the stability of the design, while offering strong support for subsequent work.

In view of the simulation results, shown in Fig 3, we determined that the StabFact exceeds 1 within the frequency range of 13.75GHz to 14.5GHz. This guarantees the absolute stability of the amplifier across the frequency band, aligning with the technical specifications of the low-noise amplifier design in this paper. This result robustly substantiates the calculations and assessments of amplifier stability in our previous work, establishing a reliable theoretical foundation for achieving optimal performance within a specific frequency range in the designed system.

#### Gain and gain flatness

In the design of low-noise amplifier circuits, three power gains are commonly used: transmission power gain *G*_*t*_, operating power gain *G*_*p*_, and utilization power gain *G*_*a*_ [62]. The relationship between the three power gains is as follows.

$$G_{t}=\frac{P_{1}}{P_{2}}=\frac{P_{1}}{P_{in}}\frac{P_{in}}{P_{2}}=G_{p}\times M_{1}$$ (10)

$$G_{t}=\frac{P_{1}}{P_{2}}=\frac{P_{1}}{P_{3}}\frac{P_{3}}{P_{2}}=G_{p}\times M_{2}$$ (11)

Where *P*_1_ is the absorbed power of the load; *P*_*in*_ is the input power; *P*_2_ is the capital-power output from the signal source; *P*_3_ is the capital power absorbed by the load; and *M*_1_, *M*_2_ are the mismatch coefficients of the input and output, respectively. When the amplifiers are conjugate matched, the following is obtained:

$$M_{1}=M_{2}$$ (12)

$$G_{t}=G_{a}=G_{p}$$ (13)

**Fig 4:**
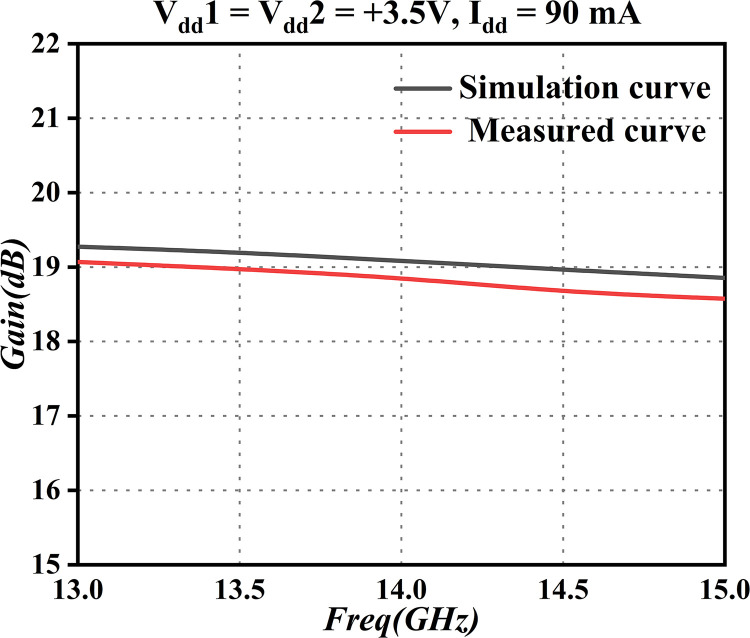
Gain curve.

The gain flatness indicates the fluctuation of the gain, i.e., Δ*G* = *G*_max_−*G*_m*in*_. A consistent gain flatness is crucial in alleviating challenges in subsequent amplifier designs. To enhance the credibility of our proposed design, we employed Advanced Design System (ADS) for simulations and conducted pertinent tests on the gain, as illustrated in Fig 4. This rigorous process ensured that the system maintained uniform gain levels across various frequencies, establishing a stable foundation for overall system performance. The thorough simulation and testing procedures enabled a comprehensive evaluation of the effectiveness of the proposed solution, thereby offering compelling support for its practical applications.

In analyzing Fig 4, we may observe that, within the frequency range of 13.75GHz to 14.5GHz, the typical gain of the amplifier is 19dBm, and it maintains a gain flatness of ± 0.5dB. This suggests that the low-noise amplifier excels in terms of gain performance. Nonetheless, a slight decrease in gain within this frequency band is observed in the test results, compared to the simulation. The main reason is a minor inaccuracy in the modeling of relevant losses, resulting in a slightly smaller measured attenuation as compared to the simulation. This result still aligns with the practical technical specifications of this paper, however.

As indicated by the data in Table 2, the low-noise amplifier designed for this paper exhibits outstanding comparative performance in terms of noise factor, stability, and gain flatness, demonstrating significant superiority. These advantages provide excellent prospects for the design within practical application scenarios. We thus anticipate that our work will contribute significantly to the enhancement, and broader application, of low-noise amplifiers in the current field.

**Table 2:** Performance comparison of low-noise amplifiers.

Technical index	[63]	[64]	[65]	This work
**Freq (GHz)**	2–20	4–20	13–16	13.75–14.5
**NF (dB)**	3.2	1.6	1	1.6
**Gain(dB)**	18	24	22.8	19
**Gain Flatness (dB)**	±1	±1.25	±6	±0.5

## Module design

### RF link

#### Preamplifier module

**Fig 5:**
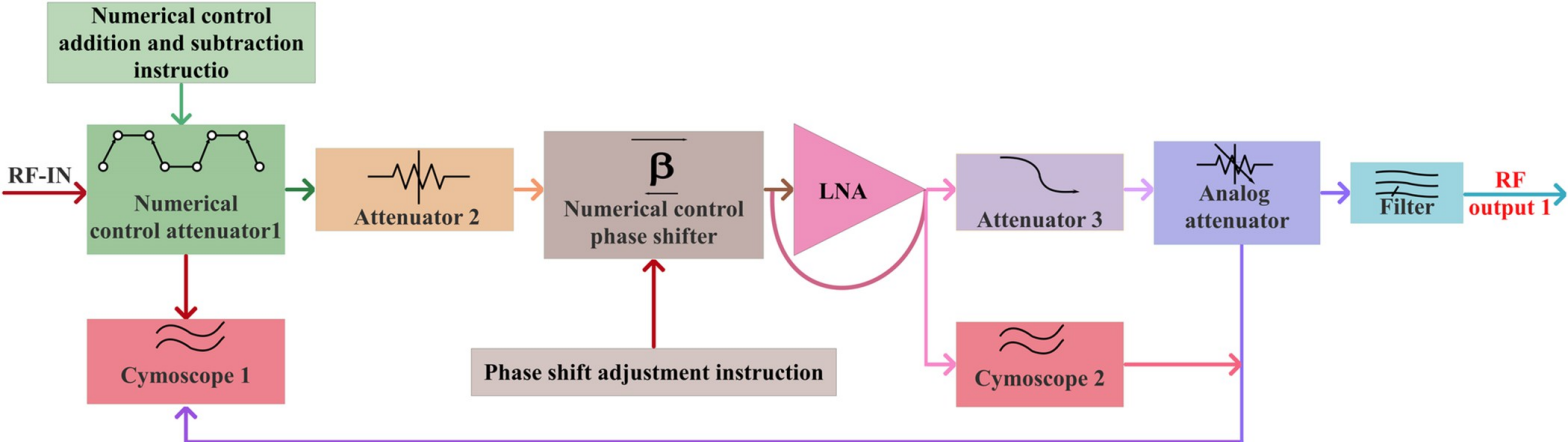
Block diagram of single-channel preamplifier module.

Fig 5 shows the block diagram for the single-channel preamplifier module in the RF link of the Ku-Band RF transmit front-end PA, as designed for star applications. Key components encompass a detector, attenuator, CNC phase shifter, low-noise amplifier, and filter. The preamplifier link consolidates functions such as link-gain compensation, low-noise amplification, amplitude adjustment, over-excitation automatic control, gain transmission, polarization-phase shift adjustment, and harmonic suppression, into a single module. Consequently, the module possesses characteristics of high integration, miniaturization, and multi-functional fusion.

**Fig 6:**
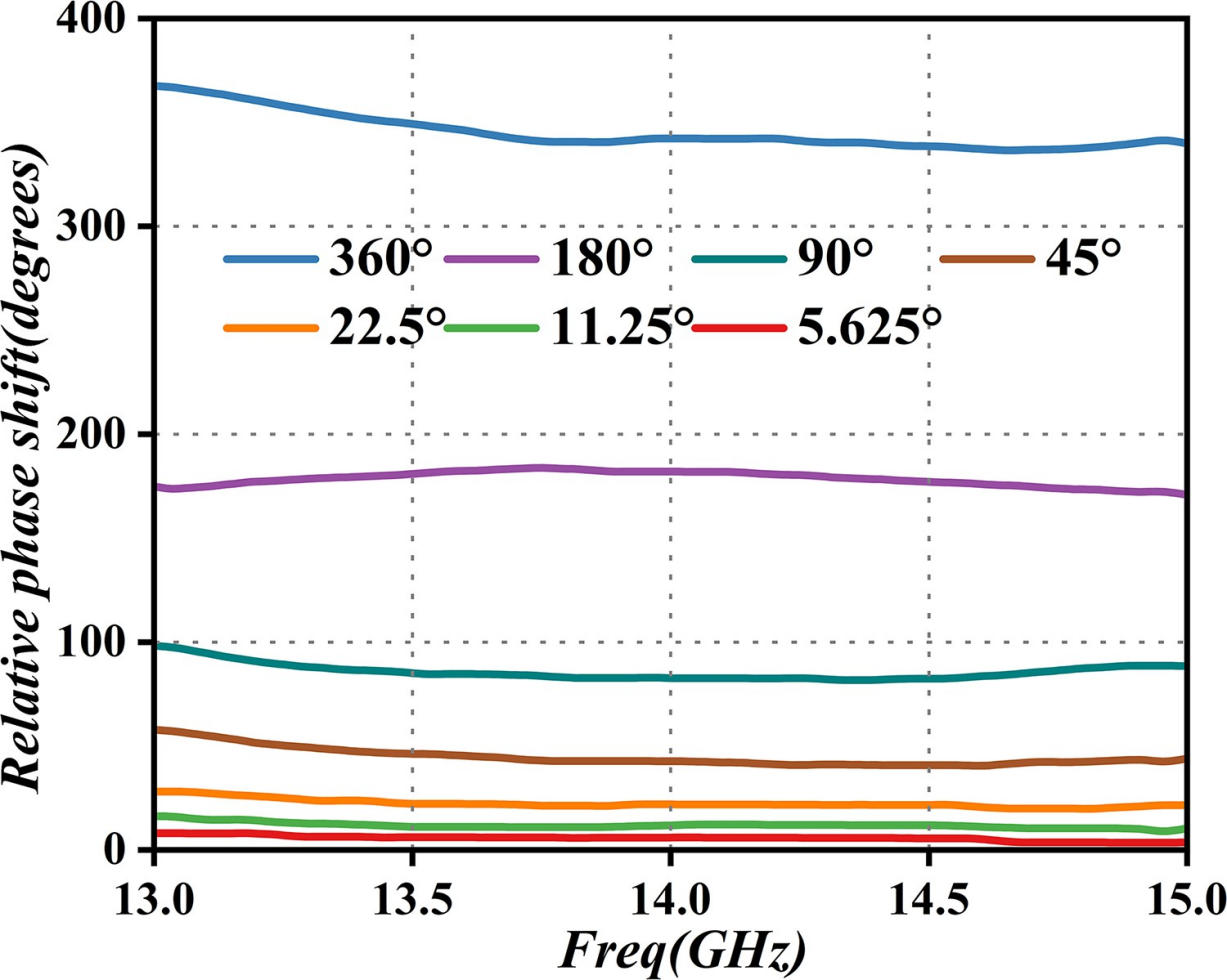
Phase-shift performance simulation curve.

The module includes four levels of attenuators for both the horizontal and vertical polarization channels. The first two levels of the attenuation system include a numerical-control attenuator and a second-level attenuator. These are controlled in response to external input-gain numerical-control plus and minus commands, to meet the amplitude requirements of the horizontal and vertical branches of the polarization angle. Furthermore, this facilitates the closed-loop adjustment of the signal amplitude in the two channels. The second two levels of the attenuation system consist of a third attenuator and an analog attenuator. The digital phase shifter in the system module on the horizontal and vertical polarization channels can respond to external input-phase shift-adjustment commands, achieving a 90° orthogonal phase shift of the horizontal and vertical channels. This enhances polarization isolation, thereby improving the performance simulation of the phase shift. Fig 6 illustrates the primary performance simulation of the phase shift.

**Fig 7:**
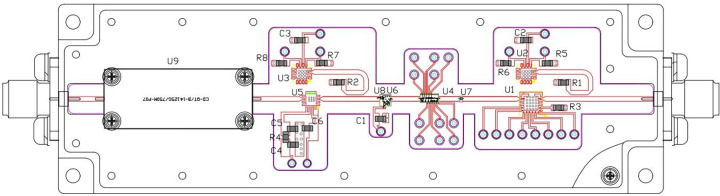
Schematic diagram of the preamplifier module.

As presented in Fig 6, the phase-shift unit can achieve phase adjustment in the range of 0°-360°, with a step size of 5.625°, and its phase-shifting accuracy is 0.5dB. Additionally, the phase of the transmission channel for each component in the phased array is compensated and corrected. The correction value is derived from the offset value of the beam shifter in the receiving channel and adjusted based on its size. Hence, the configuration value of the phase-shift unit is the sum of the polarization phase-shift parameter 1, and the array element inter-beam phase-shift parameter 2. This component also includes a low-noise amplifier stage to achieve a gain amplification of 20dB and a low power output of 7dBm. This is crucial for driving the driver stage. The principle of the single-channel module for this stage is illustrated in Fig 7 (Schematic, layout, and dimensional drawings are described in detail in S2, S11, S15, S19, S24, S25 Figs in S1 Raw images).

As shown in Fig 7, the entire single-channel preamplifier module uses more mature friction welding, and other process technologies, to sinter the RF link in the module and physically isolate it with a special isolation pressure bar, thereby reducing interference. Through rational arrangement of the circuit structure, the circuit layout can be optimized, improving both the efficiency and miniaturization of the module. Simultaneously, this accomplishes Ku-band signal-power adjustment and polarization, beam phase-shift adjustment, vertical and horizontal polarization conversion and correction, phase-shift correction between array elements, power amplification, and suppression of harmonics and out-of-band interference signals. Consequently, the preamplifier is characterized by its small size, low power consumption, high reliability, excellent performance, flexible configuration, and high stability.

#### Drive amplification module

**Fig 8:**
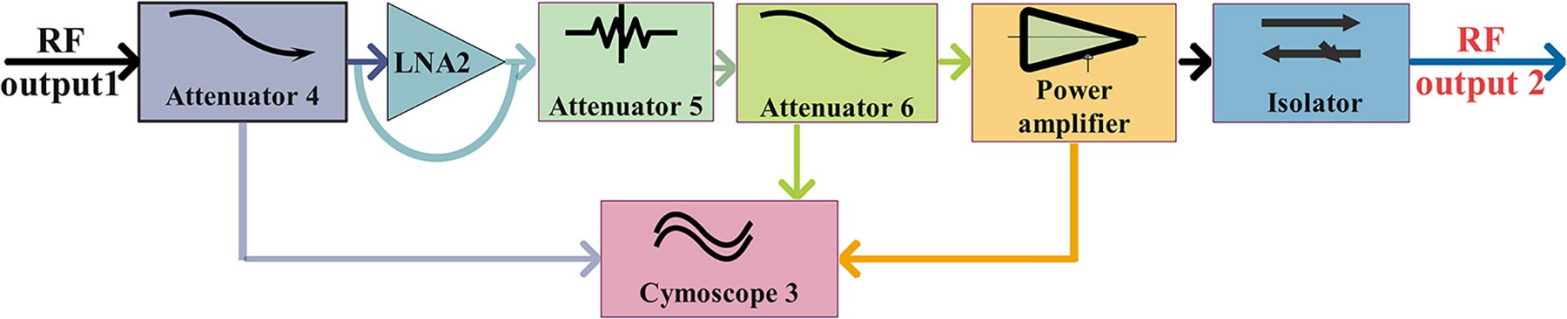
Block diagram of drive-amplification module.

**Fig 9:**
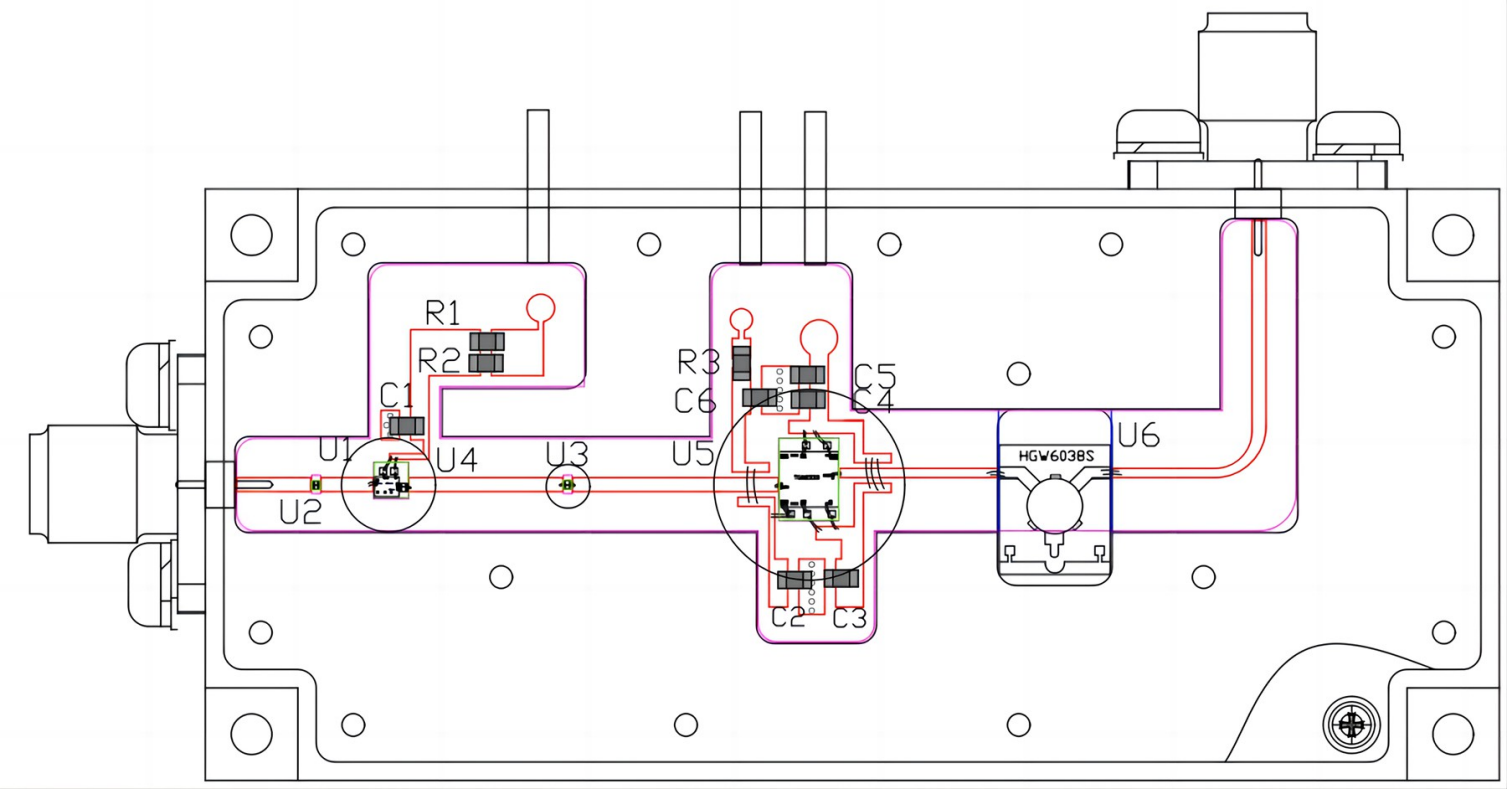
Drive-amplifier module schematic.

Fig 8 illustrates the block diagram of a single-channel driver-amplifier module, primarily consisting of a low-noise amplifier, attenuator, driver-amplifier chip, and isolator. This module accomplishes the transmission and power amplification of the RF signal output from the preamplifier module, ensuring the normal operation of the final amplifier module. The RF signal output from the pre-stage amplifier passes through the fourth attenuator in this module, thus ensuring satisfaction of the input requirements of the driver amplifier. Simultaneously, the signal undergoes additional amplification by the low-noise amplifier. After passing through the fifth and sixth attenuators to address port standing wave issues, caused by the digital IC chip and low-noise amplifier, the signal is ultimately amplified using the linear-driver chip. Moreover, to prevent the reflection of high power from the final stage back to the driver-amplifier module (which can lead to oscillation and damage to the driver amplifier), an isolator can be used to separate the driver-amplifier module from the final-stage amplifier module. The fundamental driver-amplifier module is depicted in Fig 9 (Schematic, layout, and dimensional drawings are described in detail in S3, S12, S16, S20, S26, S27 Figs in S1 Raw images).

**Fig 10:**
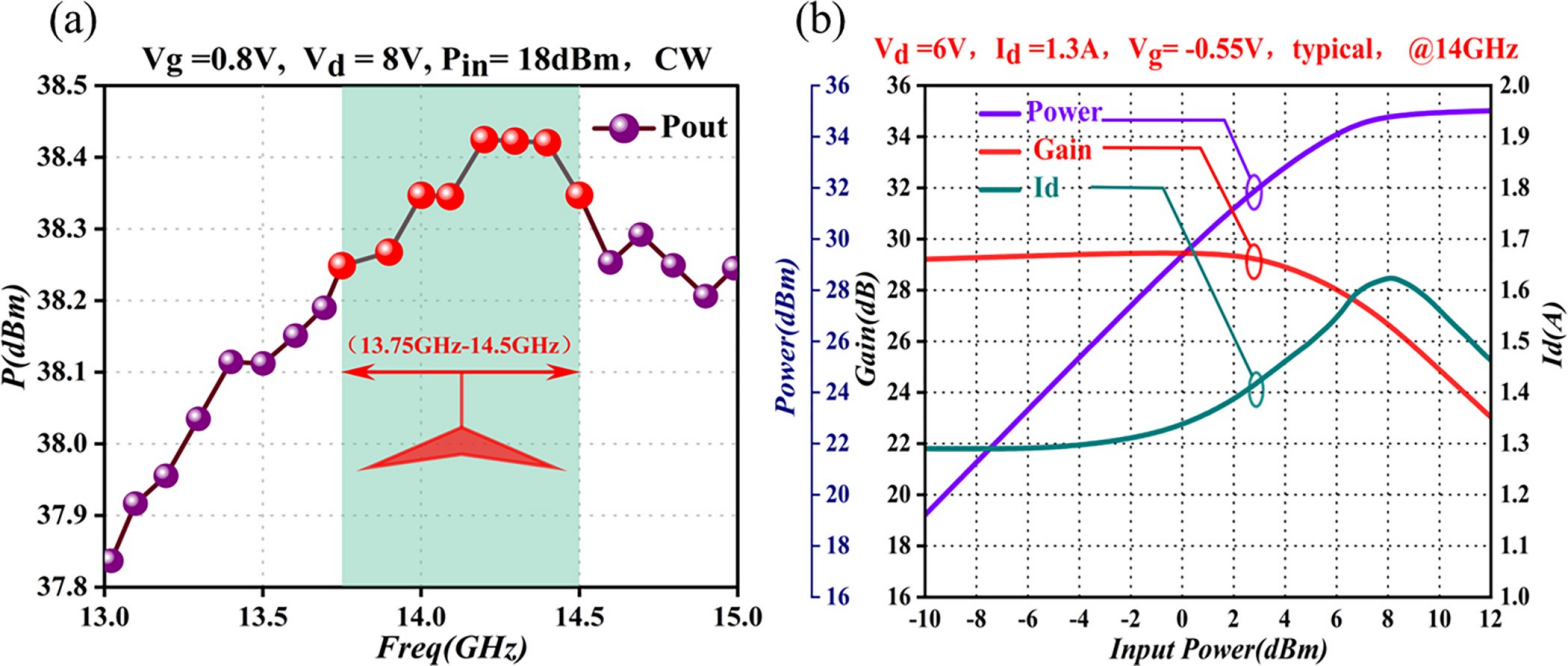
Output-performance curve of the driver-amplifier module. (a) Frequency-output power graph (b) Input power-output power/gain/Id graph.

Given the low output power of the preamplifier and the high output power of the final amplifier, maintenance of a linear operating state for the driver-amplifier module in between is crucial. Consequently, to mitigate crossover distortion, we conducted tests on the output performance of the module, generating its output-performance graph (see Fig 10).

Fig 10(A) shows that the module delivers over 38dBm of output power within the 13.5GHz to 14.5GHz frequency band. Simultaneously, in Fig 10(B), the graph depicts the modular output power as increasing with input power in the linear operational state, reaching over 34dBm. Moreover, it achieves a power gain of >28dB with a current draw of < 1.7A. The module exhibits excellent linearity. With operation at P_1dB_, employment of a power-fallback method results in a 7dB fallback above the threshold, ensuring no further reduction in the linearity index of the RF link.

#### Final stage amplification module

**Fig 11:**
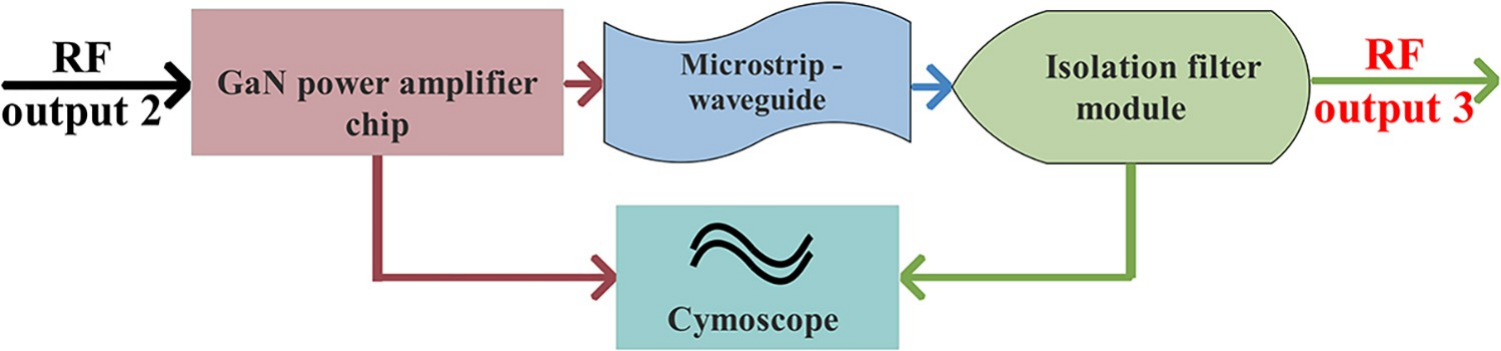
Block diagram of the final-stage amplification module.

The final-stage amplifier module, a crucial component of the system, is designed to enhance the characteristics of the PA. It plays a key role in amplifying the signal transmitted from the driver-amplifier module, in order to generate a high-power signal. This module encompasses a GaN chip, microstrip-waveguide converter, detector, and isolation filter (see Fig 11).

This module aims to utilize 0.25um GaN HEMT process technology, gain compression and expansion technology, and bias-adaptive dynamic adjustment on gate-voltage technology, in the domain of high linear continuous-wave amplification. Consequently, it enhances the autonomous controllable development process of GaN chips. These chips are collaboratively developed to achieve a high-power Ku-Band amplifier through pre-distortion, efficient spatial synthesis, and other technologies. Experimental results demonstrate that the chip exhibits good linearity and high efficiency, meeting the RF output-power technical index requirements for transmitting front-end power amplification in the Ku-Band for star use.

**Fig 12:**
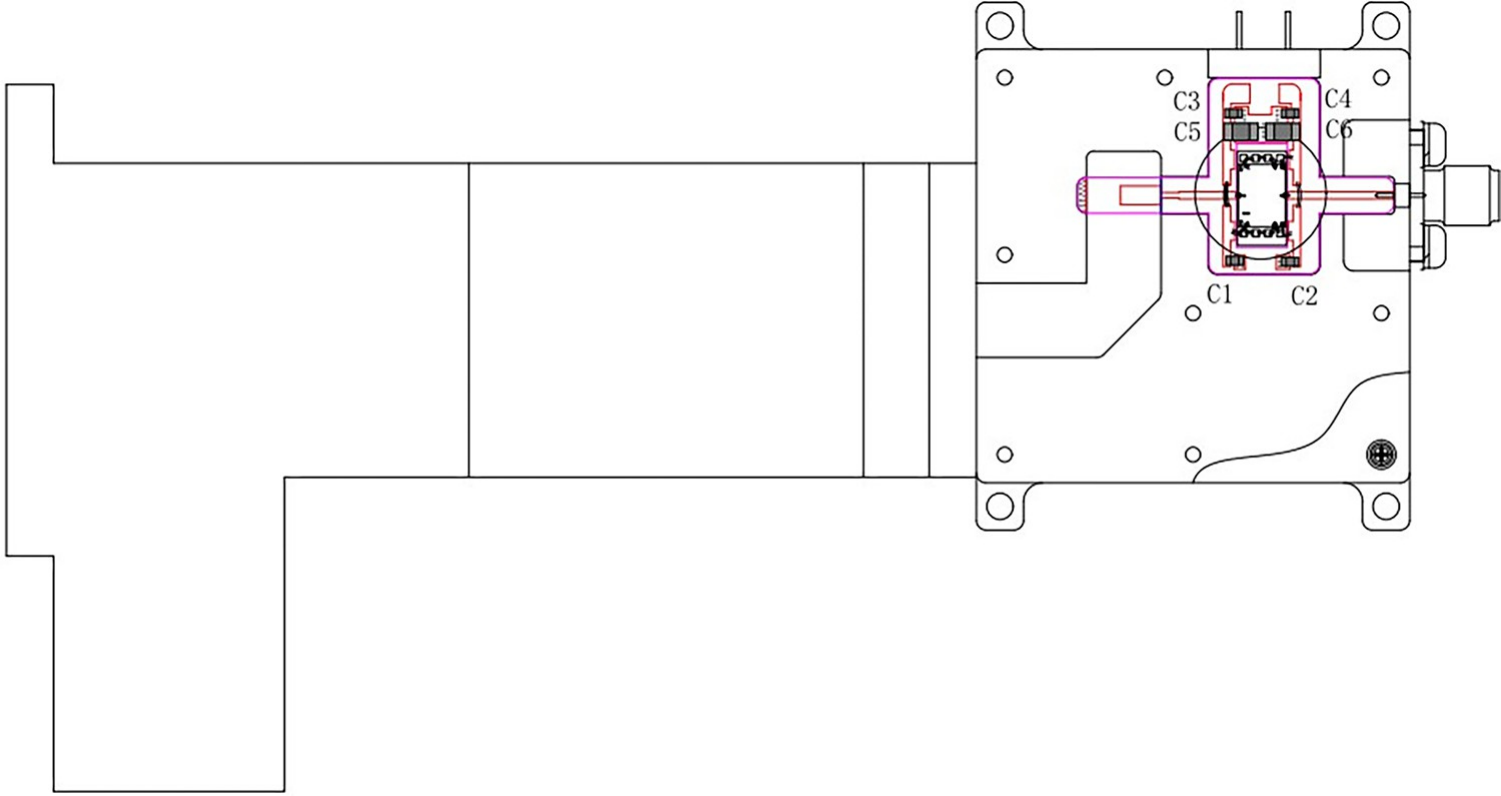
Schematic diagram of the final-stage amplifier module.

The microstrip-waveguide transition structure, within the module, transforms the output signal of the GaN chip into a standard waveguide port, in which harmonic components are subsequently suppressed by the isolation filter. Furthermore, real-time monitoring of the output-power amplitude is performed by a detector. The final-stage amplifier is saturated to enhance amplitude consistency between channels. The positive (40W) and reverse (20W) coupling port of the isolation filter facilitates one-way output, ultimately improving the output standing wave, preventing reverse feed-in, suppressing harmonic components, and ensuring that the P_1dB_ output power meets technical index requirements. See Fig 12 for details (Schematic, layout, and dimensional drawings are described in detail in S6, S13, S21, S28, S29 Figs in S1 Raw images).

**Fig 13:**
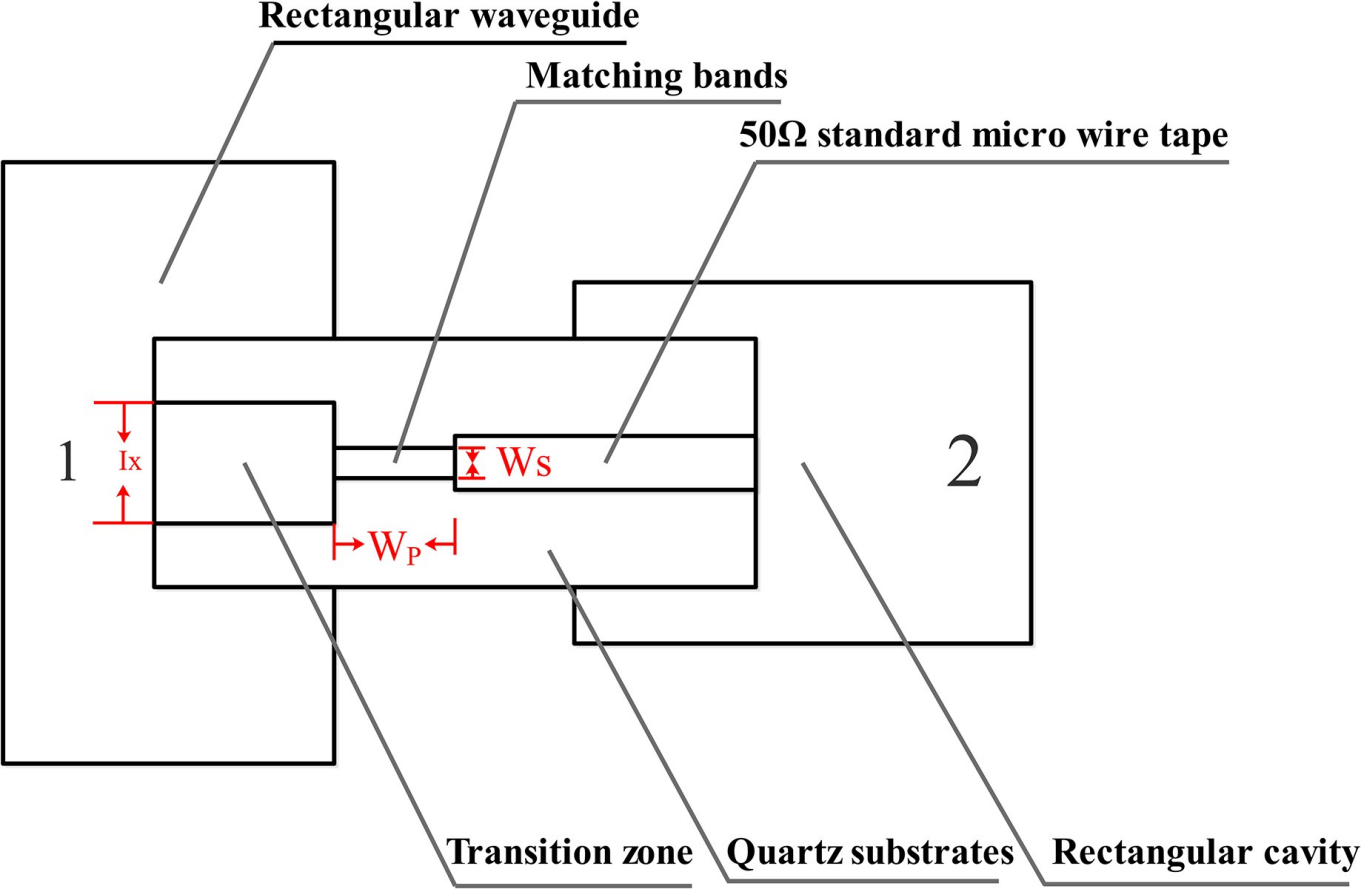
Schematic diagram of microstrip-translational waveguide transition.

The microstrip-waveguide transition design is crucial within this module, as its performance directly affects system output power, gain, and other parameters. Therefore, it is crucial for the transition design to exhibit good standing waves, high reliability, and relative insensitivity to assembly errors [66–69]. Fig 13 illustrates the relative position relationship between the microstrip and the waveguide output port (Detailed description in S32 Fig in S1 Raw images). The simple and compact transition of the module allows insertion into a section of the transition band. It serves as a coupling probe from the broad side center of the waveguide, facilitating the coupling of the TE_10_ mode in the waveguide to the microstrip. The distance between the transition band and the short pavement in the rectangular waveguide is approximately one quarter of a wavelength, positioning it at the strongest electrical field point of the waveguide. Stepped impedance lines are used to realize the transition band and the microstrip line between the matching networks. A quartz substrate passes through the rectangular waveguide, providing a waveguide window and aiding substrate positioning. This forms a sealed structure, enabling polarization of the rectangular waveguide in different directions.

**Fig 14:**
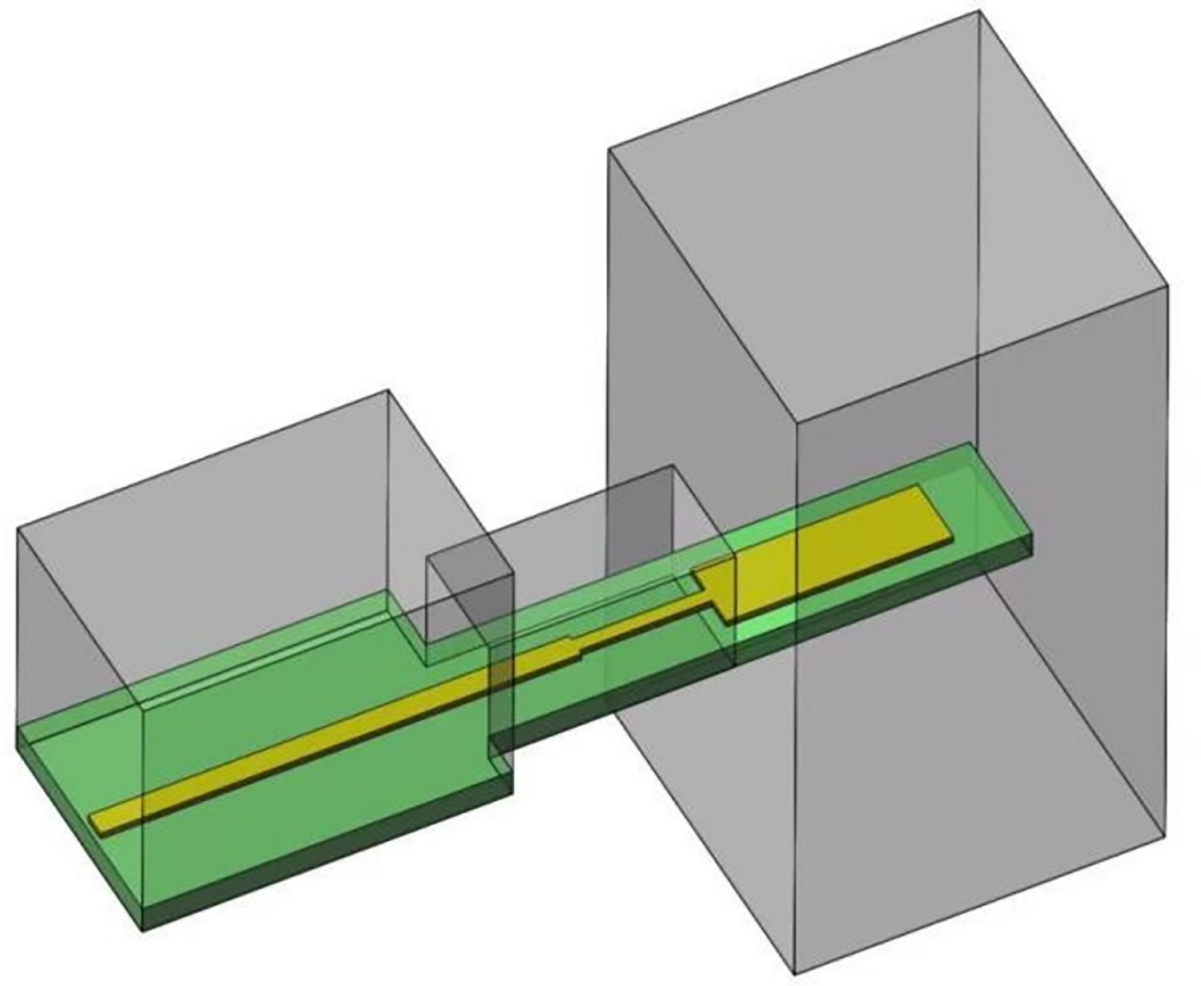
Simulation model of microstrip-to-waveguide transition.

As demonstrated in the transition diagram, the High Frequency Structure Simulator (HFSS) can model and optimize parameters with a significant impact on transition performance. The structural model is depicted in Fig 14.

**Fig 15:**
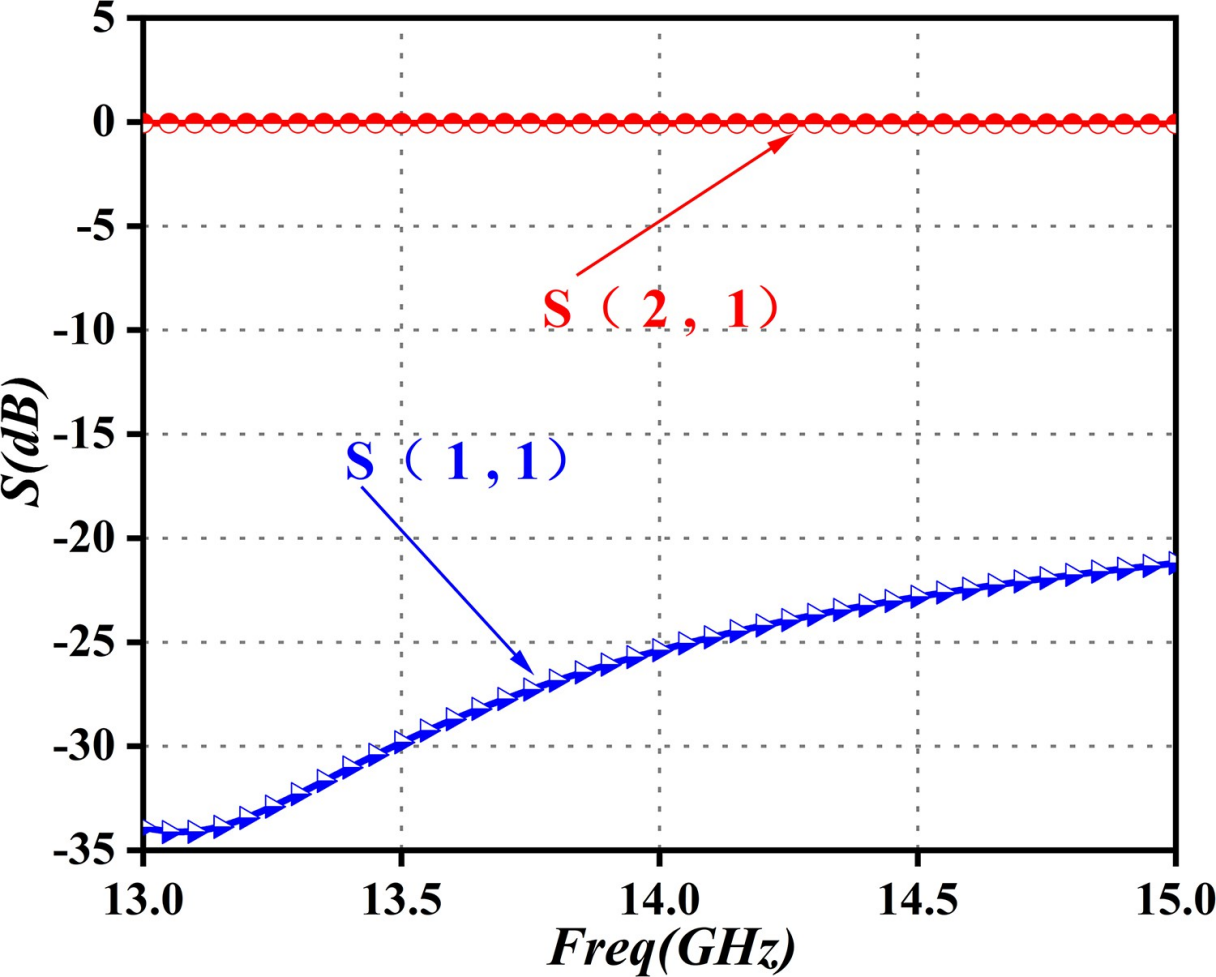
Simulation curve of microstrip-to-waveguide transition.

Fig 14 illustrates the microstrip-waveguide transition structure, which consists of a metal cavity housing a planar circuit. To ensure that the cavity exclusively accommodates the TE_10_-mode coupled planar circuit, optimal model values must be obtained through parameter scanning and optimization, and this primarily impacts transition performance. The key structural dimensions are as follows: transition-band length and width (d = 4.3mm, Ix = 2mm); matching-band length and width (Wp = 4.3mm, Wx = 2mm). Simulation results based on these dimensions are displayed in Fig 15.

**Fig 16:**
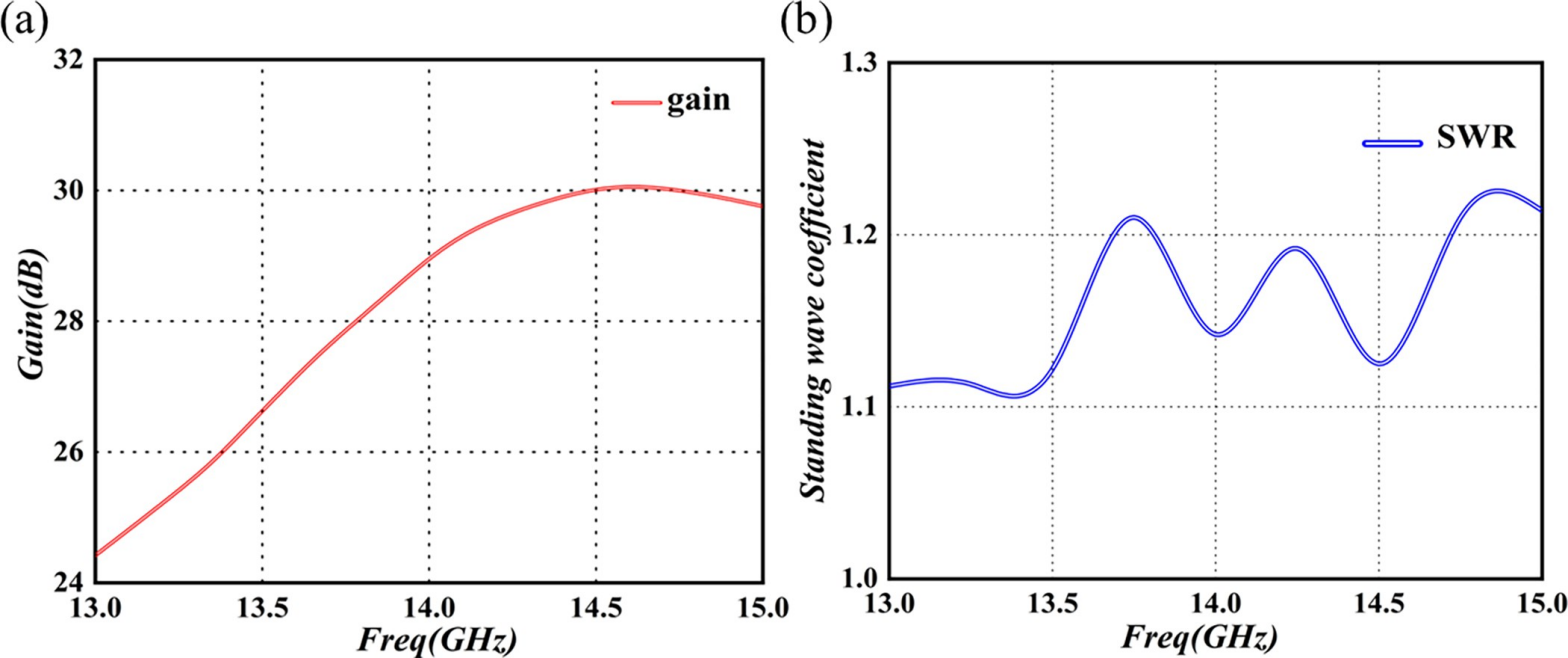
Performance test chart of the final-stage amplification module. (a) Frequency-gain test curve (b) Frequency-SWR test curve.

Fig 15 reveals a return loss better than -20dB within the 13.75GHz to 14.5GHz Ku-Band, with insertion loss below 0.1dB. This conversion structure exhibits a wide bandwidth, minimal insertion loss, and low return loss, making it an ideal choice that satisfies the necessary requirements. To further validate the microwave performance of the final-stage amplifier module, gain and standing-wave indicators were measured using a vector network analyzer, after system assembly. This procedure is illustrated in Fig 16.

In Fig 16, the gain of the final-stage amplifier module is seen to exceed 26dB within the frequency range of 13.75GHz to 14.5GHz, while the Standing Wave Ratio (SWR) is below 1.2. These results suggest that the module exhibits favorable linearity and SWR, meeting all the design requirements for the module.

### Overall design of the display and control unit

#### Power control module

**Fig 17:**
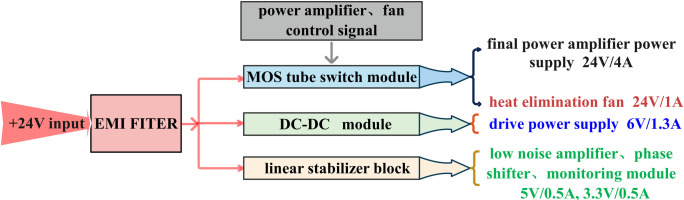
Power-supply unit: Schematic block diagram.

The power-supply unit incorporates a stable, integrated high-power DC-DC power-supply module, with a 24V DC input voltage. Initially, an Electro Magnetic Interference (EMI) power filter is employed to ensure the EMC test performance of the entire system. Subsequently, the process is divided into three branches: the MOS tube switches deliver an output of 24V voltage for amplifier and fan usage, the DC-DC module outputs 6.5V voltage for the drive-amplifier chip, and the linear regulator stabilizes 6.5V to produce 5V and 3.3V voltages for phase shift, attenuation, and usage monitoring. The schematic block diagram of the power-supply unit is illustrated in Fig 17 (Detailed description in S1-S9, S14-S16 Figs in S1 Raw images).

The PA module includes a pre-stage low-noise amplifier, and the total power consumption of the control chip is ≤ 1W. The drive PA power consumption is ≤ 3W/20% = 15W, and the final-stage PA power consumption is ≤ 90W. The fan power consumption is ≤ 15W, and the DC/DC total efficiency should be ≥ 90%. Therefore, the total power consumption of the Ku-Band PA is ≤ 140W.

#### Monitoring module

**Fig 18:**
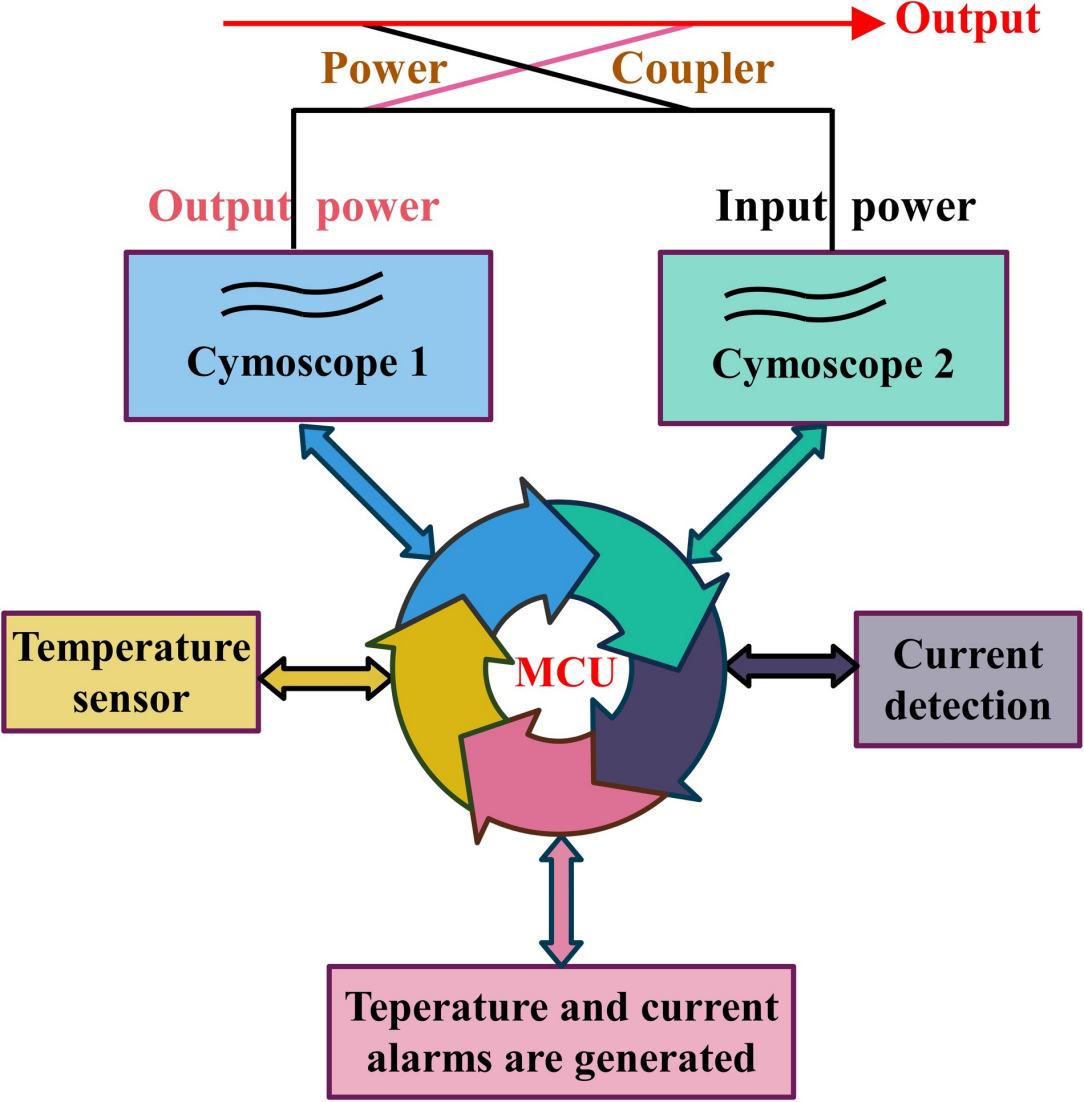
Monitoring-module control schematic.

The monitoring unit observes and manages the real-time working status of each module within the device. This unit facilitates remote monitoring, fault identification, and safety-protection functions. It also allows monitoring and control of the modular alarm functions (see Fig 18, Detailed description in S1-S9, S16-S18 Figs in S1 Raw images). The internal MCU is primarily responsible for monitoring power, phase shifting, attenuation, and temperature, and for executing other controls (such as fan on/off). Additionally, it conducts external controls through the 74HC595 serial and parallel port conversion, providing operational status parameters.

Fig 18 displays a schematic diagram, illustrating the three typical functions of the monitoring unit. Power detection is primarily accomplished through the transmitter output, using the coupler and detector. Simultaneously, the output power and reflected power are converted into analog voltage and transmitted to the MCU, where the latter assesses the output alarm signal. The current detection circuit is a well-established one. The overheating alarm depends on a built-in temperature-monitoring sensor chip, which queries the modular temperature every five seconds. Real-time monitoring of the system reflects the current operating status, ensuring the stability and reliability of the entire PA operation.

#### Complete PA design

**Fig 19:**
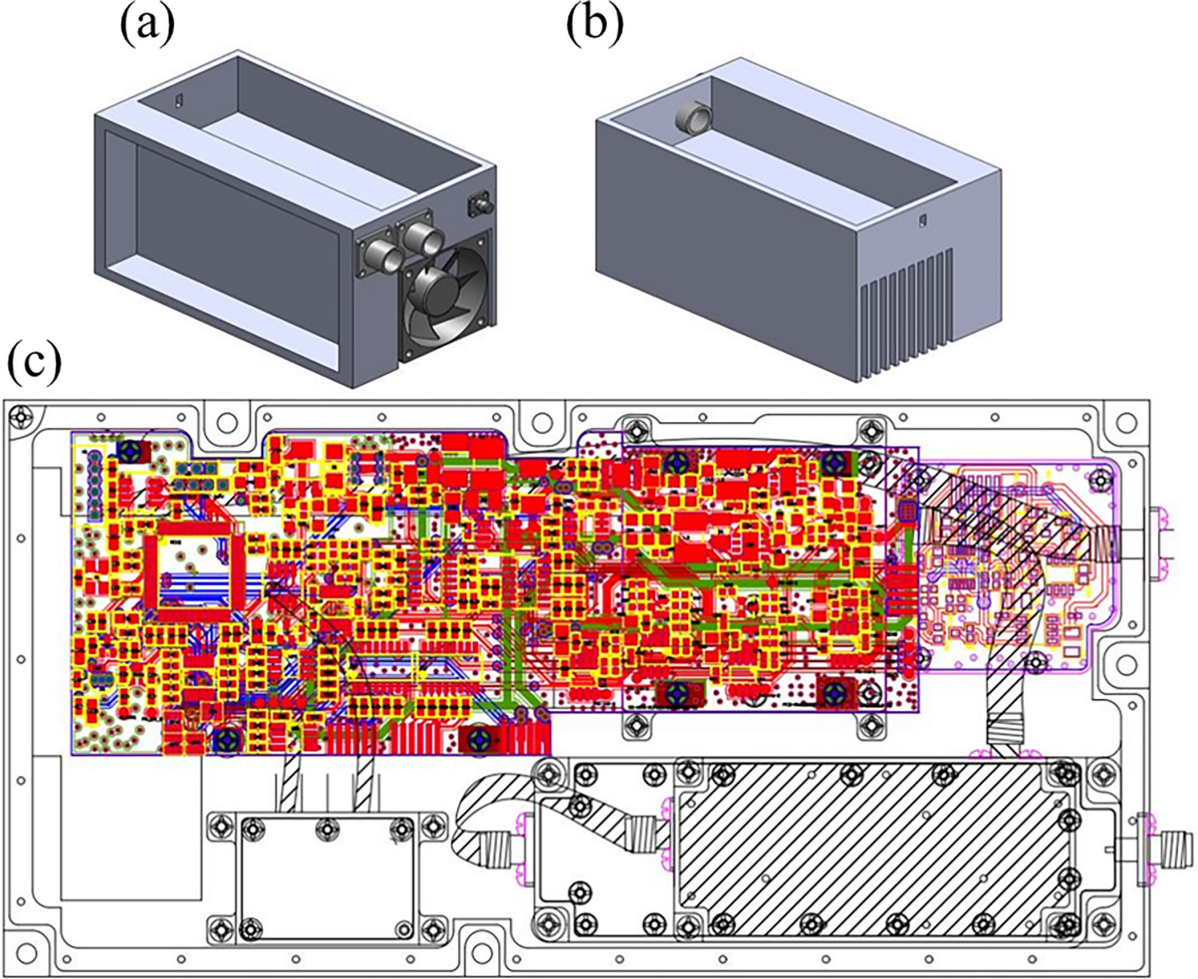
Diagram of the 20W RF transmit front-end PA for star Ku-Band. (a) Forced air-cooling design diagram (b) Grid-type heat-sink design diagram (c) PA internal layout diagram.

With the assistance of special experiments, including prototype production, environmental verification, and small-batch production, the performance index of the Ku-Band RF front-end PA for star has been configured to meet all design requirements. The system conducts module-level synthesis to facilitate amplifier reuse. Moreover, the rational design of the entire system ensures the integration of all modules (including RF, power supply, and monitoring) within a sealed milling cavity. Additionally, over-excitation protection, overheating protection, and other control and protection technologies are employed to enhance amplifier safety, reliability, and maintainability, and to achieve stable power output. Due to the large power consumption of the overall radar system, heat generation is a serious concern, and the working environment is more complex. In turn, this generates heat-dissipation difficulties, which can significantly reduce the life of this PA. Therefore, enhancement of the heat-dissipation efficiency of the PA is a crucial consideration in the design of this product. Effective heat-dissipation measures can be implemented to maintain the thermal balance of the PA. More specifically, to minimize the thermal resistance of the device, it is secured in the cavity with a maximum contact surface, using screw fastening or load welding, more efficiently to dissipate heat. Nevertheless, to optimize the overall thermal design, and to achieve efficient heat dissipation, a combination of forced air-cooling and grid-type heat-sink technology is utilized. This allows the thermal-performance parameter requirements to be satisfied. Moreover, this approach also permits the technical index of the entire PA to be realized, while also keeping cooling costs minimal, and the structure itself compact. The shape of the PA can be seen in Fig 19 (Detailed description in S1-S32 Figs in S1 Raw images).

## Experimental system

**Fig 20:**
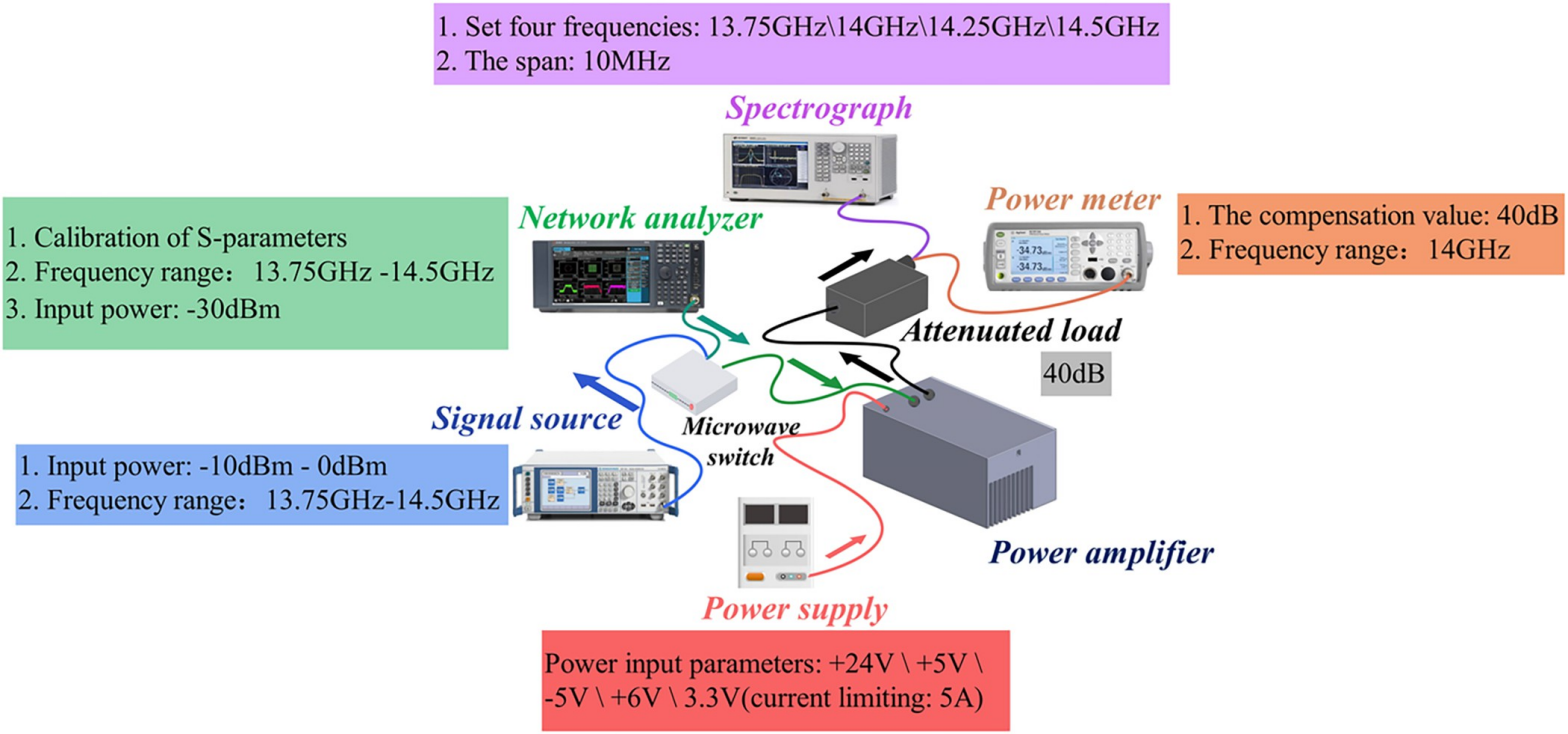
Experimental system test diagram.

The system test device has been developed according to the actual technical parameters and product-design specifications of the star. The output power, third-order cross-talking, power detection, spurious rejection, gain adjustment, harmonic rejection, phase-shift attenuation, and other parameters of the PA for the star were tested. The experimental system for these tests is depicted in Fig 20.

The assembly of the test system is illustrated in Fig 20. Each instrument undergoes independent calibration, after which the test parameters are established. In order to ensure the accuracy of the test, the instruments are powered on and preheated for more than thirty minutes. Next, the remote-control port is connected and the host computer is opened. The test can then begin.

First, one should connect the attenuation load to the waveguide output port of the amplifier. Connect the spectrum meter or power meter to the output coupling port of the test coupler. Connect the signal source or scalar network analyzer to the amplifier input port, using a cable or microwave switch. Second, power must be supplied to the amplifier. Thirdly and finally, connect the equipment to the host computer, thus enabling the sending of commands to control the operation of the equipment, and to initiate the testing of PA indicators.

## Measurement results and analysis

### P_1dB_ output power test

**Fig 21:**
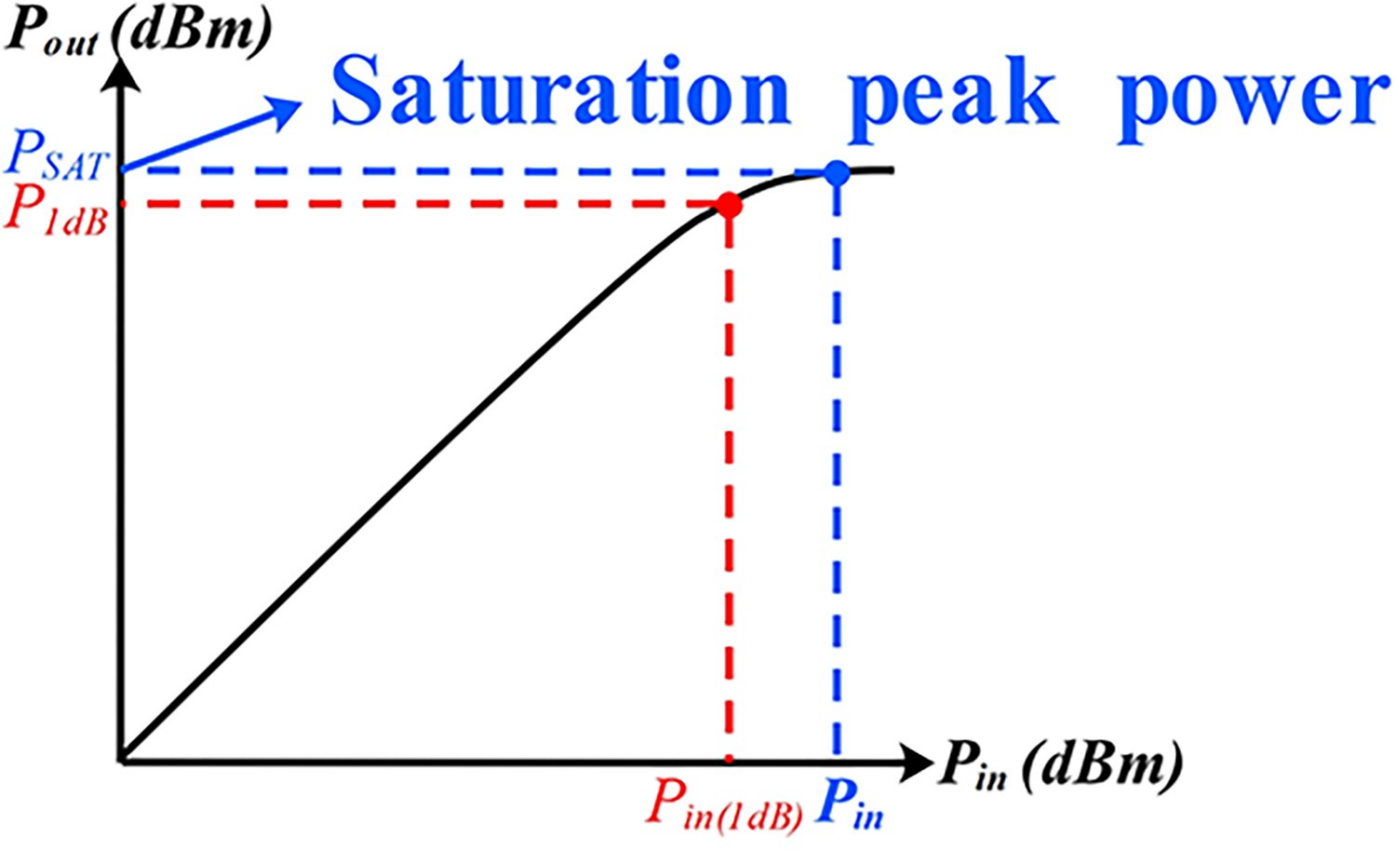
PA input-output relationship diagram.

The power gain of the amplifier is commonly used to assess its linearity in terms of the power point. Linearity is considered when the linear gain is less than 1dB, indicating that the relationship between input and output no longer exhibits linear growth, as input power increases. This results in the phenomenon known as gain compression, and specifically, the 1dB compression point [70]. See Fig 21 for an illustration.

**Fig 22:**
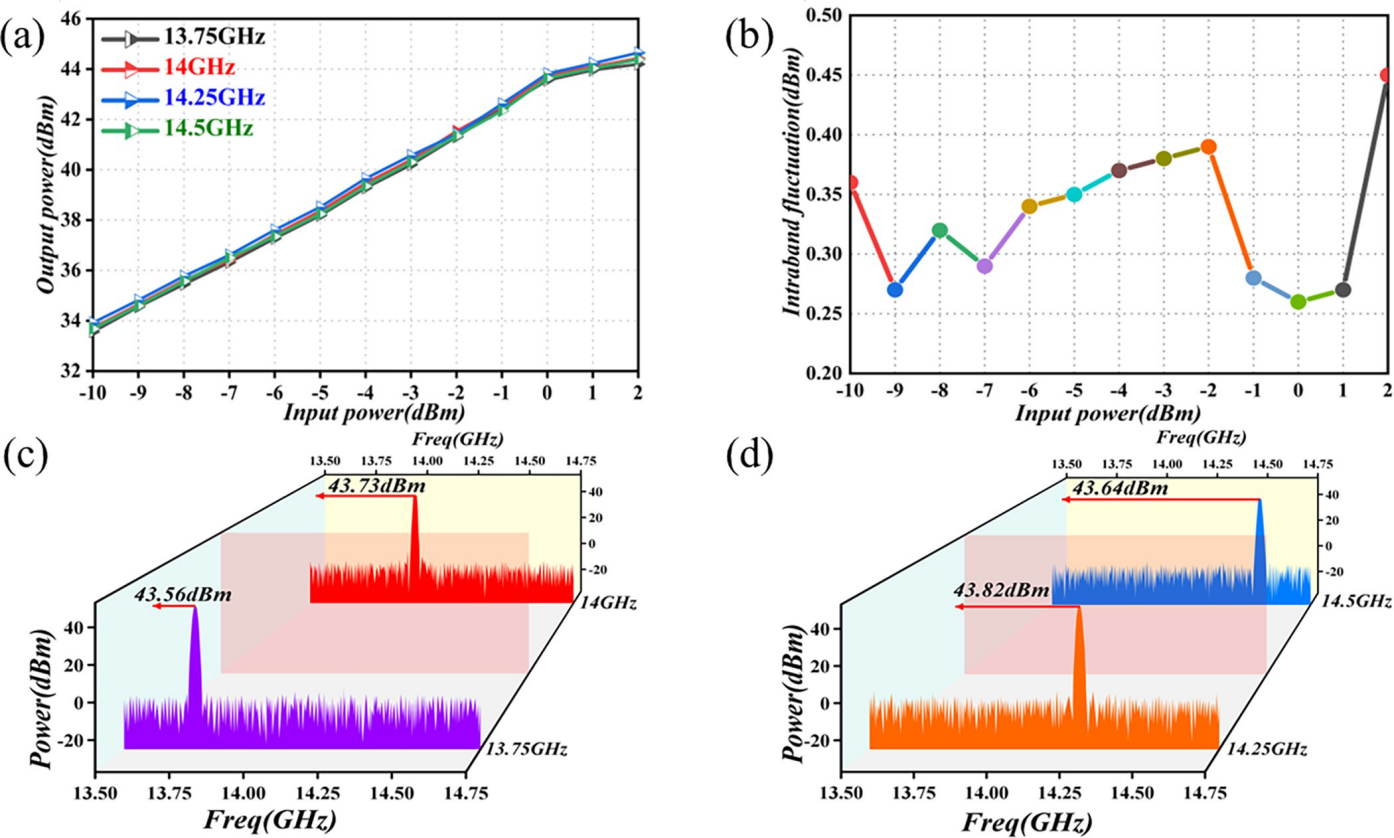
Output-power test-results graph. (a) Power-output test chart (b) Output-power flatness in frequency band (c) P_1dB_ output power at 13.75GHz and 14GHz frequency points (d) P_1dB_ output power at 14.25GHz and 14.5GHz frequency points.

In the practical PA design, a higher P_1dB_ value signifies better linearity. Thus, the output power of the radar PA was assessed through a single controlled-variable method, within the operational frequency range of 13.75GHz to 14.5GHz. The results were recorded using a power meter and a spectrometer, as illustrated in Fig 22.

Fig 22(A) depicts the variation curve of the output power for the entire PA module, in relation to input power. It is evident from the figure that the output power in the linear operating state, within the 13.75GHz to 14.5GHz band, increases with the input power. Additionally, when the input is 0 dBm, the output power corresponds to P_1dB_. With a continuous increase in input, the PA module enters a saturation state, reaching peak power values of 44.2dBm, 44.43dBm, 44.65dBm, and 44.38 dBm. Fig 22(B) illustrates the fact that the in-band output-power flatness of the PA at different input powers is < 0.5dBm. Fig 22(C) indicates that, at 0dBm input, the P_1dB_ output power at the frequency points of 13.75GHz and 14GHz is 43.56dBm and 43.73dBm, respectively. Fig 22(D) shows us that at 0dBm input, the P_1dB_ output power at the 14.25GHz and 14.5GHz frequency points is 43.82dBm and 43.64dBm, respectively. Experimental results confirm that, in the 13.75GHz to 14.5GHz frequency band, the PA output power is > 43dB, and its in-band flatness is < 0.5dBm. This demonstrates good linearity and compliance with the technical-index requirements for the designed star radar output power.

**Fig 23:**
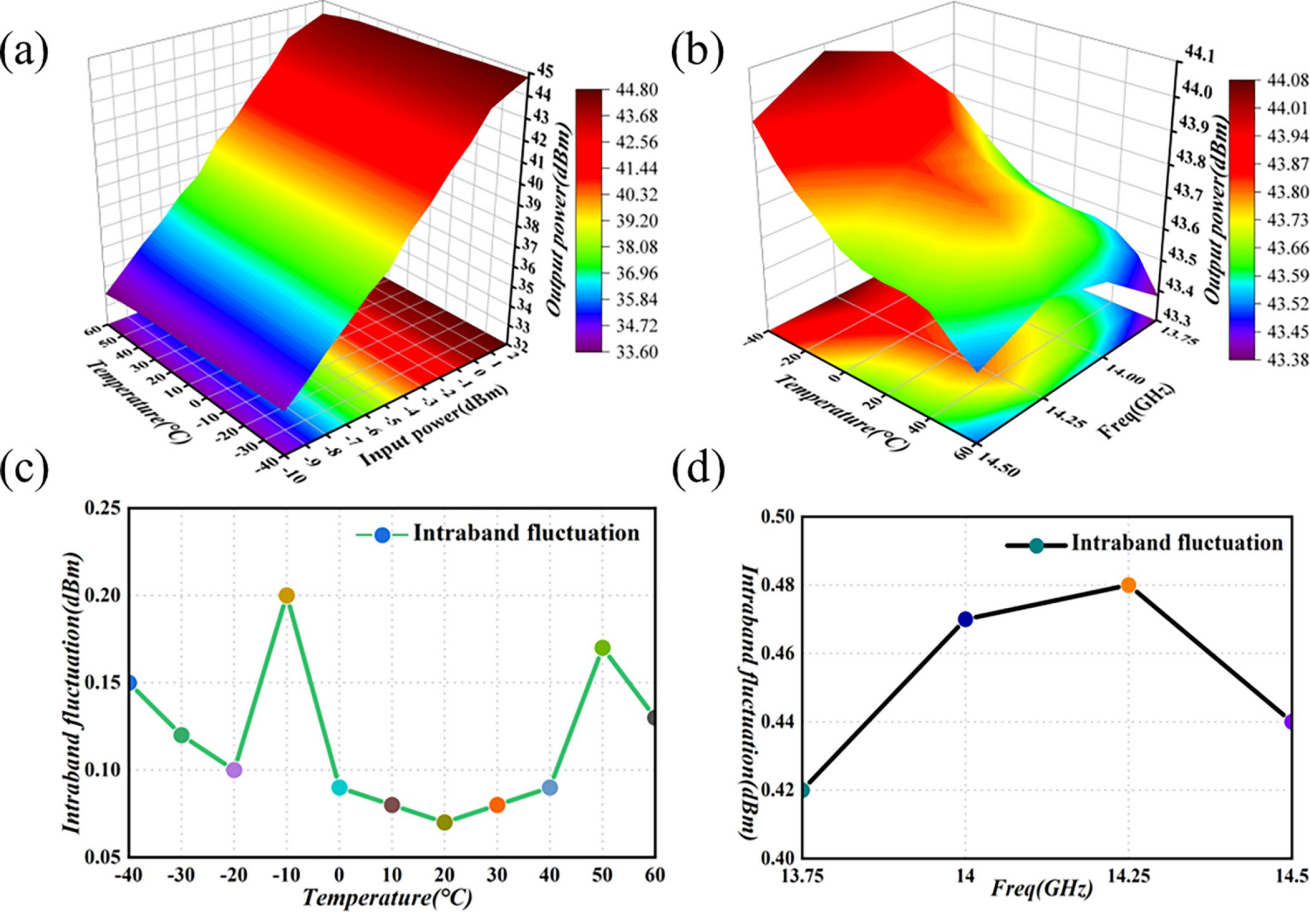
Graph of environmental test results. (a) Input–temperature–output power (b) Frequency–temperature–output power (c) Output-power flatness at the limit temperature (d) Output-power flatness in the full frequency band.

To enhance the reliability assessment of the Ku-Band PA for satellite applications, we conducted environmental cycling experiments, and specifically, limit experiments. These tests involved exposing the amplifier to high temperatures (+60°C) and low temperatures (-40°C). The chosen temperature range significantly exceeded the actual working temperature range of the product in satellite environments. The findings from these experiments are graphically depicted in Fig 23.

The reliability test process involves operating the amplifier in a saturated output state. Fig 23(A) illustrates the fact that, at a frequency of 14GHz, the output power varies continuously with the temperature, within the input-power range. Moreover, Fig 23(B) demonstrates that, in the 13.75GHz to 14.5GHz band, the P_1dB_ output power increases continuously with decreasing temperature. Meanwhile, Fig 23(C) indicates that the output-power flatness in the extreme temperature range is < 0.3dBm, and Fig 23(D) shows that the output-power flatness in the 13.75GHz to 14.5GHz band is < 0.5dBm. The results of the environmental test at extreme temperatures indicate that, within this range, the PA is influenced by the ambient temperature, albeit to a minimal extent. The test results are more pronounced. The output power of the PA across the entire frequency and temperature range complies with the design specifications for satellite radar.

### Third-order intermodulation (IMD3)

**Fig 24:**
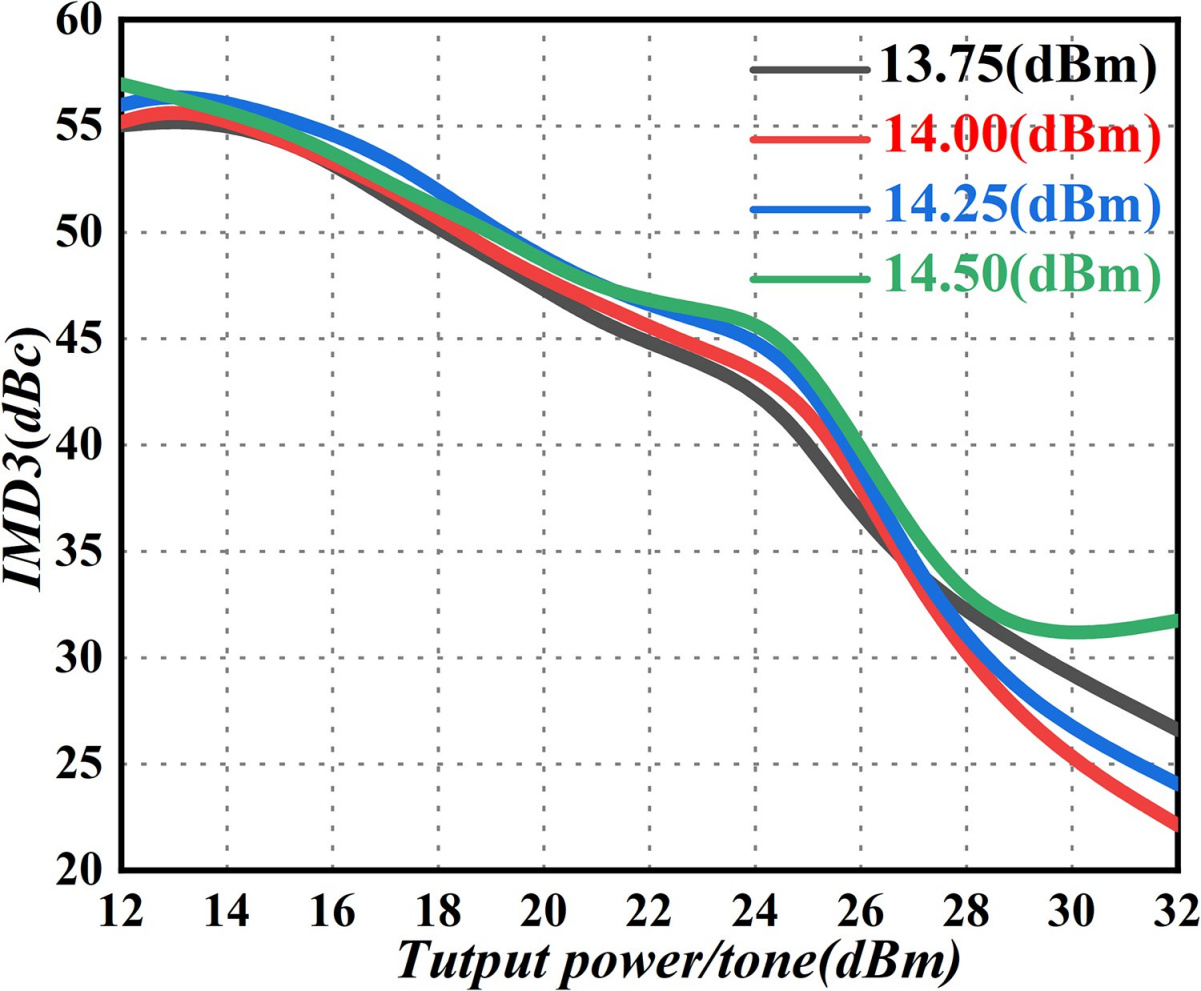
Third-order cross-tuning simulation curve of the final-stage amplifier module.

The nonlinear operating state of the PA induces intermodulation distortion, predominantly in harmonics and cross-tuning [71]. Given that harmonic distortion and the output signal occur at different frequency points, the second harmonic of the Ku-Band amplifier, at P_1dB_ output, is approximately -25dBc. This can be reduced to below -65dBc by incorporating a waveguide harmonic-suppression filter at the amplifier output port, resulting in a suppression system exceeding 40dBc at the harmonic position. Consequently, the associated impact can be disregarded. Incidentally, the most notable impact is on output-signal performance. The main distortion component in the intermodulation distortion is, in fact, third-order intermodulation distortion. Since the driver-amplifier module has an ample power margin and operates linearly, the third-order intermodulation of the PA is primarily determined by the final amplifier module. Consequently, the third-order intermodulation index curve of the final amplifier module is derived through simulation. This process is illustrated in Fig 24.

**Fig 25:**
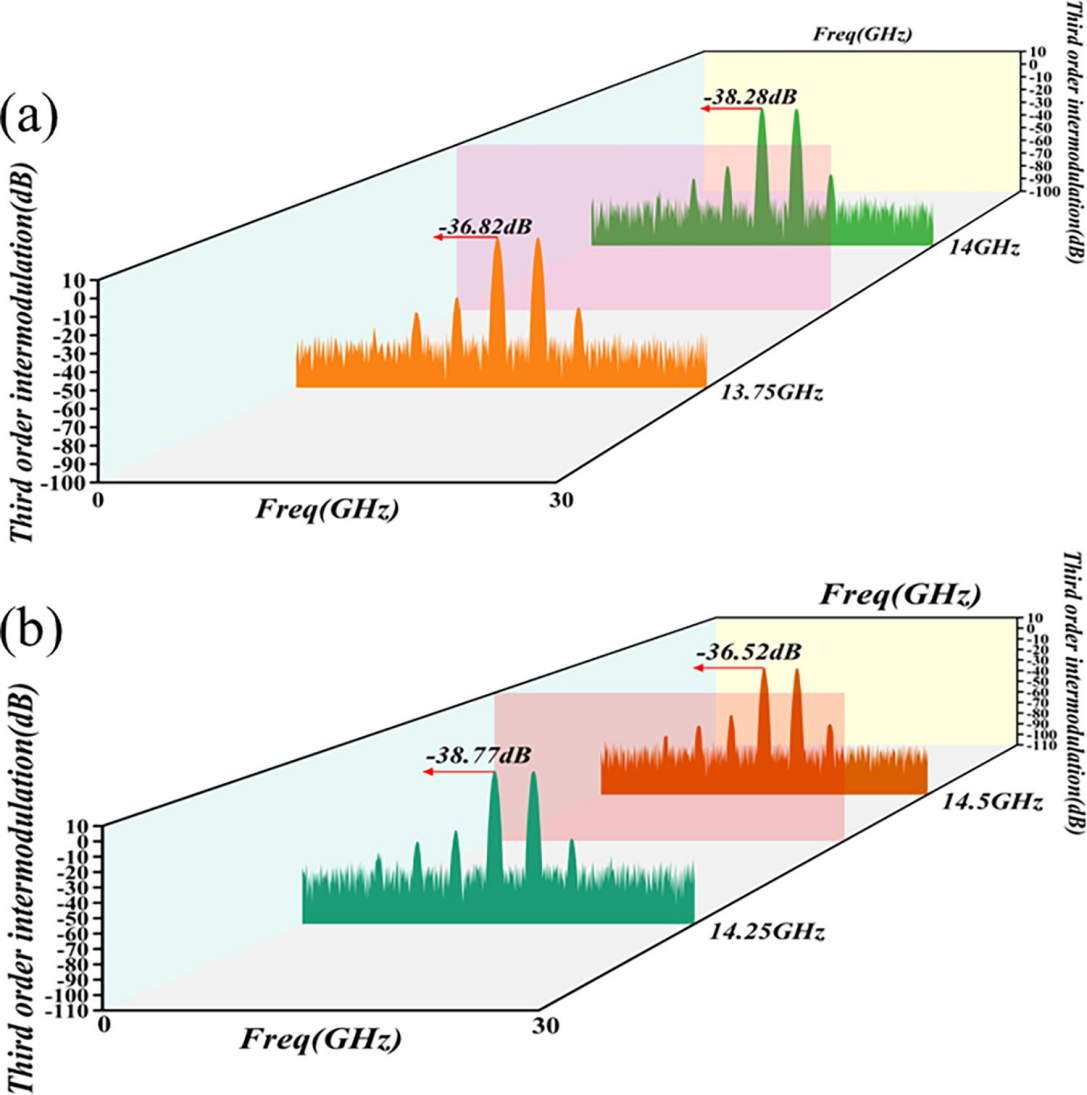
Third-order intermodulation test results. (a) Third-order intermodulation at 13.75GHz, 14 GHz (b) Third-order intermodulation at 14.25GHz, 14.5GHz.

According to the simulation curve of the final-stage amplifier chip, at a total power of a single tone, decreasing from 46dBm to 7dBm (equivalent to the total power of a dual tone decreasing from 46dBm to 4dBm, or the combined output of 42dBm), the single-chip output power is approximately 28.5dBm. Simultaneously, the full frequency range of third-order intermodulation in the final-stage amplifier is ≤ -23dBc, meeting the design requirements for third-order intermodulation in the final-stage amplifier. To further validate the third-order intermodulation parameters of the entire system, the indicator was tested, as illustrated in Fig 25.

Fig 25 reveals that the third-order cross-tuning of the PA, at frequency points 13.75GHz, 14GHz, 14.25GHz, and 14.5GHz, is -36.82dBc, -38.28dBc, -38.77dBc, and -36.52dBc, respectively, surpassing the 23dBc threshold. This finding strongly confirms that the output signal is minimally affected by the nonlinear effects of the amplifier, indicating excellent linearity and adherence to the requirements of satellite radar applications. In summary, the intermodulation component introduces spectrum-expansion issues in the amplifier, leading to spectrum waste. To mitigate this problem, IMD3 should be minimized (typically below -40dBc), while truncation power should be judiciously increased. Subsequent strategies, such as the feed-forward method, back-off method, and other auxiliary approaches, can be implemented to ensure and enhance the overall linearity of the amplifier.

### Power detection

**Fig 26:**
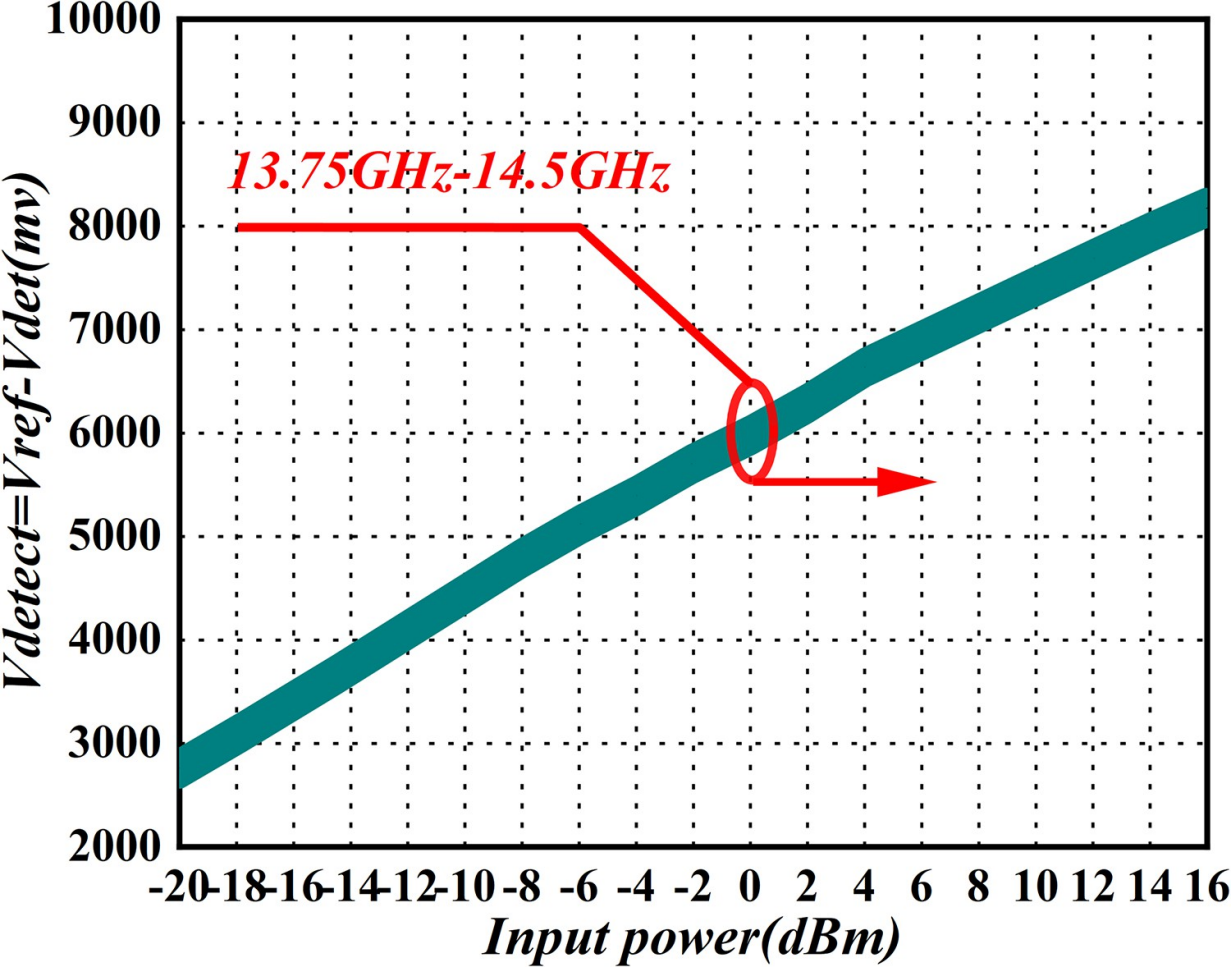
Power-detector simulation diagram.

The detector module performs real-time monitoring of the output power, concurrently measuring both forward transmission power and reflected power. Additionally, it incorporates various protection functions contingent on power levels. The power-detection performance of the detector module is illustrated in Fig 26.

In Fig 26, within the 13.75GHz to 14.5GHz band, the forward coupler exhibits a coupling degree of approximately -38dB, effectively amplifying the output power within the actual range of 18 dBm to 54dBm. Specifically, at an amplifier output power of 46dBm, showcasing a 30dB large dynamic range, precise power detection is conducted in order to couple the RF signal. The signal is extracted at 9dBm, and subsequently, the voltage from the forward output detector of the unit real-time monitoring amplifier is utilized to govern the gain attenuator. This achieves a closed-loop adjustment function for output power. This process ensures sustained accuracy in output power, maintaining the latter within ±0.5dB, in adherence to the specified technical indicators.

### Spurious and harmonic suppression

#### Spurious suppression

The spurious signal consists primarily of a frequency-conversion component and a power-supply component. This will reduce the signal-to-noise ratio of the output signal, and the range accuracy and distance resolution of the radar. Thus, in the present paper, a systematic approach is implemented to suppress spurious responses [72]. First, the spectrum combination indicates that the spurious level of the nearest part outside the band is 16.5GHz. This occurs when the local oscillation frequency is 11.5GHz, and the spurious frequency point falls outside the range of 13.75GHz to 14.5GHz. Nonetheless, there remains a 2GHz frequency difference from the highest frequency of 14.5GHz in the passband. This signifies that there is no intermodulation spurious level in the band, and the microstrip filter in the line suppresses more than 40dB. Combined with the third-order component power of about 25dB, a suppression system of more than 65dB is sufficient to facilitate spurious suppression.

**Fig 27:**
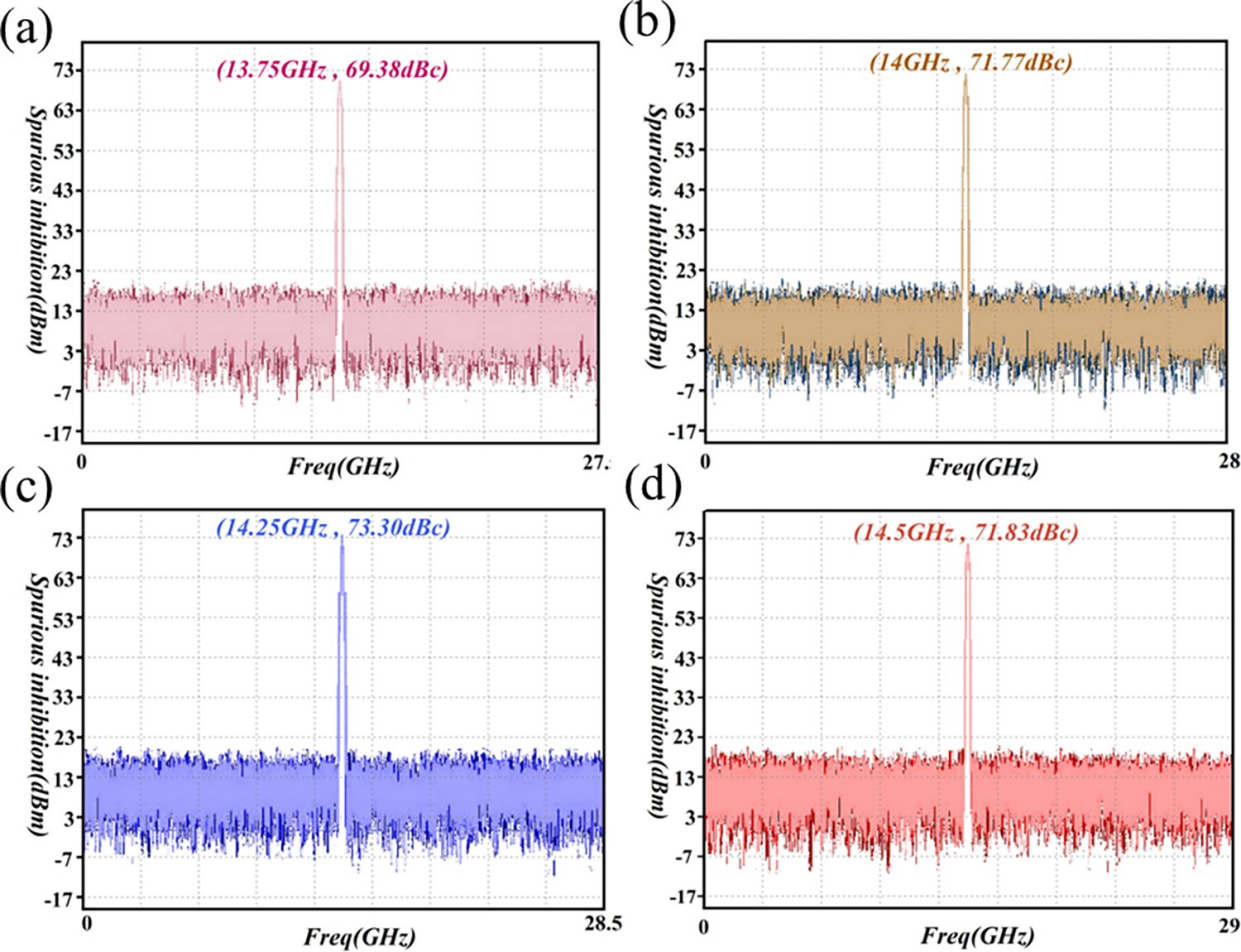
Spurious suppression test-result graph. (a) 13.5GHz spurious suppression (b) 14GHz spurious suppression (c) 14.25GHz spurious suppression (d) 14.5GHz spurious suppression.

Second, the frequency leakage of the local oscillation is also an important source of spurious signaling; the local oscillation signal size of this program is 13dBm. Thus, leakage of the local oscillation signal size to the RF channel is approximately -27dBm, corresponding to a level of about -70dBm after 43dB suppression by the microstrip filter. Line-gain distribution can be seen in the linear region of operation when its input power is -5dBm. At this time, the suppression system evinces 65dB, to meet the requirements of spurious suppression. Additionally, the secondary power supply has clear requirements for ripple indicators, while the amplifier module itself is designed with a filtering circuit, in order to generate optimal transmission characteristics. In real-life engineering applications, spurious suppression can be ensured above 65dB. In turn, this fulfils the requirements of the index. The spurious suppression test is presented in Fig 27.

The results depicted in Fig 27 reveal spurious rejection coefficients of 69.38dBc, 71.77dBc, 73.3dBc, and 71.83dBc, at the frequency bands of 13.5GHz, 14GHz, 14.25GHz, and 14.5GHz, respectively. This fulfills the stipulated requirement of harmonic rejection exceeding 65dB for the star PA. These findings provide additional validation for the efficacy of the proposed rejection theory in enhancing spurious rejection.

### Harmonic suppression

**Fig 28:**
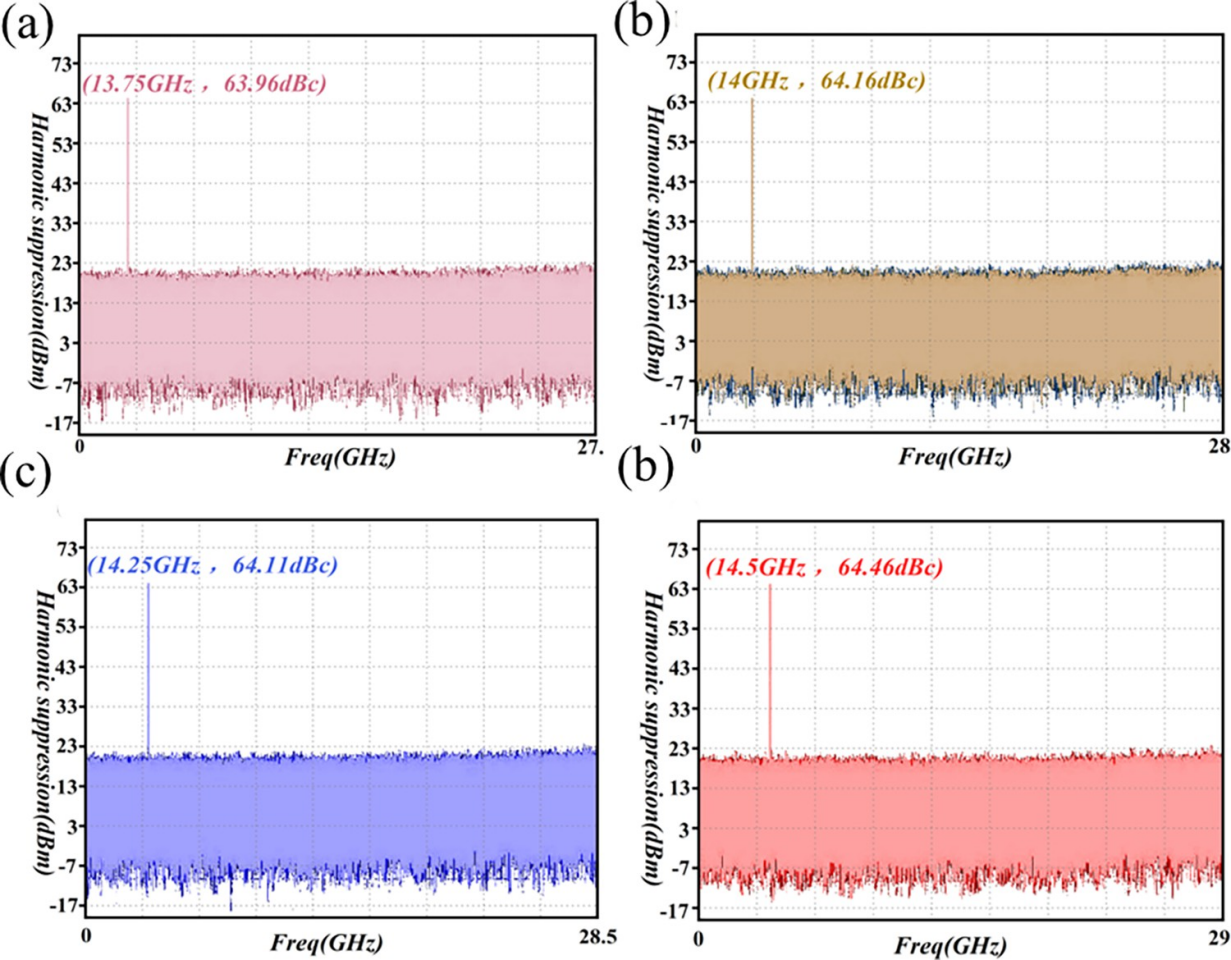
Harmonic suppression test-result graph. (a) 13.5GHz harmonic suppression (b) 14GHz harmonic suppression (c) 14.25GHz harmonic suppression (d) 14.5GHz harmonic suppression.

The harmonic-rejection characteristics of the amplifier are highly significant, and the potential harmonic distortion can be assessed through the second harmonic-distortion input signal. This distortion can lead to increased compression phenomena, and interference with other frequency bands. Consequently, the design process must carefully consider harmonic rejection [73, 74]. This paper employs the load-traction suppression-design approach to optimize the performance of the active device, complemented by the addition of a waveguide harmonic-suppression filter at the output of the amplifier. For the Ku-Band amplifier, with a second harmonic P_1dB_ output of approximately -25dBc, effective second harmonic suppression is imperative, requiring levels below -60dBc for practical engineering applications. The feasibility of the design was further scrutinized through relevant experimental tests, as depicted in Fig 28.

The test results presented in Fig 28 reveal significant harmonic rejection coefficients at 13.5GHz, 14GHz, 14.25GHz, and 14.5GHz bands, measuring -63.95dBc, 64.16dBc, -64.11dBc, and -64.46dBc, respectively. Consequently, the harmonic rejection across the entire frequency spectrum surpasses 60dBc, aligning with the specified requirements for Ku-Band PAs in star applications. This information underscores the consistent harmonic-rejection performance of the amplifier throughout the frequency band, exceeding the 60dBc threshold, and satisfying the technical index standards for Ku-Band PAs.

### Gain adjustment stability

**Fig 29:**
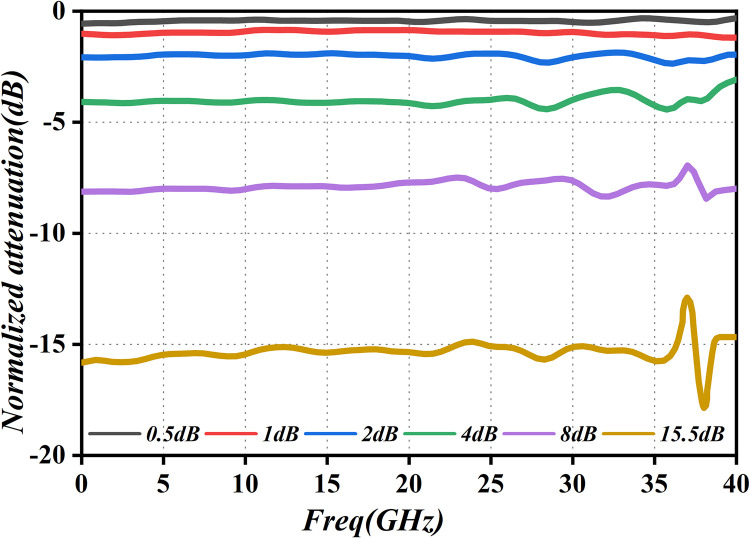
Attenuation simulation graph.

In this investigation, gain adjustment is attained through the modification of the attenuation level in the attenuator. Utilizing two CNC attenuators in a cascading design, we fulfill the criteria for a 30dB dynamic range, ensuring control over gain and amplitude indices. Additionally, this configuration exhibits improved in-band flatness characteristics. Simulation results, presented in Fig 29, illustrate the index curves corresponding to attenuation levels of 0.5dB, 1dB, 2dB, 4dB, 8 dB, and 16dB. These curves offer valuable insights into the attenuation process, providing a comprehensive understanding of the performance of the system under various attenuation settings.

**Fig 30:**
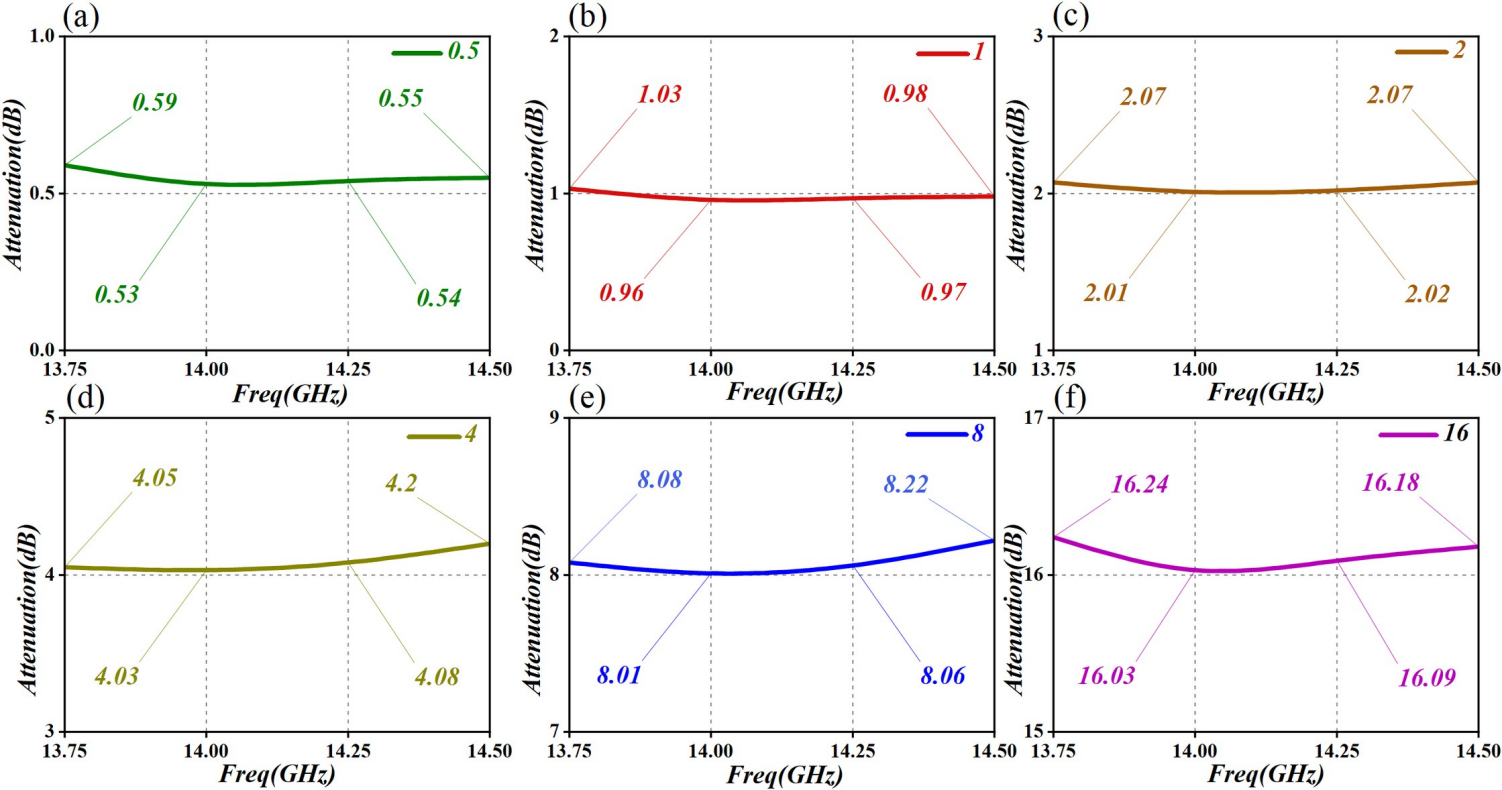
Attenuation performance test-results graph. (a)Attenuation of 0.5dB (b)Attenuation of 1dB (c)Attenuation of 2dB (d)Attenuation of 4dB (e)Attenuation of 8dB (f)Attenuation of 16dB.

Fig 29 indicates that the attenuation errors within the 0GHz to 40GHz range are minimal, meeting all corresponding attenuation requirements. Concurrently, to ensure the stability of this stellar PA in real-world applications, its attenuation performance has undergone rigorous testing, with the results depicted in Fig 30, which showcases the measured attenuation values.

Fig 30 reveals that the attenuation levels at 13.5GHz, 14GHz, 14.25GHz, and 14.5GHz align well with the simulation outcomes, displaying an attenuation error of ≤ 1%. To enhance attenuation performance, the implementation of a π-type attenuation network for impedance matching is recommended, in subsequent debugging phases. This approach ensures the attainment of optimal noise characteristics and attenuation responses.

**Fig 31:**
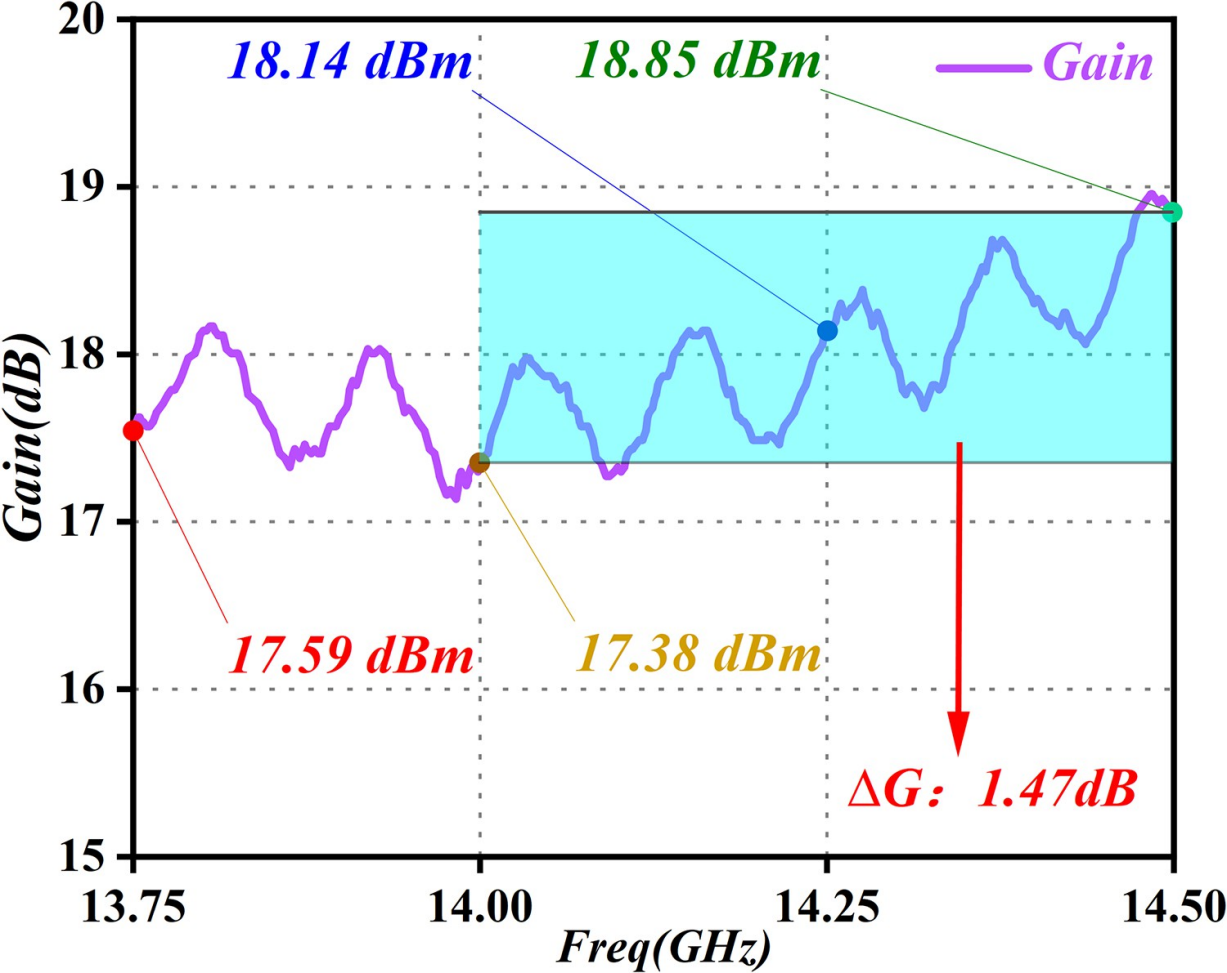
Gain-test curve graph.

For further assessment of gain stability within the amplifier frequency band, the power gain provides insight (see Fig 31). The amplitude difference between the highest and lowest gains in the band, denoted as ΔG (dB), serves as a crucial metric for evaluating the overall stability of the gain of the PA throughout its operational spectrum.

It is evident from Fig 31 that the gains at 13.5GHz, 14GHz, 14.25GHz, and 14.5GHz bands are 17.59dB, 17.38dB, 18.14dB, and 18.85dB, respectively, while the gain flatness in the band from 13.5GHz to 14.5GHz is 1.47dB. As this is below 2dB, it meets the gain-flatness requirements of the system.

## Discussion

Table 3 illustrated the results of our comparative analysis of various Ku-band PAs, set against our current study. This PA has been meticulously designed, taking into account the specific demands of satellite communication, ensuring exceptional performance and relative superiority. Through adept design and innovative technical approaches, the amplifier has effectively reduced noise, ensuring clear, high-quality signal transmission for satellite communication. The robust 20W output power represents a significant breakthrough in signal-transmission capability, establishing its leadership in the Ku-band PA domain. A holistic consideration of critical technologies, including output-power flatness, spurious suppression, and harmonic suppression, positions this PA as proficient in intricate communication environments, guaranteeing signal stability and purity. The adaptability of the amplifier is further enhanced by flexible gain and phase-adjustment features, catering to diverse communication scenarios and requirements, thereby fortifying the applicability and robustness of the entire satellite communication system. Especially noteworthy is the transition of the amplifier from theoretical design to successful deployment on satellites, with reliable operation. Validation through practical satellite applications lends substantial support to its advancement, infusing fresh energy into the ongoing evolution of satellite-communication technology, and steering the trajectory of future system development.

**Table 3:** Performance comparison of Ku-band PAs.

References\ Specs	[75]	[76]	[77]	[78]	[79]	[80]	[81]	This work
**Freq (GHz)**	12–18	14.7–15	5–33	12–20	17.7–18.3	13.5–18	2.3–21	13.75–14.5
**NF (dB)**	2.5	2.5	13.3	1.51	-	-	4.56	1.2
**Output P**_**1dB**_ **power (dBm)**	19	19.2	14	5	28.8	≥ 40	10.5	≥ 43
**Output power flatness(dBm)**	1	0.6	≥ 3 dBm	2.8	≤ 0.5	≤ 0.5	-	≤ 0.5 dBm (Full temperature section)
**Stray suppression**	-	-	-	-	-	-	-	≤ -65 dBc
**Harmonic suppression**	-	-	-	-	-	-	-	≤ -60 dBc
**Gain adjustment**	-	1.5–17.3dB	24–27 dB, steps of 10 dB	20.1–28 dB	0–21.4 dB, the gain difference across 2:1 SWR phase variations is only 0.8 dB	30–32 dB	an average gain of 13.5 dB with ripples of 0.2 dB, an excellent gain in the flatness of 13.5 ± 0.2 dB	0 dB-30 dB, steps of 0.5 dB, gain flatness ≤ ± 2 dB, attenuation error ≤ 1%
**Phase shift regulation**	-	0°-360°, steps of 22.5°, Phase shift accuracy of 0.7–0.8 dB	0°-360°, steps of 4.7°, Phase shift accuracy of 1 dB	the phase imbalance is 87.8° between ports 2, 3 and 2, 5 and 0.18° between ports 2, 4	-	-	-	0°-360°, steps of 5.625°, Phase shift accuracy of 0.5 dB

- Indicates that the study did not mention the indicator

## Conclusion

This paper introduces a novel Ku-Band 20W RF transmitting front-end PA, tailored to address issues prevalent in satellite communication systems, such as poor stability, low linearity, high cost, low efficiency, and large size. The paper commences with an overview of the RF modules, followed by a comprehensive theoretical analysis and simulation to establish an optimized design methodology for the key parameters of the system. The low noise amplification design of the PA is then theoretically calculated and optimized, accompanied by a detailed analysis, index allocation, and simulation design for the system. Subsequently, the paper concludes with the completion of the system design, prototype production of the PA, and experimental testing to ascertain the feasibility of the proposed design scheme. Notably, the space simulation-environment test results reveal that the Star Ku-Band PA surpasses 20W in output power, reaching a peak power of 26W, with power fluctuation ≤ ±0.5dBm, third-order intermodulation ≤ -23dBc, spurious rejection ≤ -65dBc, harmonic rejection ≤ -60dBc, power-detection range of +18dBm ~ +54dBm, gain-adjustment range of 0dB - 30dB, phase-shift adjustment range of 0°- 360°, and gain flatness < 1dB. These specifications align with the actual application requirements of the starboard technical indicators. This paper presents the development of a 20W RF transmitter front-end PA in Ku-Band for satellites that, in contrast to existing PA module specifications, offers advantages such as stability, high linearity, low cost, and small modularity, while maintaining a stable power output. This positions the proposed system as a frontrunner in the market. The successful deployment of the product in orbit has yielded positive socio-economic benefits, playing a crucial role in advancing the localization process of China’s satellite-based PA technology.

## Supporting information

S1 Raw images (PDF)
