# Supplementary material for: Catalyzing satellite communication: A 20W Ku-Band RF front-end power amplifier design and deployment
Source: PLoS One. 2024 Apr 10;19(4):e0300616. doi: 10.1371/journal.pone.0300616 (PMC11006151; doi:10.1371/journal.pone.0300616)

Fig S1. MCU

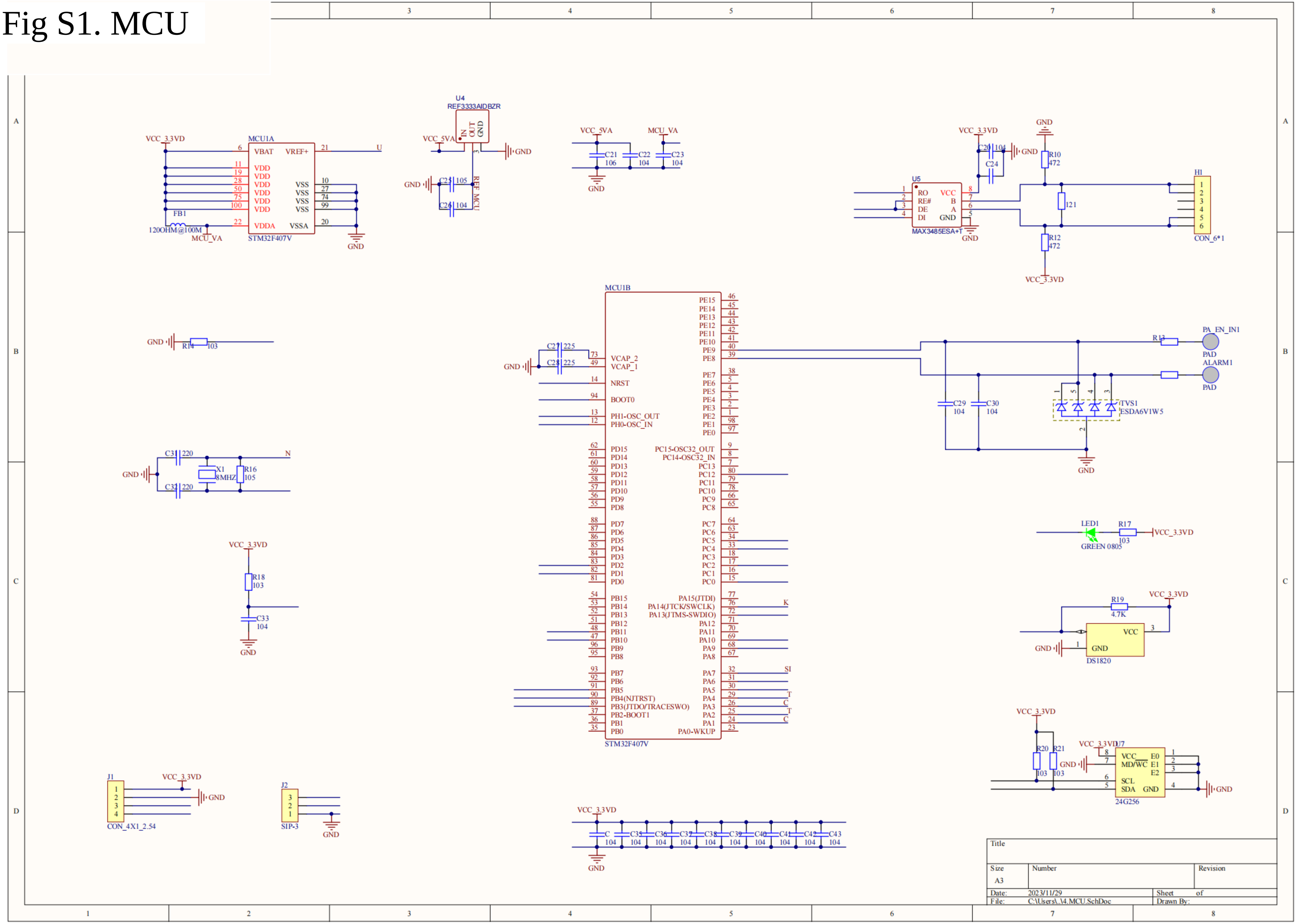

MCU1A
STM32F407V
VBAT
VREF+
VDD
VSS
VDDA
VSSA
VCC_3.3VD
FB1
120OHM@100M
MCU_VA
GND
U4
REF3333AIDBZR
IN
OUT
GND
VCC_5VA
C25 105
C26 104
REF_MCU
C21 106
C22 104
C23 104
U5
MAX3485ESA+T
RO
RE#
DE
DI
VCC
B
A
R10 472
R12 472
121
C20 104
C24
H1
CON_6*1
MCU1B
STM32F407V
VCAP_2
VCAP_1
NRST
BOOT0
PH1-OSC_OUT
PH0-OSC_IN
C27 225
C28 225
R14 103
C31 220
C32 220
X1 8MHZ
R16 105
R18 103
C33 104
PA_EN_IN1
ALARM1
PAD
R13
C29 104
C30 104
TVS1 ESDA6V1W5
LED1
GREEN 0805
R17 103
R19 4.7K
DS1820
R20 103
R21 103
U7
24G256
VCC
MD/WC
SCL
SDA
E0
E1
E2
J1
CON_4X1_2.54
J2
SIP-3
Title
Size
A3
Number
Revision
Date: 2023/11/29
File: C:\Users\..\4.MCU.SchDoc
Sheet of
Drawn By:

Fig S2. Preamplifier module-power supply and control section

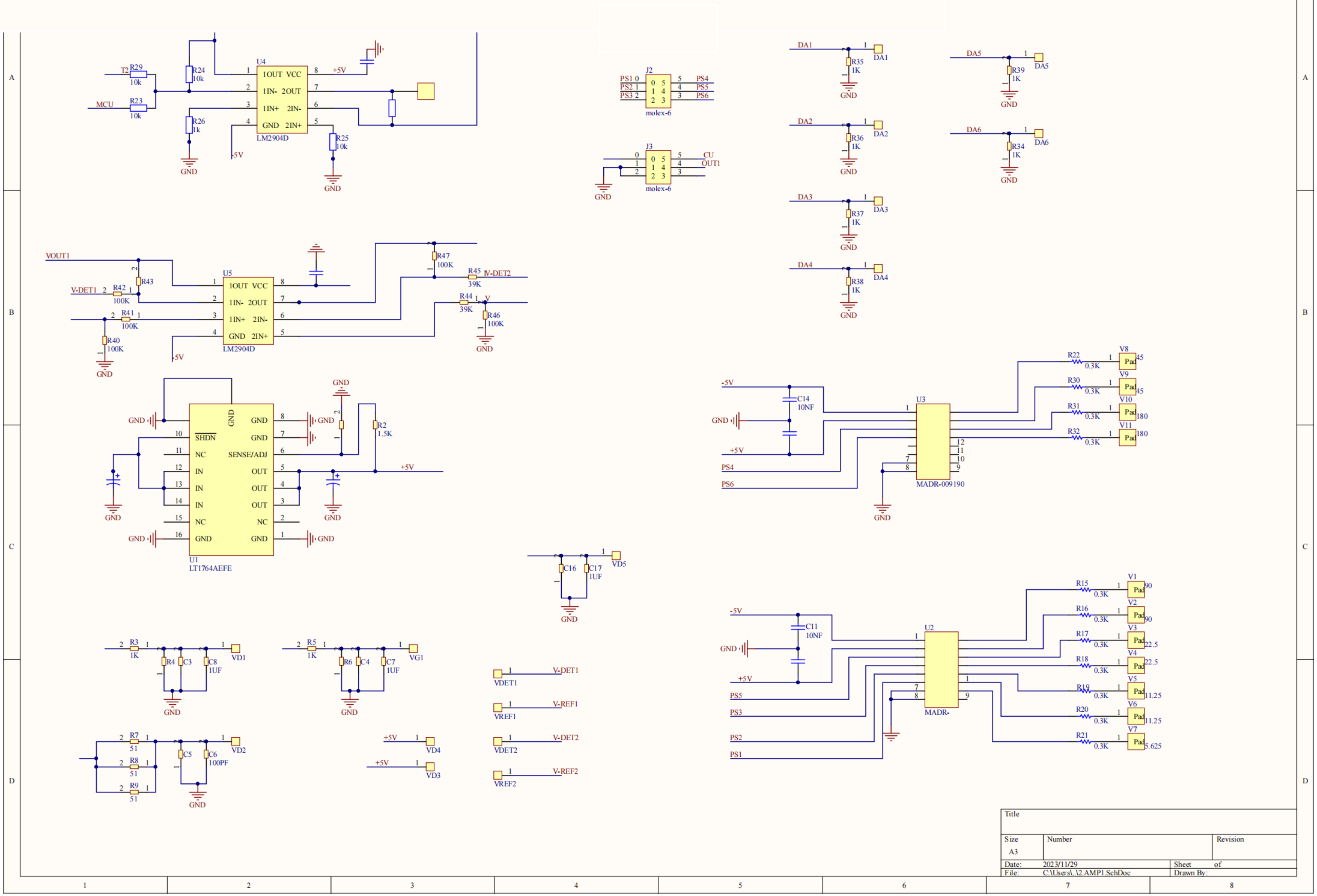

Fig S3. Drive module-power supply and control section

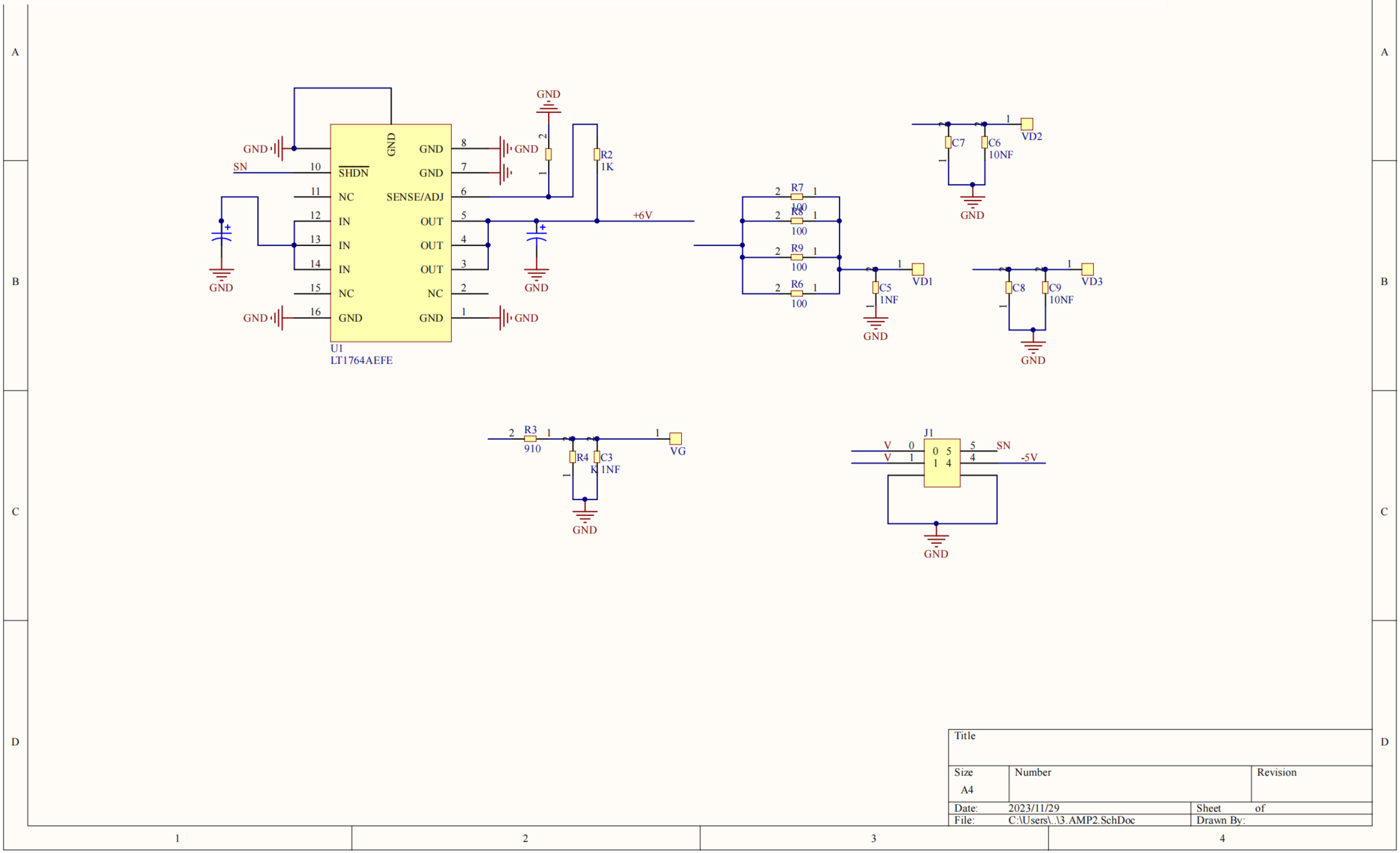

Fig S4. Amplifier-con

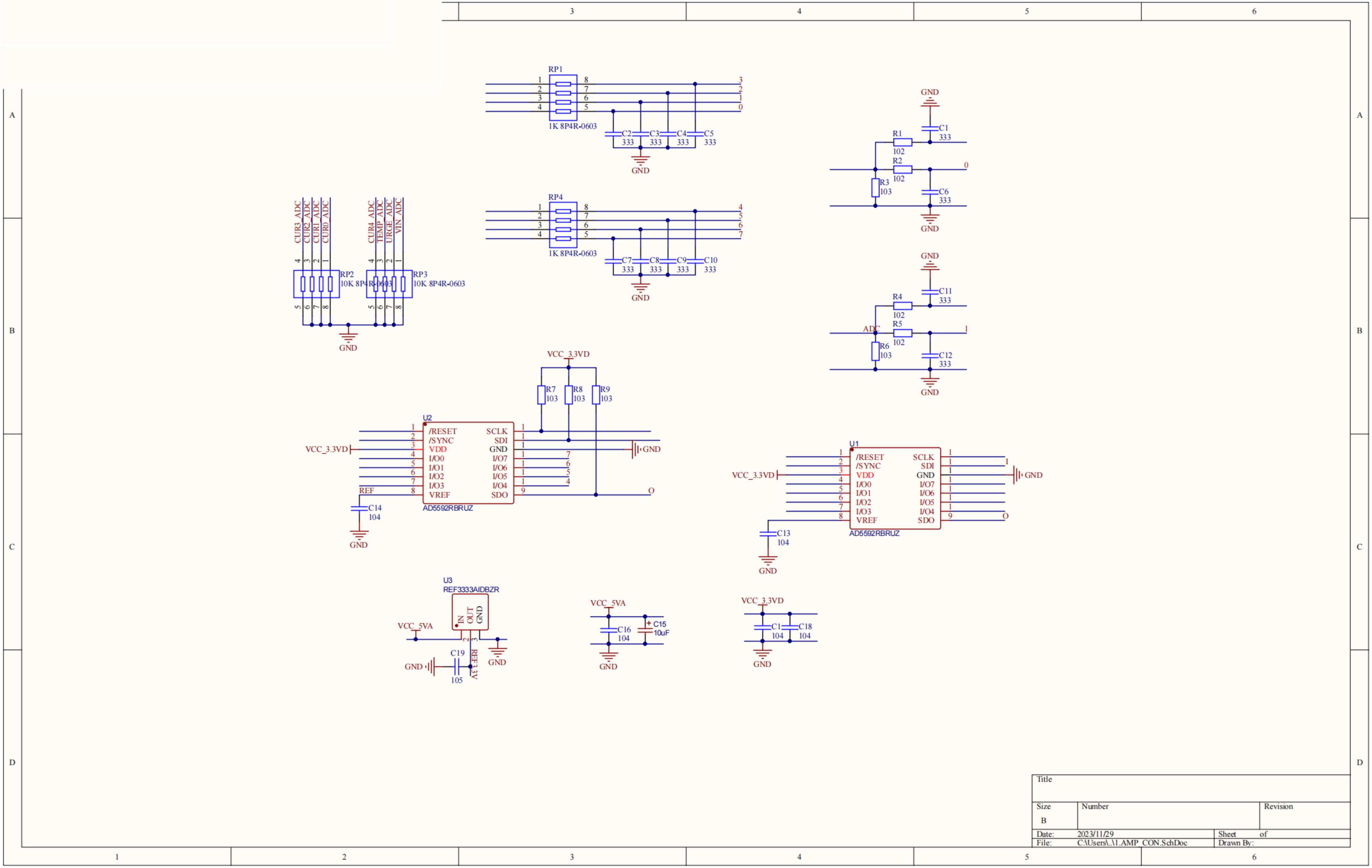

Fig S5. Amplifier-total power control

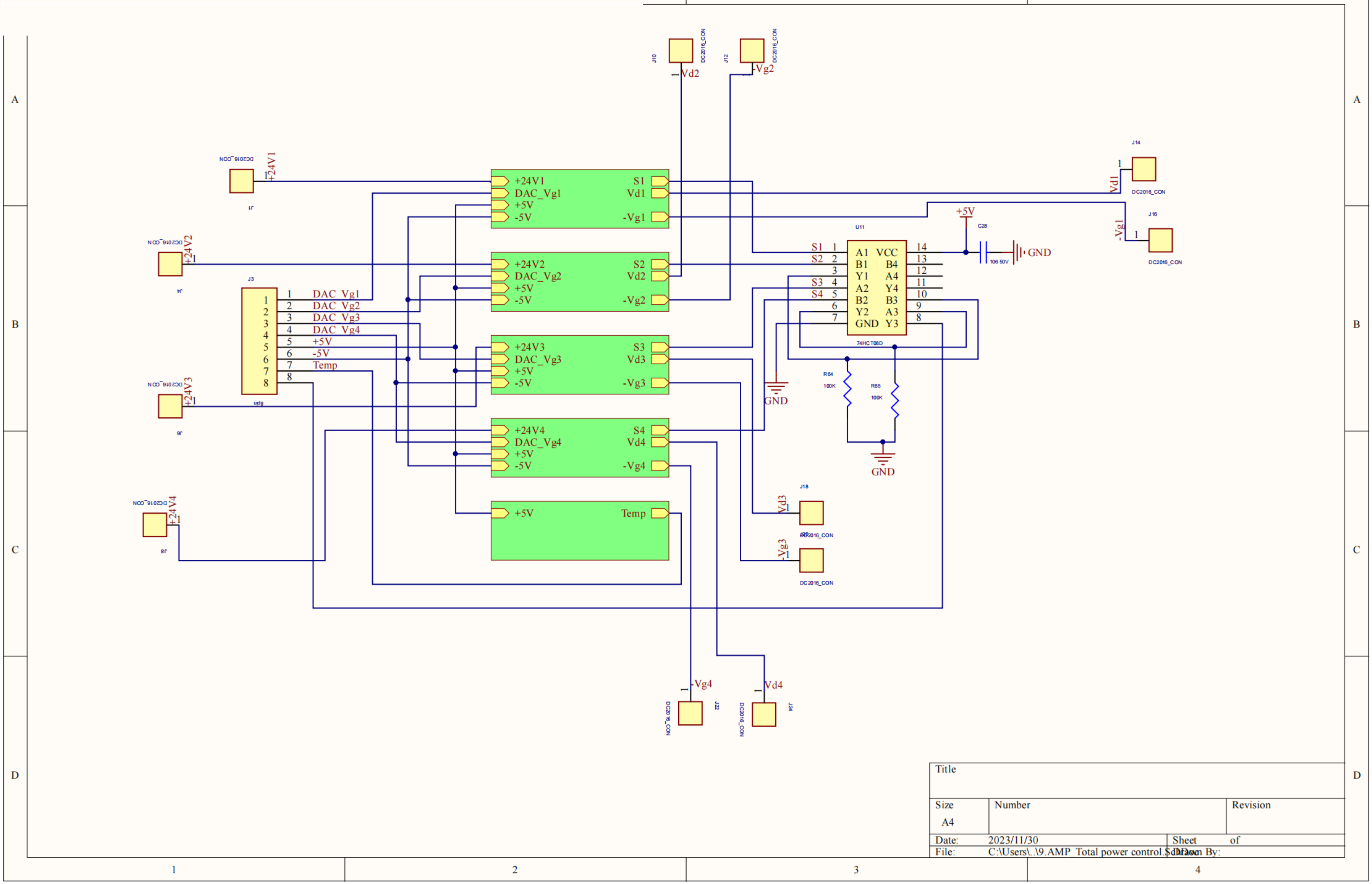

Fig S6. Function module

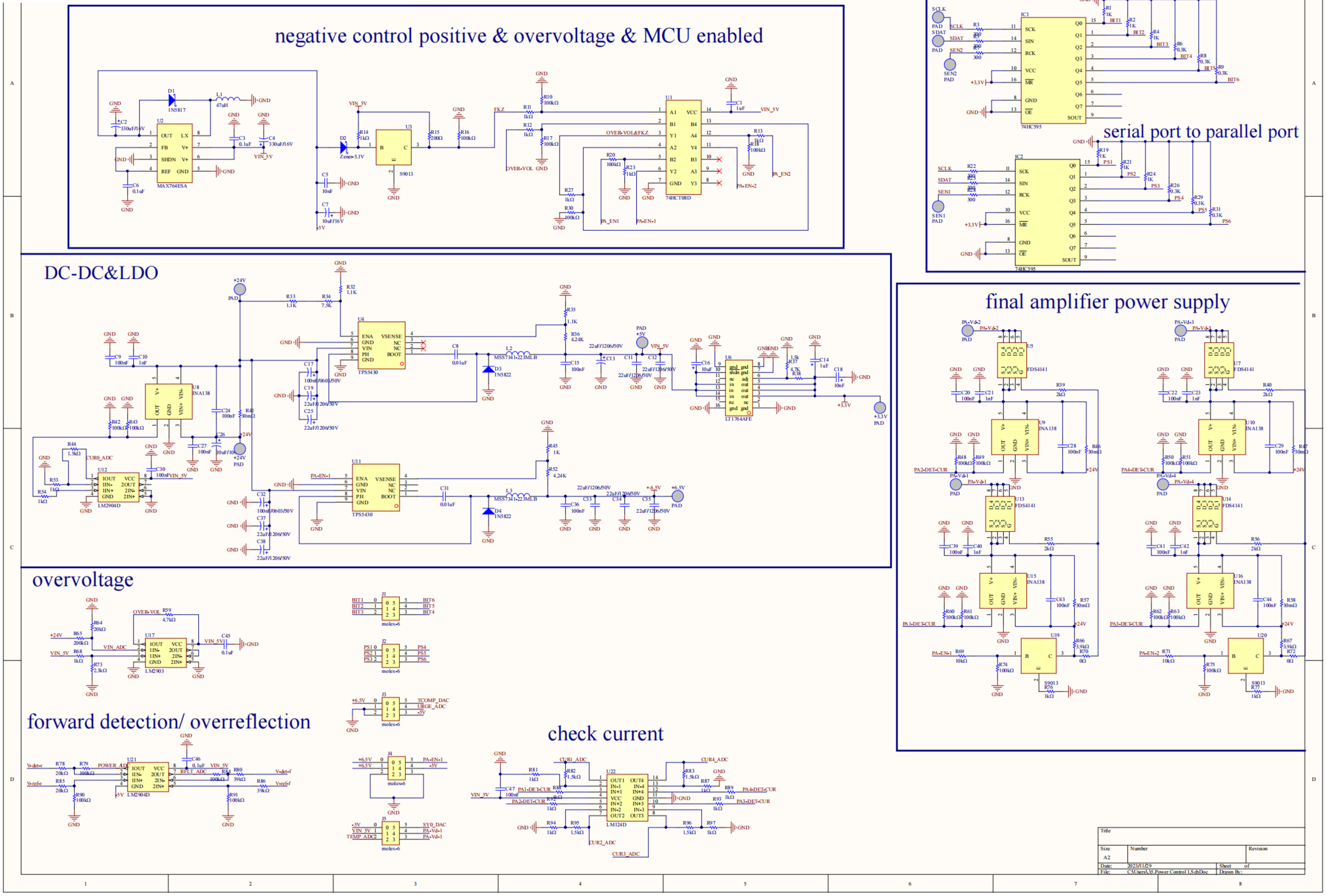

Fig S7. Power control

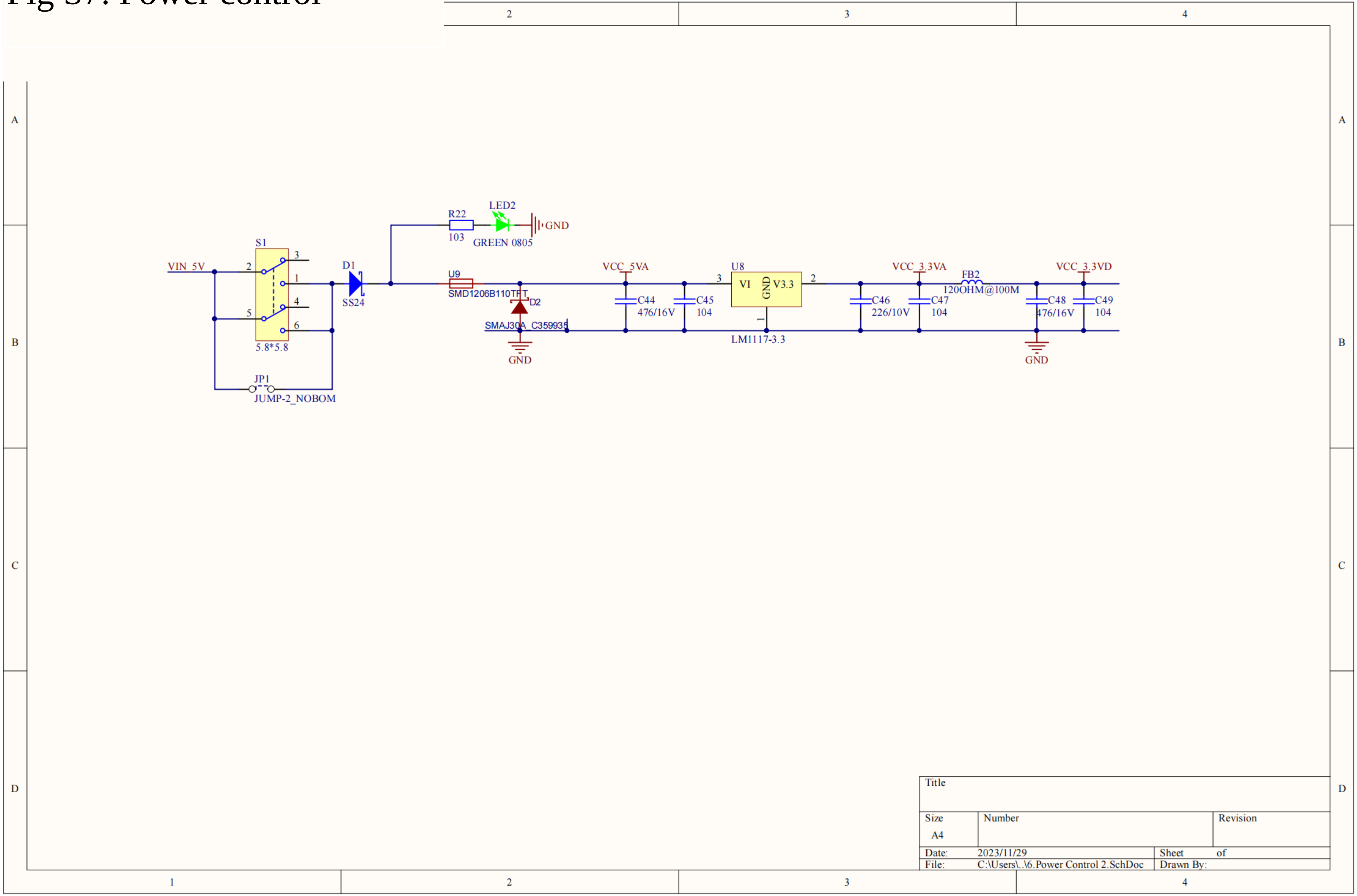

Fig S8. Amplifier-detection

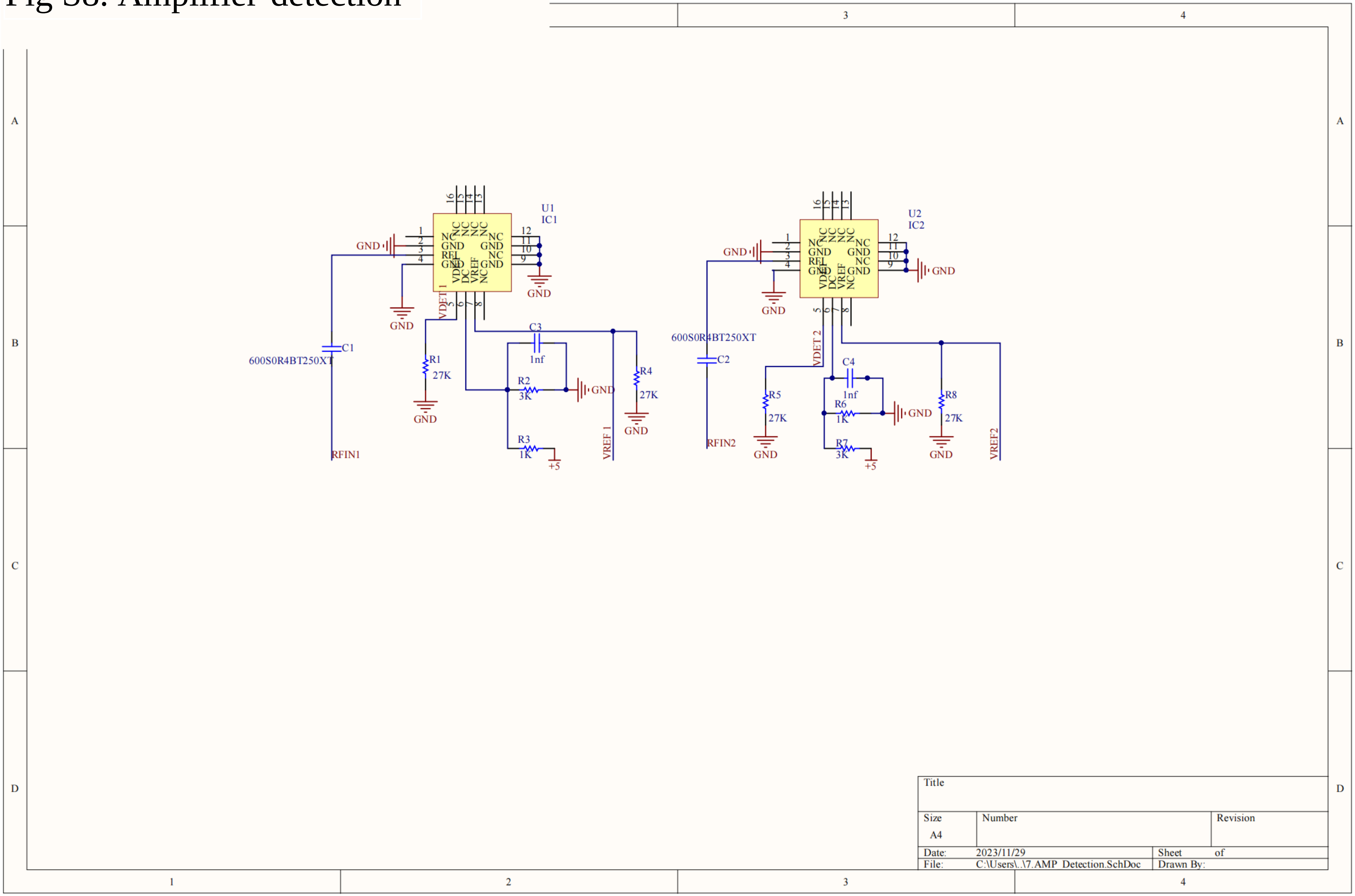

Fig S9. Amplifier-temperature

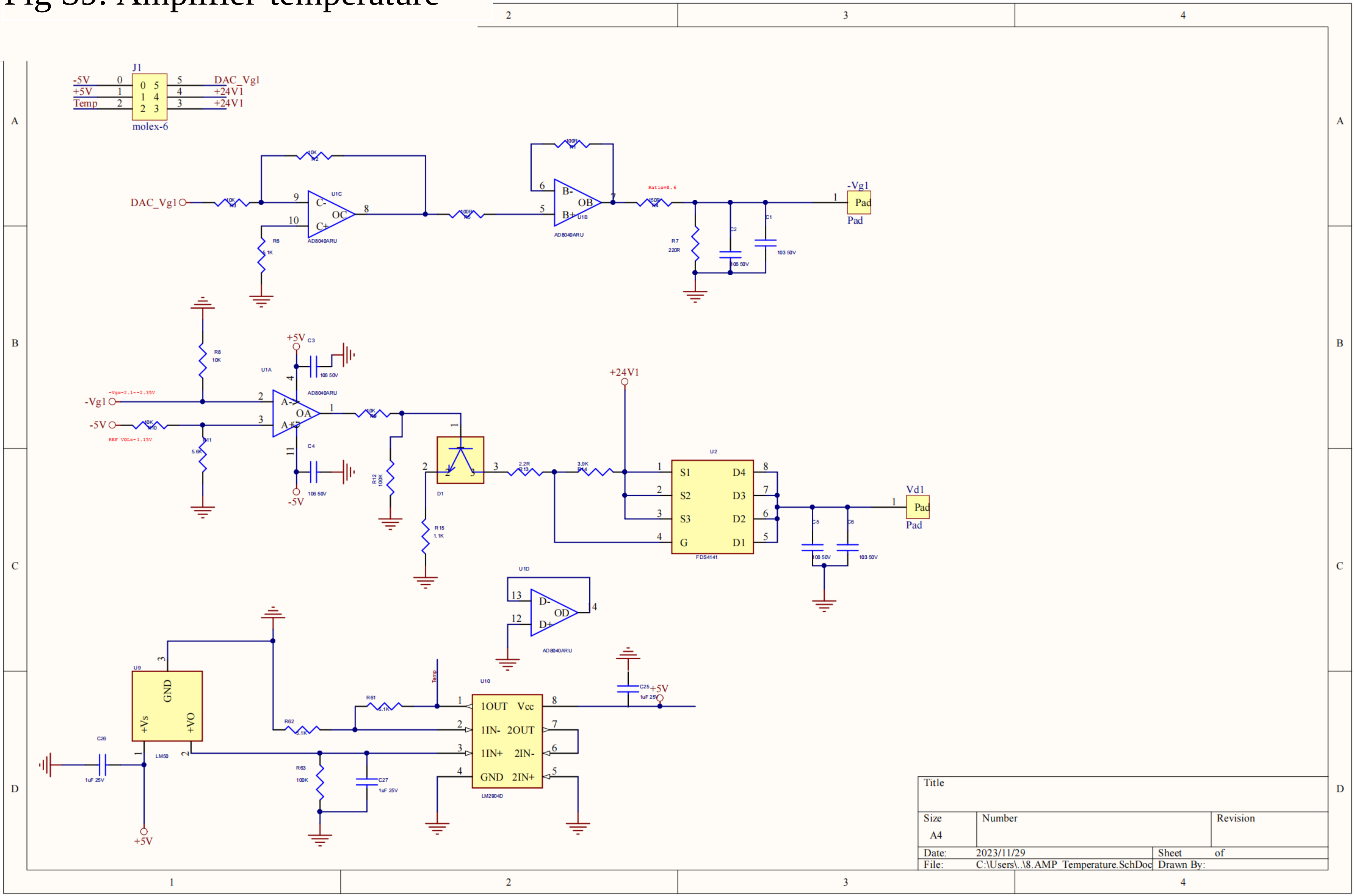

Fig S10. Ku-band high amplifier layout

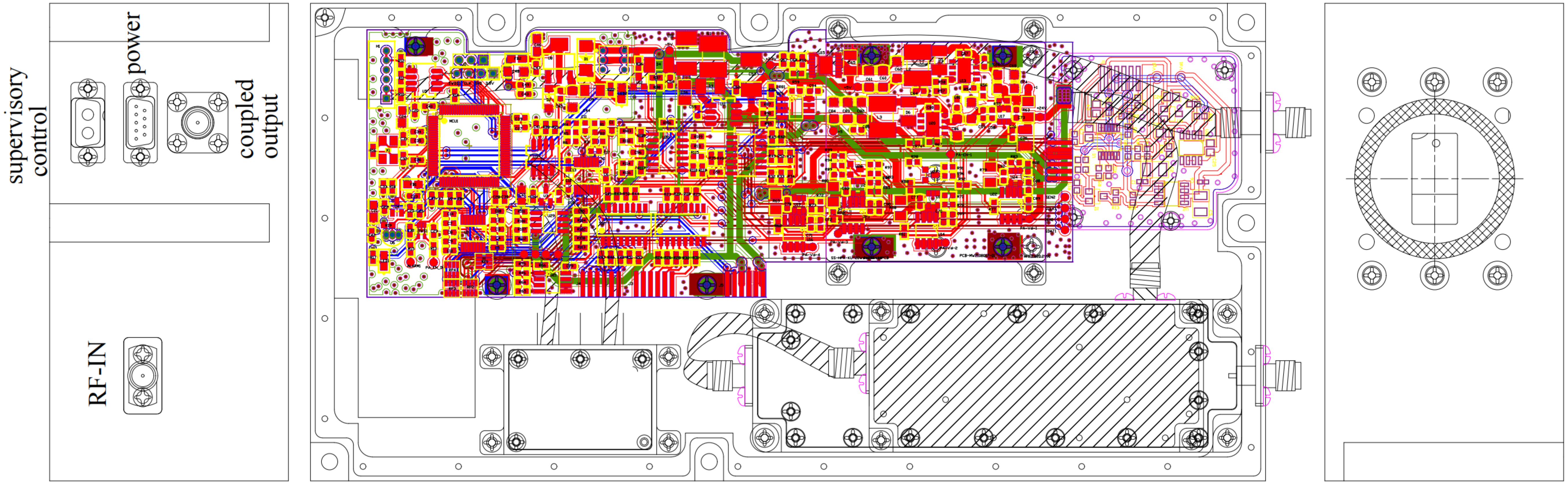

Fig S11. Layout of preamplifier module

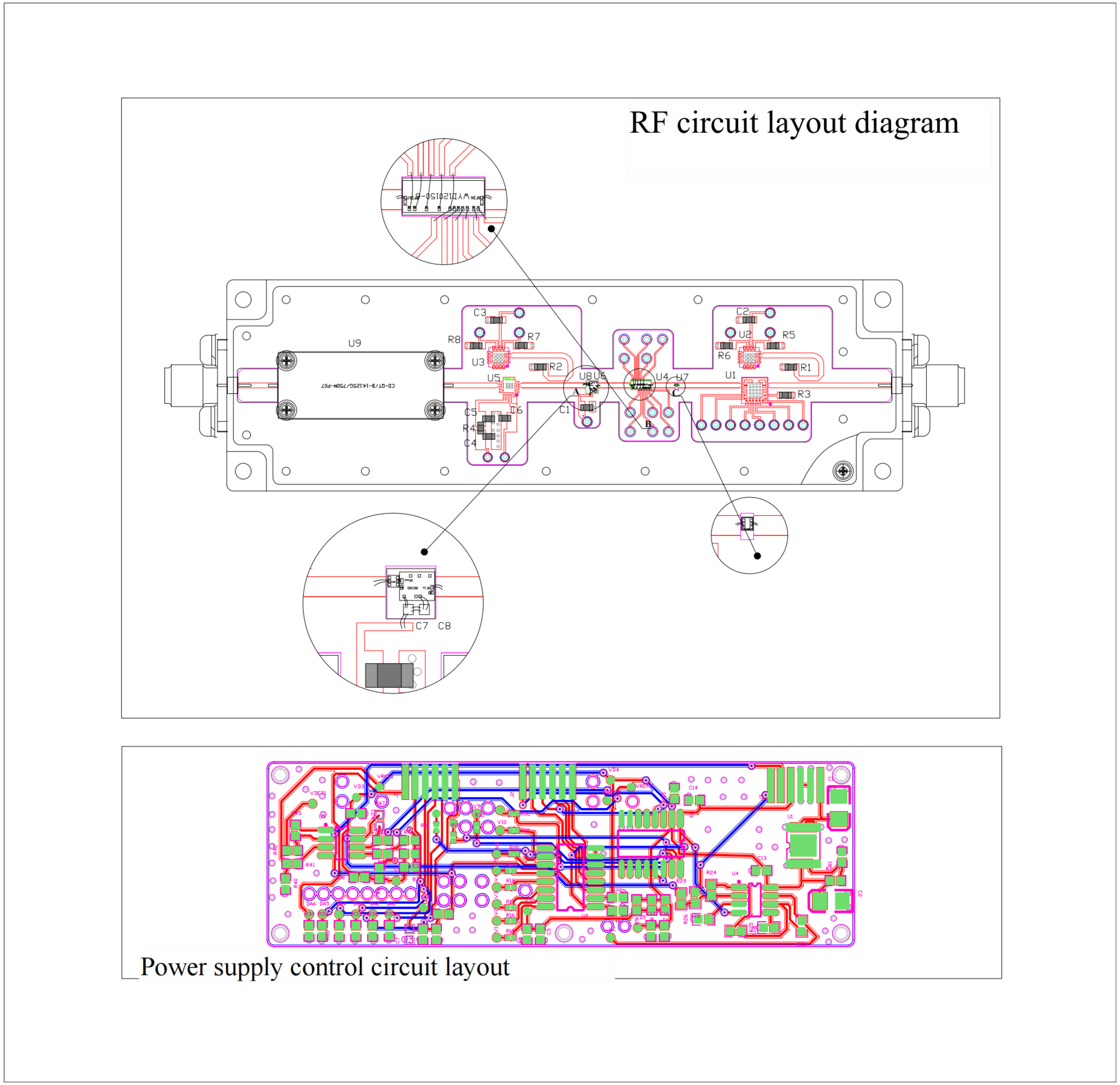

Fig S12. Drive amplifier module layout

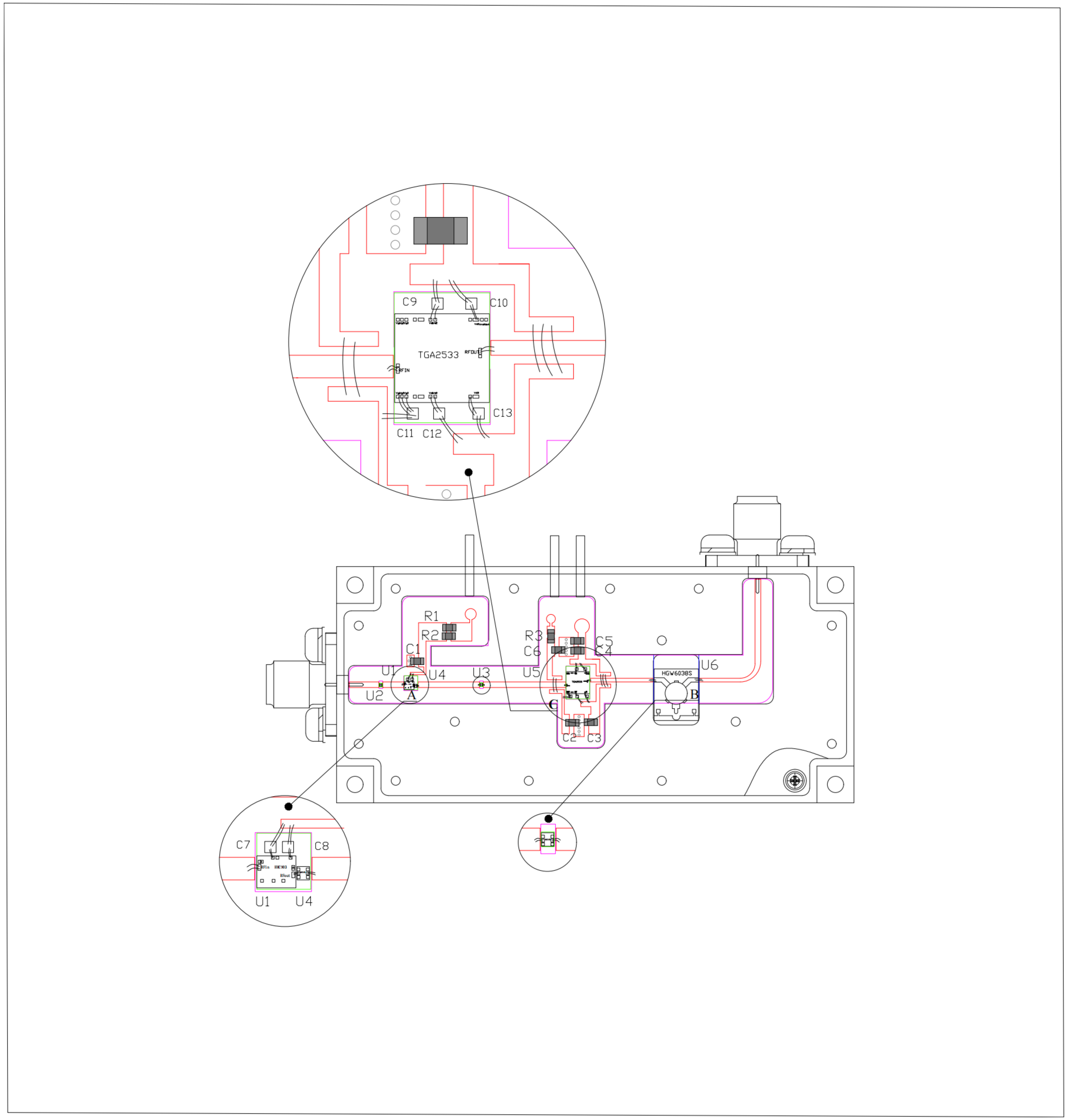

Fig S13. Layout of the amplification module

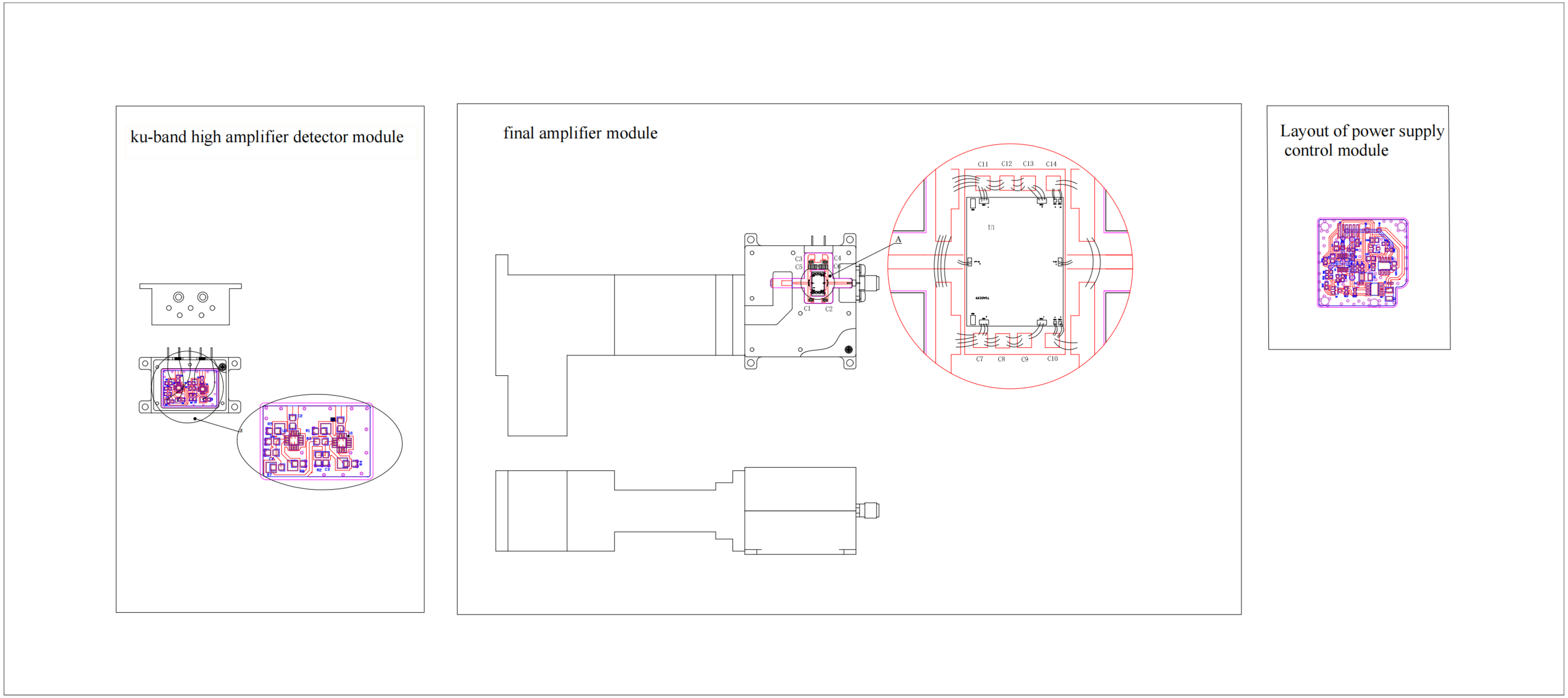

Fig S14. Amplifier-PCB

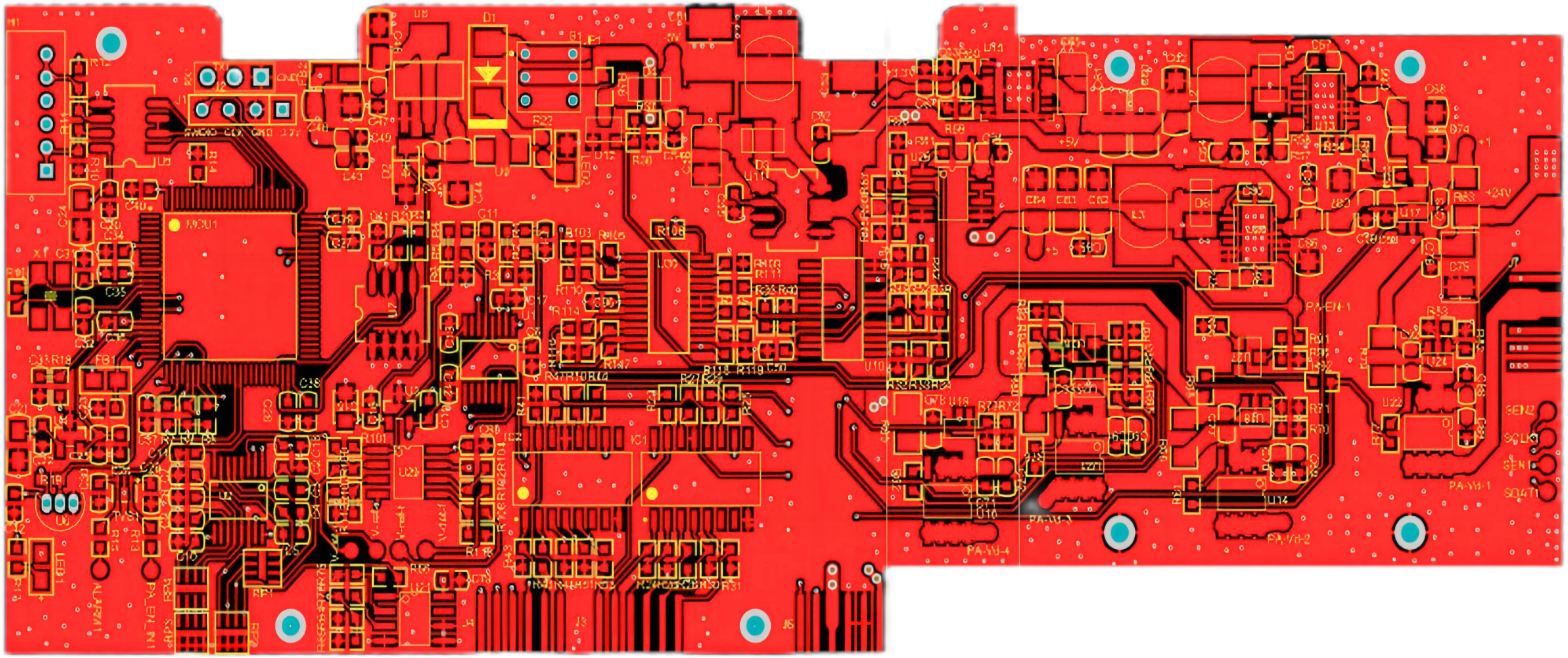

Fig S15. Preamplifier module-power supply and control section

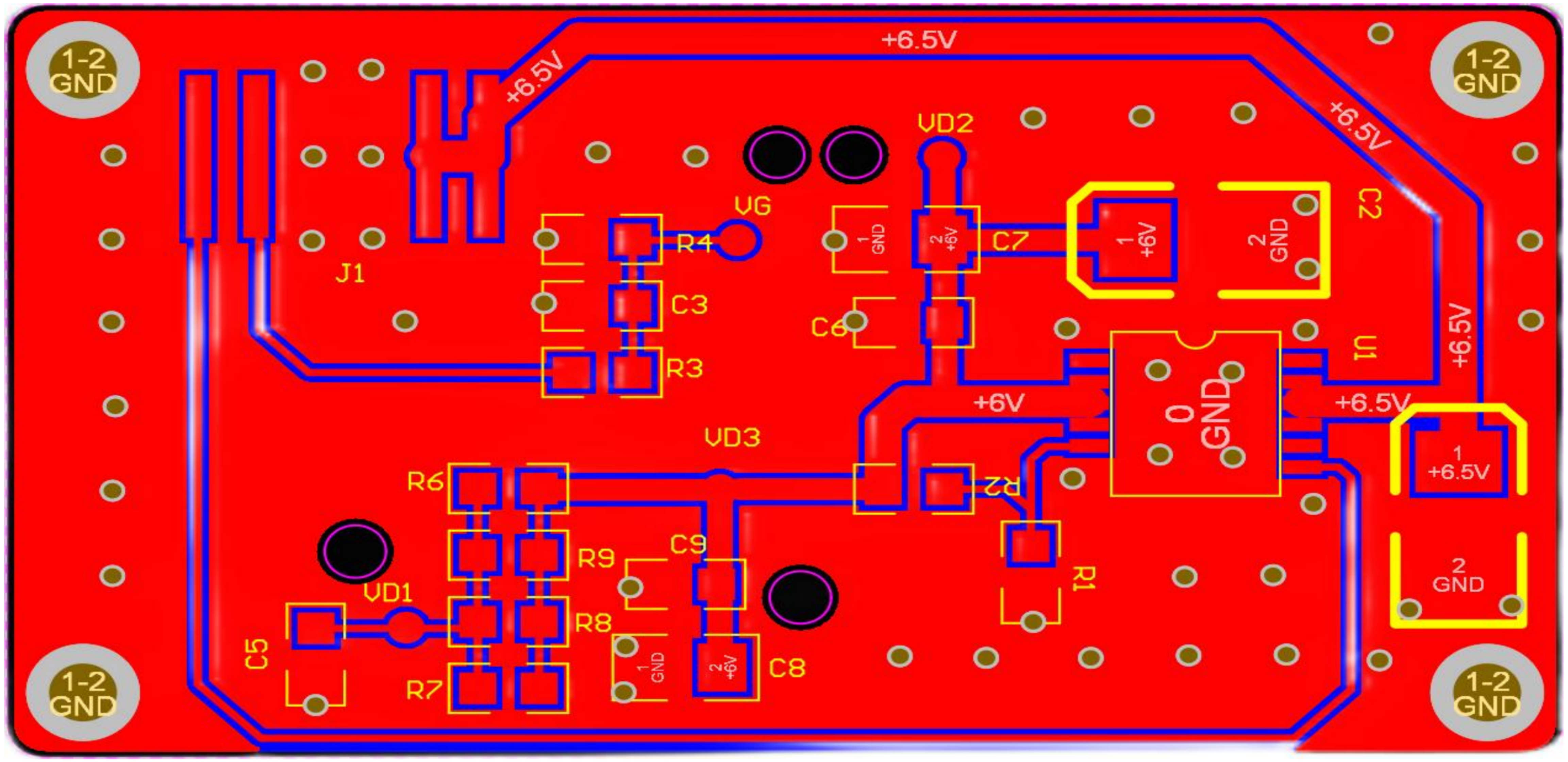

Fig S16. Drive amplifier module-power supply and control section

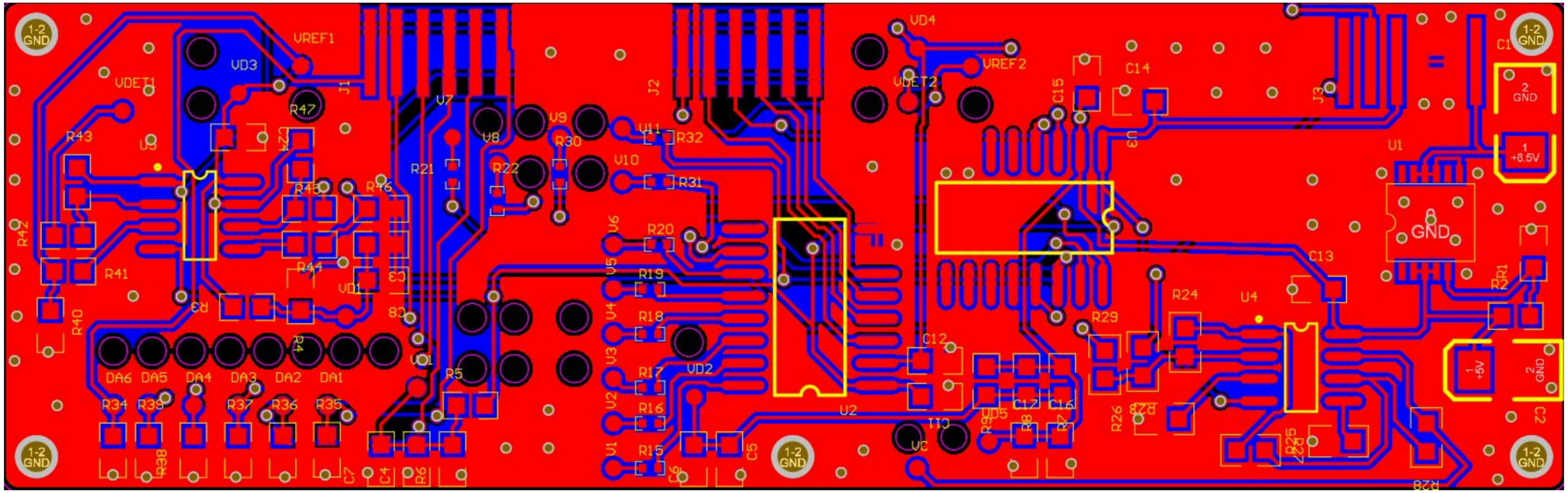

Fig S17. Amplifier-detection

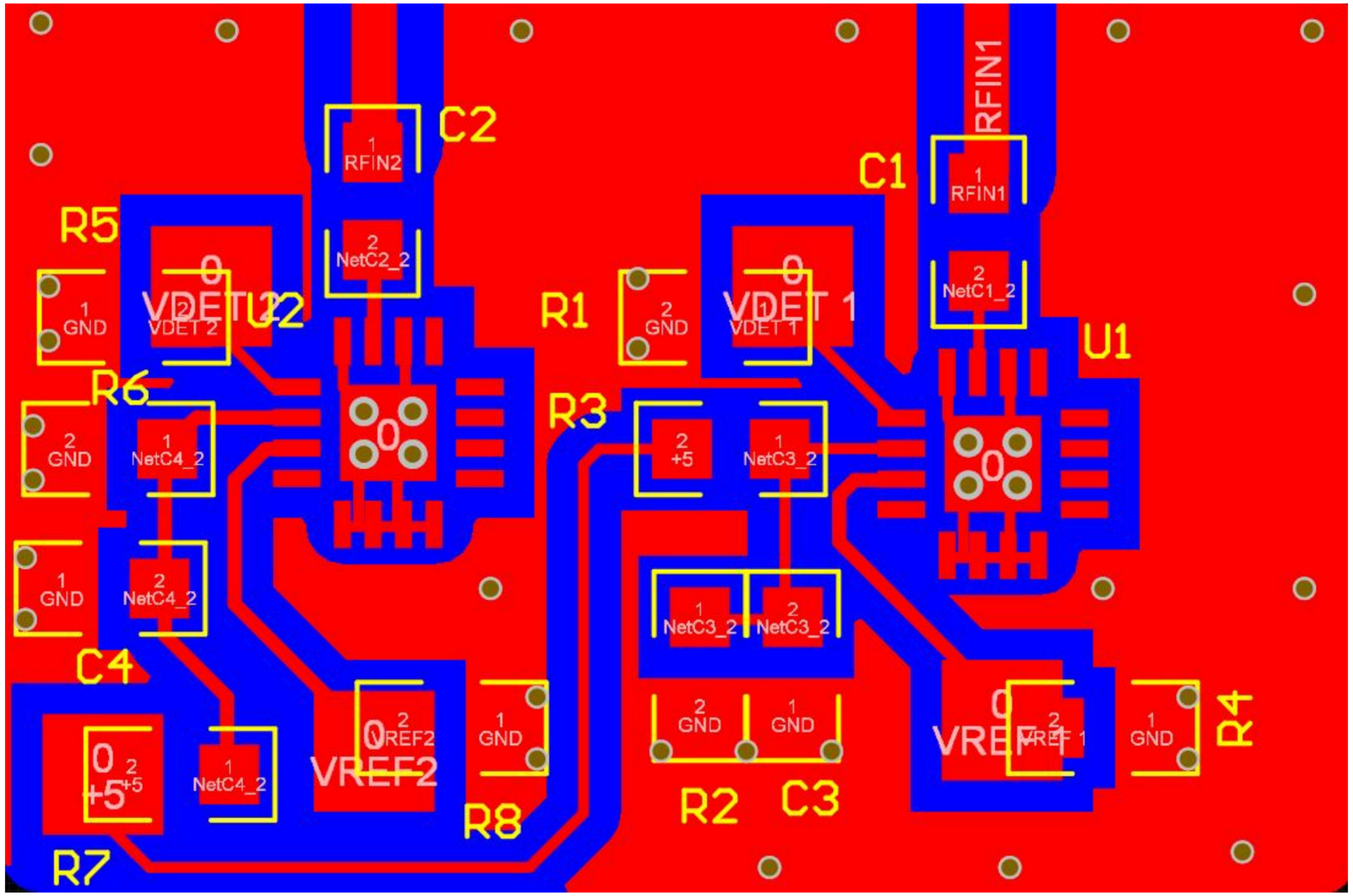

Fig S18. Amplifier-temperature

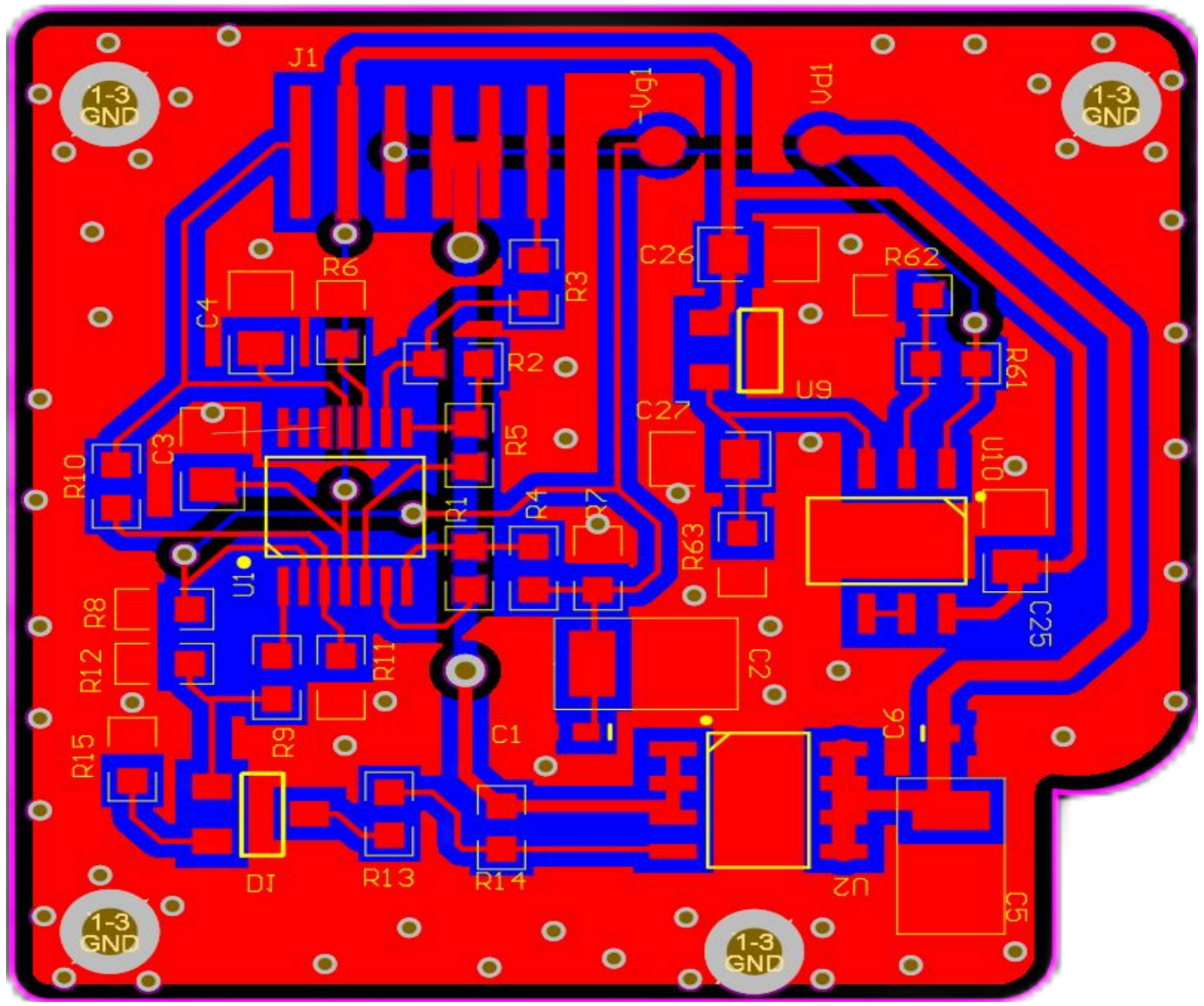

Fig S19. Preamplifier module RF link

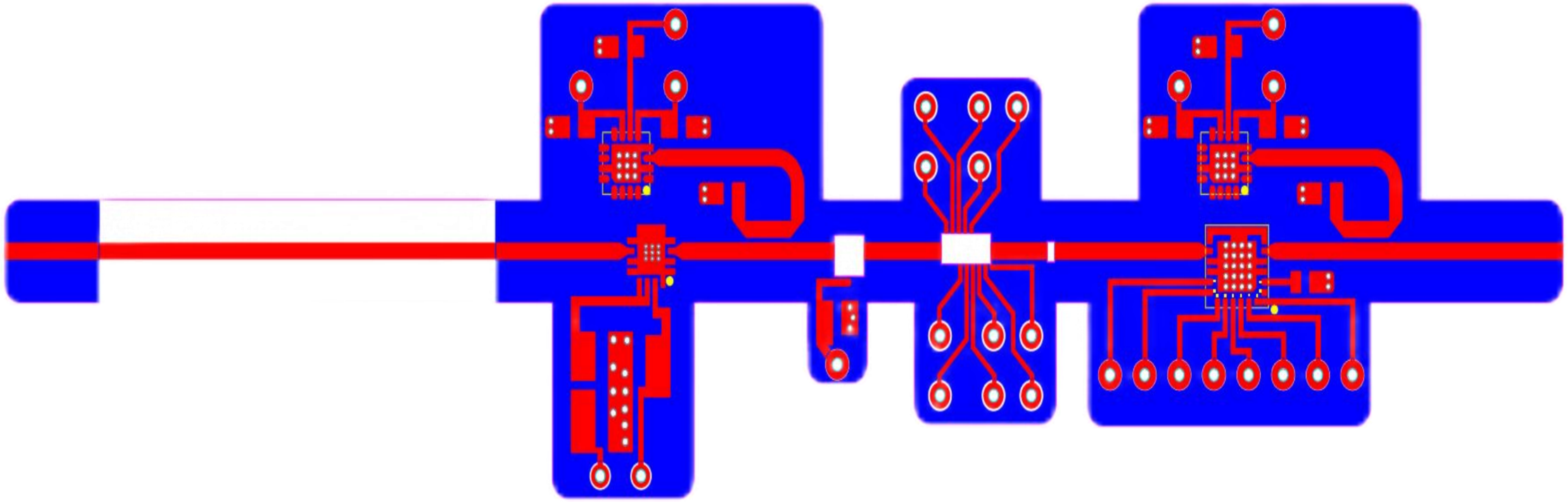

Fig S20. Drive amplifier module RF link

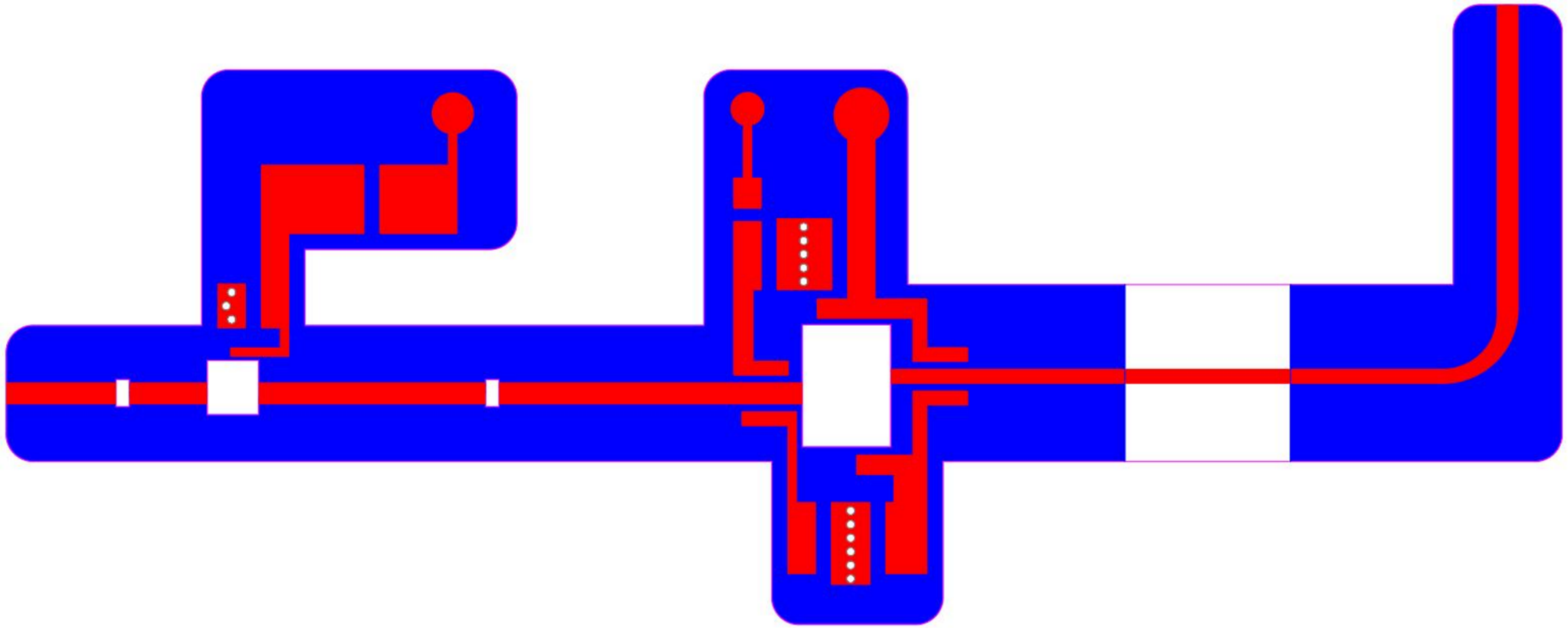

Fig S21. Final amplifier module RF link

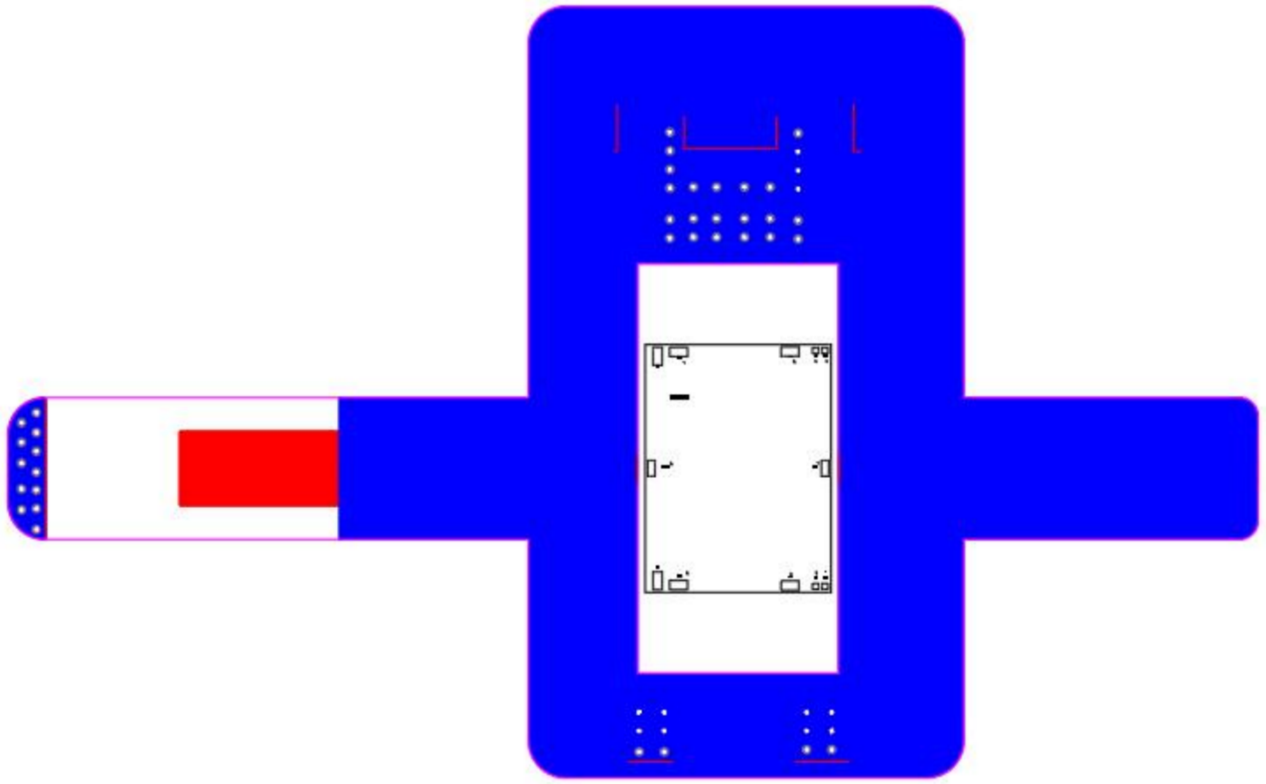

Fig S22. Ku-band power amplifier chanmber

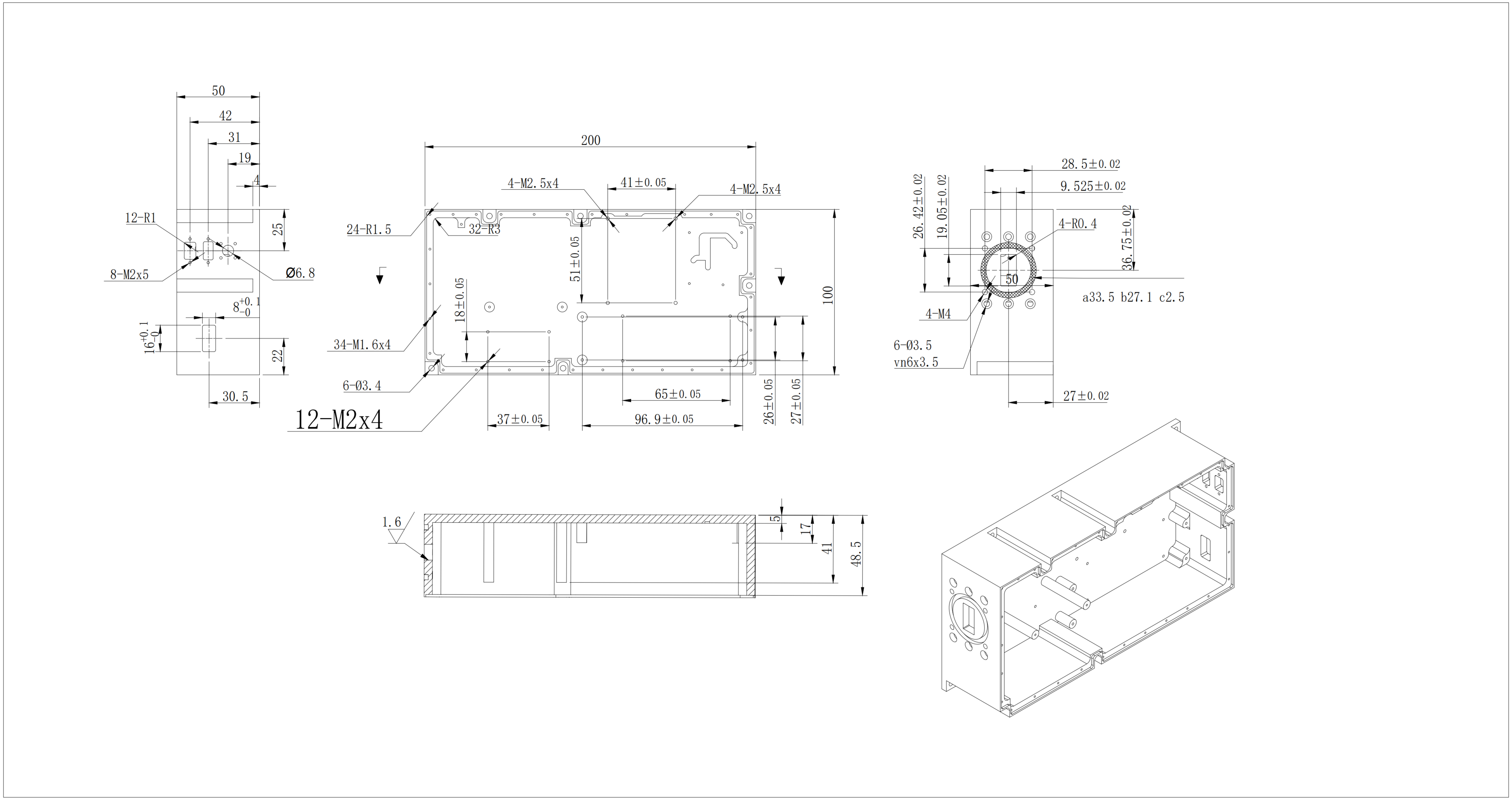

Fig S23. Ku-band high amplifier chanmber cover plate

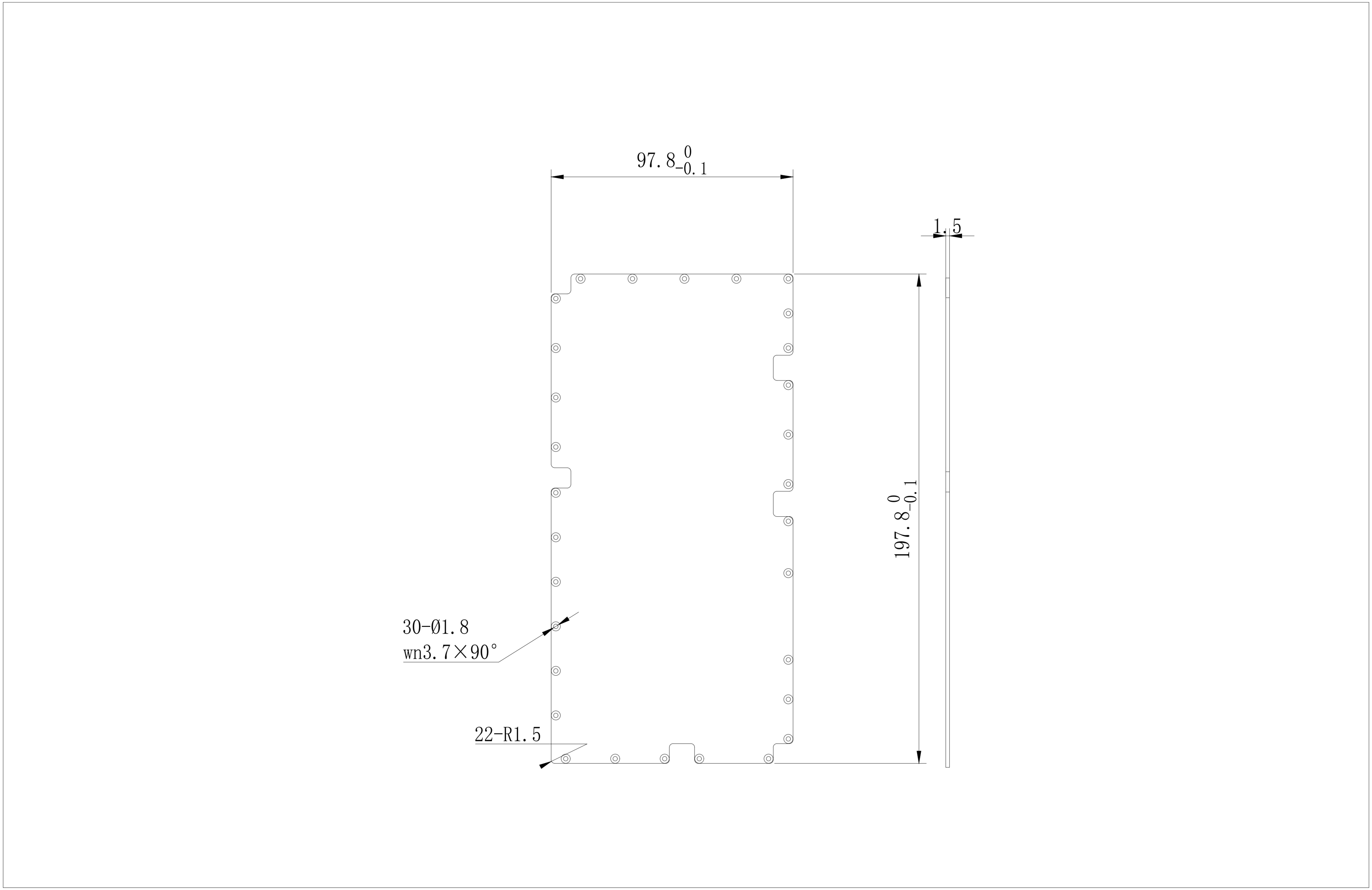

Fig S24. Ku-band power amplifier preamplifier module-cavity

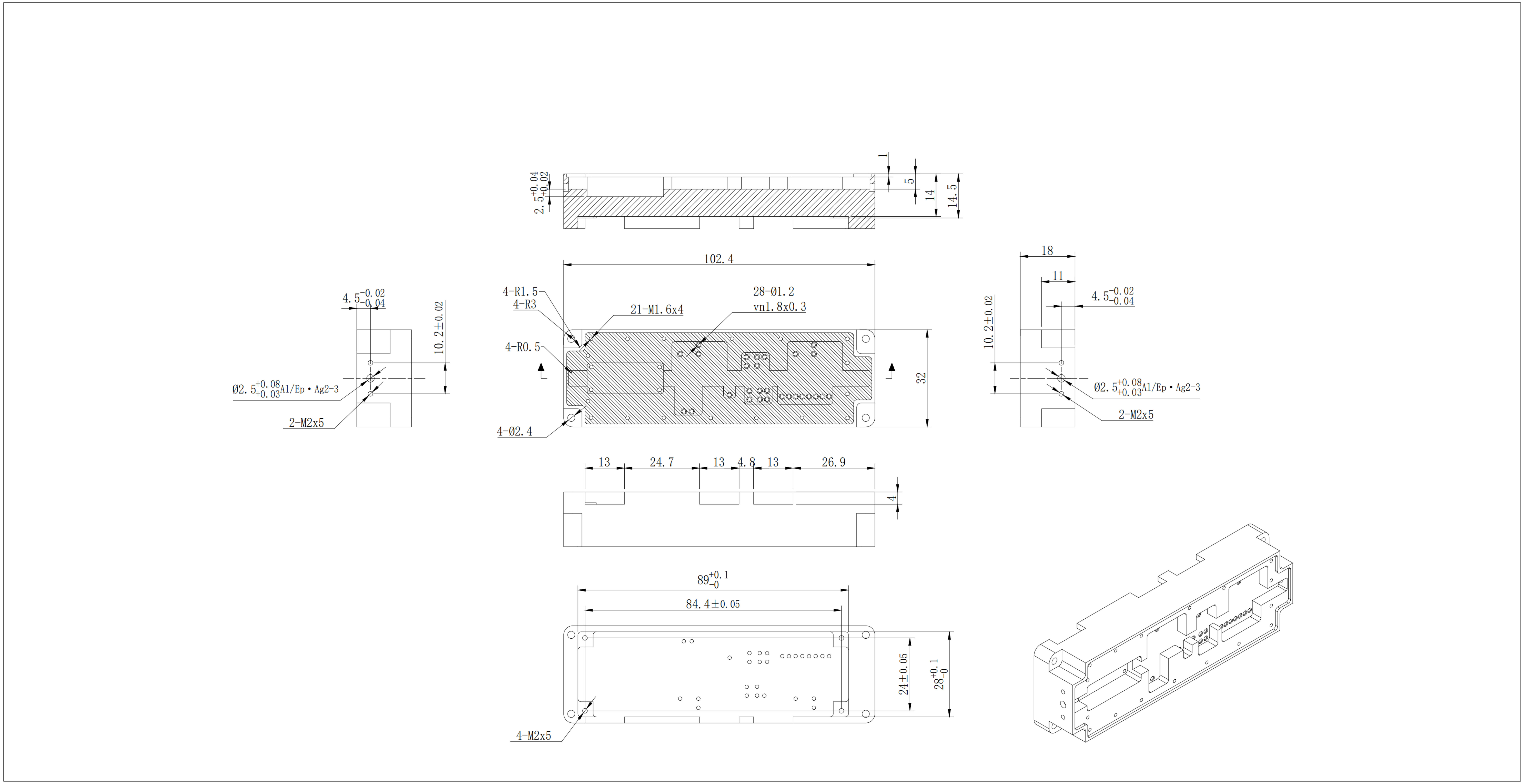

Fig S25. Ku-band high amplifier preamplifier module-cover plate

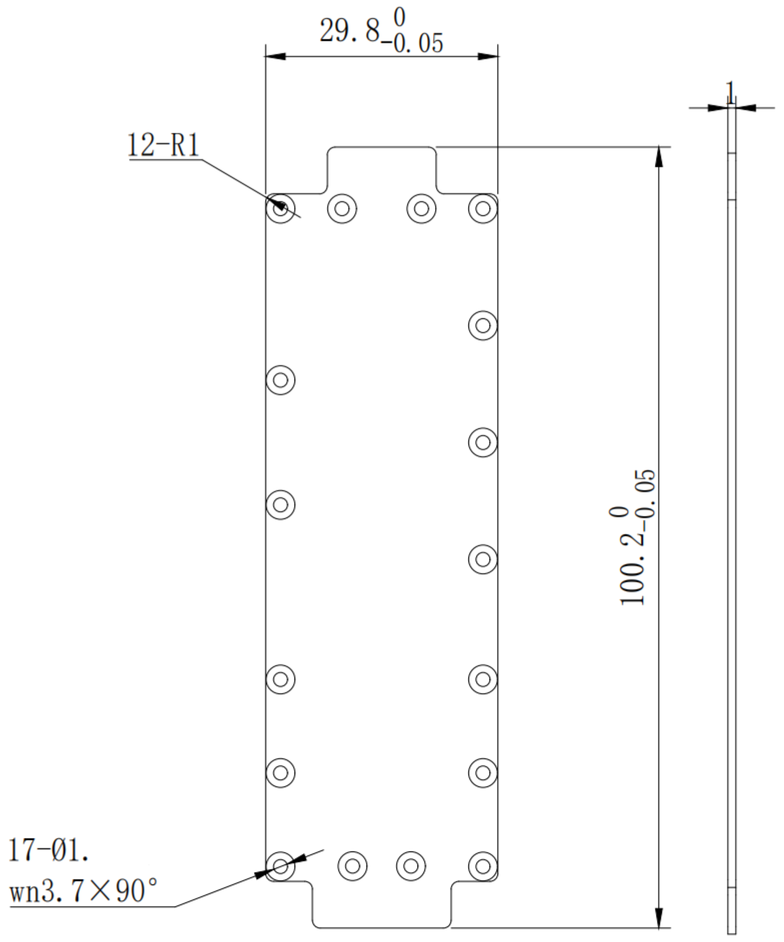

Fig S26. Ku-band high power amplifier drive amplifier module-cavity

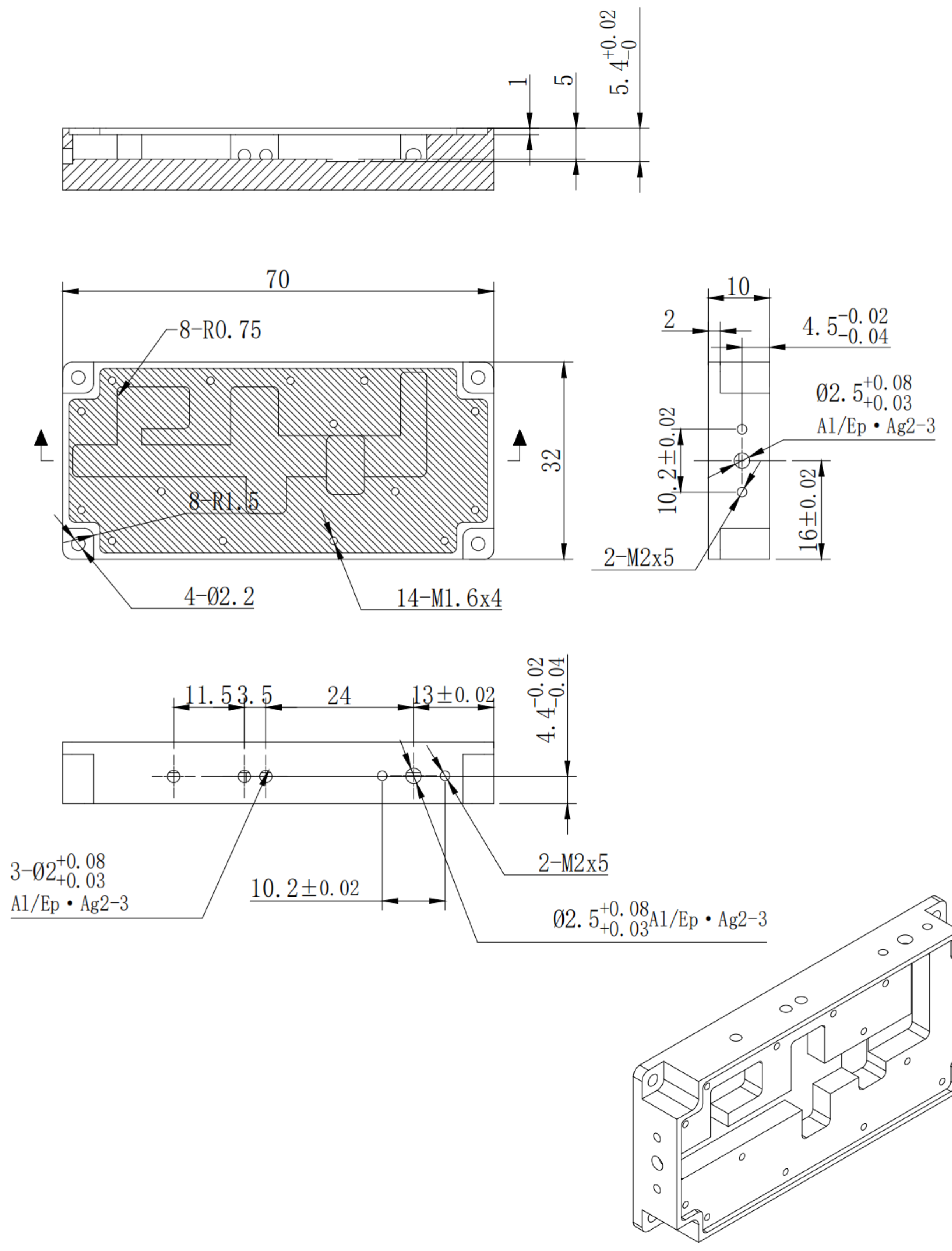

Fig S27. Ku-band high amplifier module-cover plate

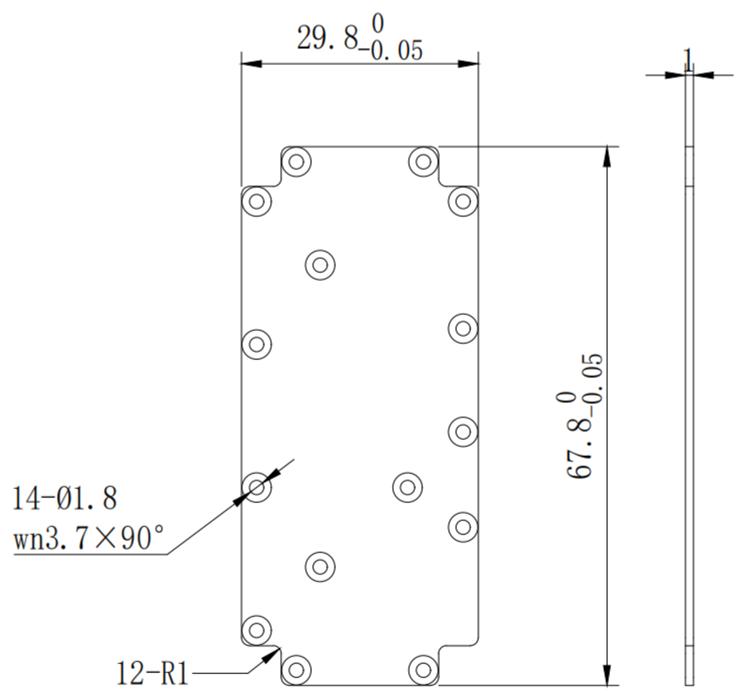

Fig S28. Ku-band high amplifier final stage amplifier module-upper chamber

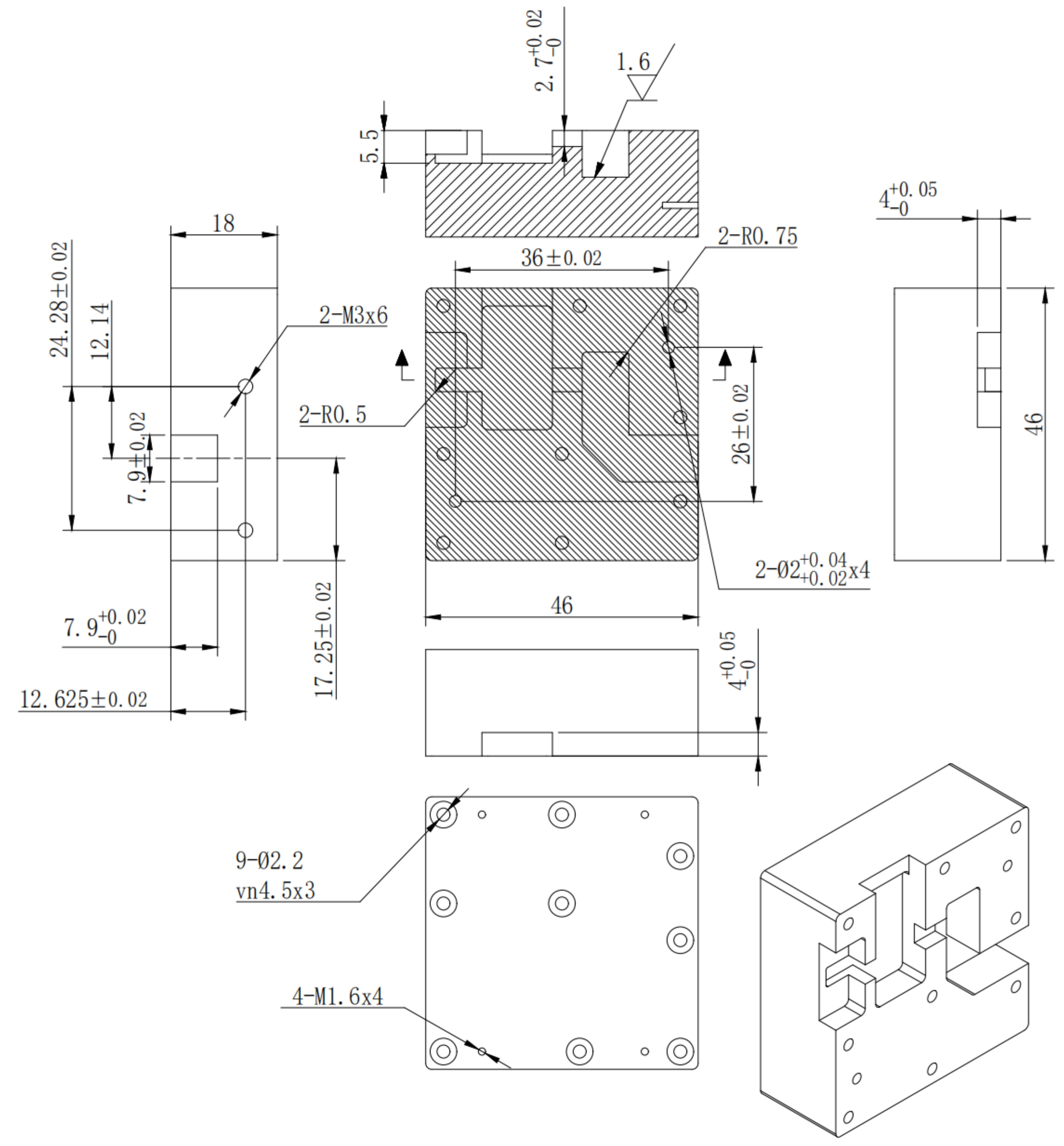

Fig S29. Ku-band high amplifier final stage amplifier module-lower chanmber

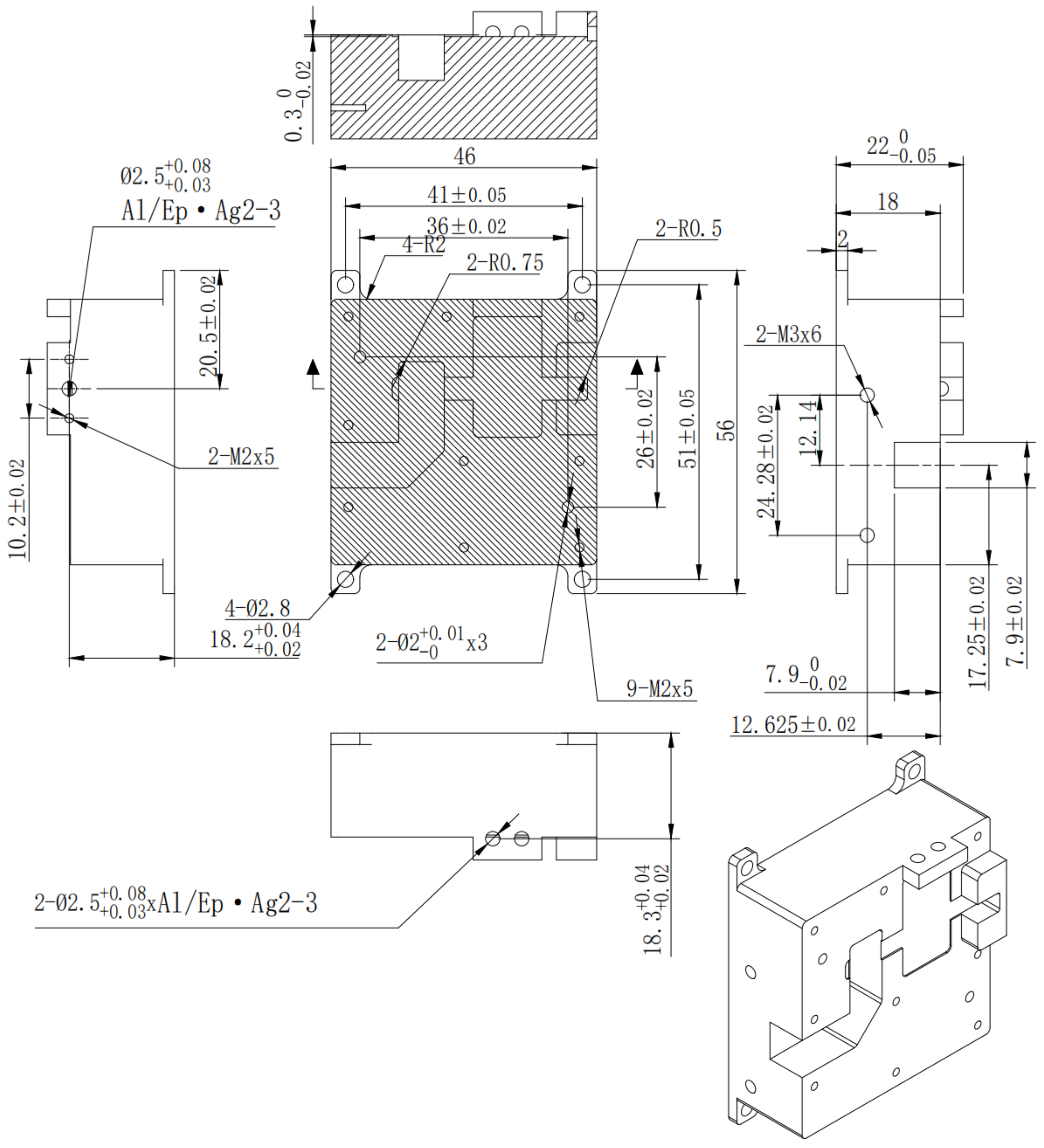

Fig S30. Ku-band high amplifier detector module-cavity

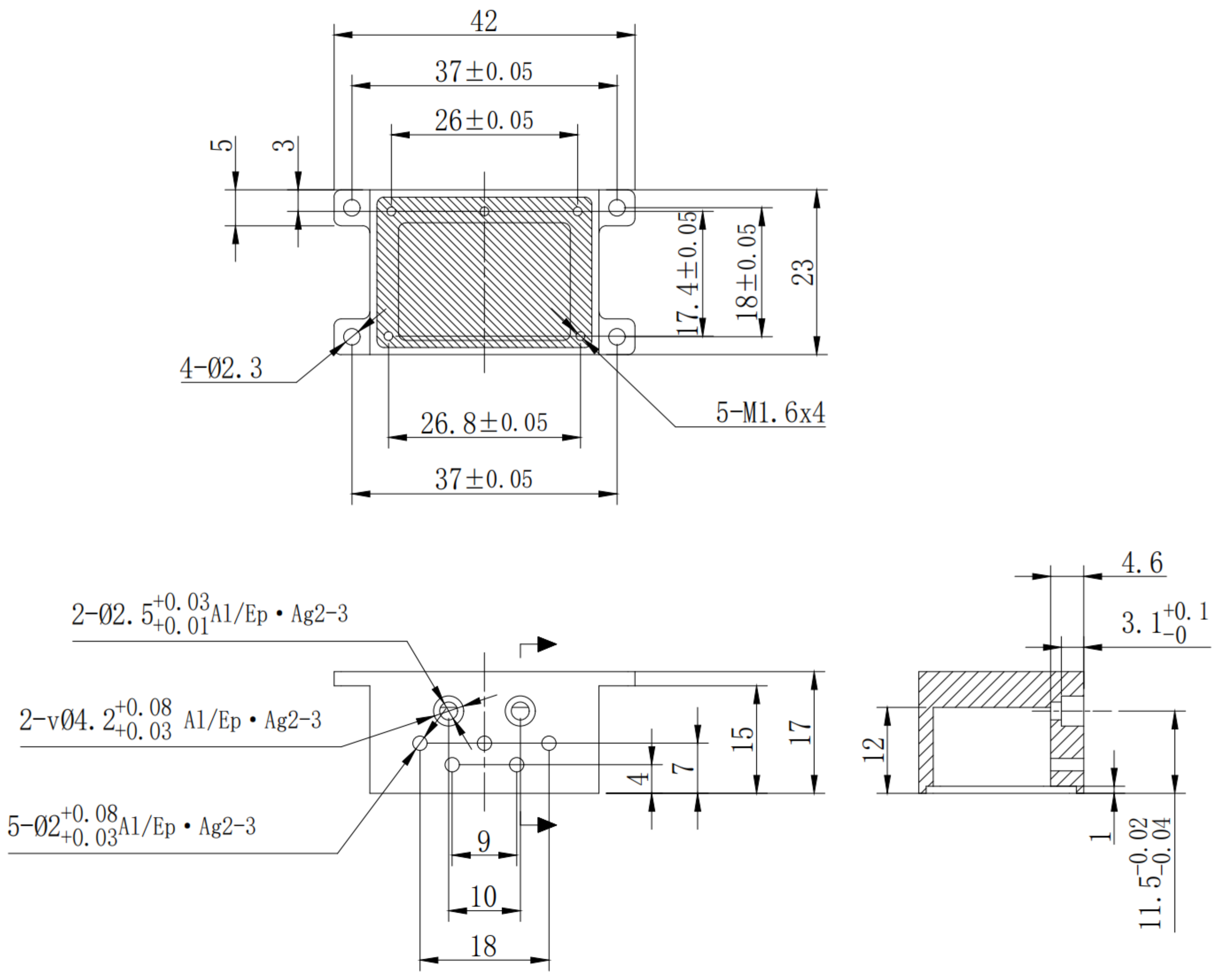

Fig S31. Ku-band high amplifier detector module-cover plate

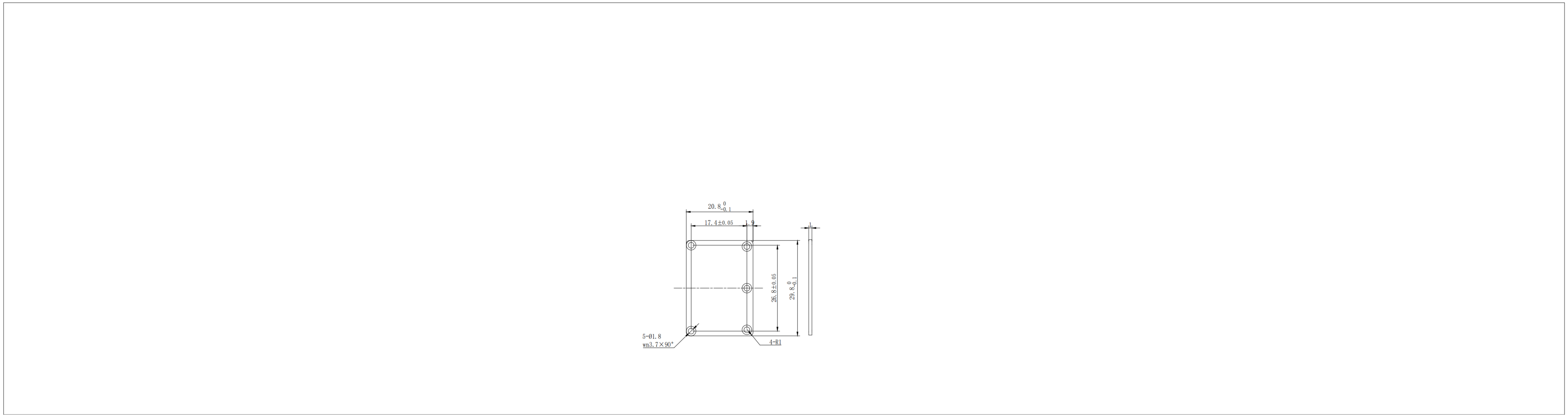

Fig S32. Preamplifier module-power supply and control section

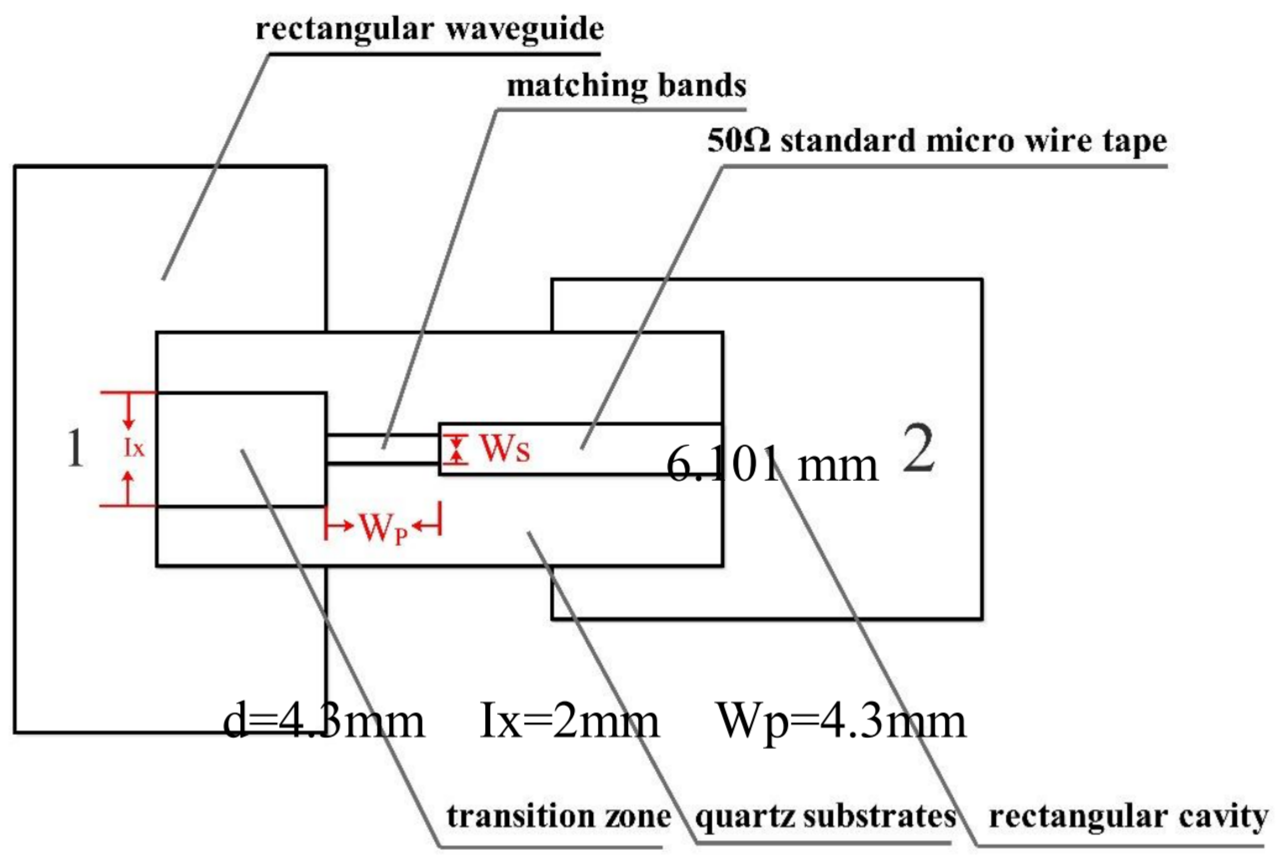

Supplement: S1 Raw images — (PDF) [file pone.0300616.s001.pdf]
